# Prediction of treatment response to antipsychotic drugs for precision medicine approach to schizophrenia: randomized trials and multiomics analysis

**DOI:** 10.1186/s40779-023-00459-7

**Published:** 2023-06-02

**Authors:** Liang-Kun Guo, Yi Su, Yu-Ya-Nan Zhang, Hao Yu, Zhe Lu, Wen-Qiang Li, Yong-Feng Yang, Xiao Xiao, Hao Yan, Tian-Lan Lu, Jun Li, Yun-Dan Liao, Zhe-Wei Kang, Li-Fang Wang, Yue Li, Ming Li, Bing Liu, Hai-Liang Huang, Lu-Xian Lv, Yin Yao, Yun-Long Tan, Gerome Breen, Ian Everall, Hong-Xing Wang, Zhuo Huang, Dai Zhang, Wei-Hua Yue

**Affiliations:** 1 Institute of Mental Health, Peking University Sixth Hospital, Beijing, 100191 China; 2 National Clinical Research Center for Mental Disorders, Peking University Sixth Hospital, Beijing, 100191 China; 3 NHC Key Laboratory of Mental Health and Research Unit of Diagnosis and Treatment of Mood Cognitive Disorder (2018RU006), Chinese Academy of Medical Sciences, Beijing, 100191 China; 4 Peking University Huilongguan Clinical Medical School, Beijing Huilongguan Hospital, Beijing, 100096 China; 5 Department of Psychiatry, Jining Medical University, Jining, 272067 Shandong China; 6 Henan Key Lab of Biological Psychiatry, The Second Affiliated Hospital of Xinxiang Medical University, Xinxiang, 435001 Henan China; 7 Key Laboratory of Animal Models and Human Disease Mechanisms of the Chinese Academy of Sciences and Yunnan Province, Kunming Institute of Zoology, Chinese Academy of Sciences, Kunming, 650223 China; 8 Institute of Psychiatry, Psychology and Neuroscience, King’s College London, London, WC2R 2LS UK; 9 State Key Laboratory of Cognitive Neuroscience and Learning, Beijing Normal University, Beijing, 100875 China; 10 Analytic and Translational Genetics Unit, Massachusetts General Hospital, Boston, MA 02114 USA; 11 Stanley Center for Psychiatric Research, The Broad Institute of MIT and Harvard, Cambridge, MA 02141 USA; 12 Department of Medicine, Harvard Medical School, Boston, MA 02115 USA; 13 Department of Biostatistics and Computational Biology, School of Life Sciences, Fudan University, Shanghai, 200438 China; 14 Department of Neurology, Xuanwu Hospital, Capital Medical University, Beijing, 100053 China; 15 State Key Laboratory of Natural and Biomimetic Drugs, Key Laboratory for Neuroscience for Ministry of Education, School of Pharmaceutical Sciences, Peking University Health Science Center, Beijing, 100191 China; 16 PKU-IDG/McGovern Institute for Brain Research, Peking University, Beijing, 100871 China; 17 Chinese Institute for Brain Research, Beijing, 102206 China

**Keywords:** Schizophrenia, Antipsychotic drug, Treatment response, Prediction model, Genetics, Epigenetics

## Abstract

**Background:**

Choosing the appropriate antipsychotic drug (APD) treatment for patients with schizophrenia (SCZ) can be challenging, as the treatment response to APD is highly variable and difficult to predict due to the lack of effective biomarkers. Previous studies have indicated the association between treatment response and genetic and epigenetic factors, but no effective biomarkers have been identified. Hence, further research is imperative to enhance precision medicine in SCZ treatment.

**Methods:**

Participants with SCZ were recruited from two randomized trials. The discovery cohort was recruited from the CAPOC trial (*n* = 2307) involved 6 weeks of treatment and equally randomized the participants to the Olanzapine, Risperidone, Quetiapine, Aripiprazole, Ziprasidone, and Haloperidol/Perphenazine (subsequently equally assigned to one or the other) groups. The external validation cohort was recruited from the CAPEC trial (*n* = 1379), which involved 8 weeks of treatment and equally randomized the participants to the Olanzapine, Risperidone, and Aripiprazole groups. Additionally, healthy controls (*n* = 275) from the local community were utilized as a genetic/epigenetic reference. The genetic and epigenetic (DNA methylation) risks of SCZ were assessed using the polygenic risk score (PRS) and polymethylation score, respectively. The study also examined the genetic-epigenetic interactions with treatment response through differential methylation analysis, methylation quantitative trait loci, colocalization, and promoter-anchored chromatin interaction. Machine learning was used to develop a prediction model for treatment response, which was evaluated for accuracy and clinical benefit using the area under curve (AUC) for classification, *R*^2^ for regression, and decision curve analysis.

**Results:**

Six risk genes for SCZ (*LINC01795*, *DDHD2*, *SBNO1*, *KCNG2*, *SEMA7A*, and *RUFY1*) involved in cortical morphology were identified as having a genetic-epigenetic interaction associated with treatment response. The developed and externally validated prediction model, which incorporated clinical information, PRS, genetic risk score (GRS), and proxy methylation level (proxyDNAm), demonstrated positive benefits for a wide range of patients receiving different APDs, regardless of sex [discovery cohort: AUC = 0.874 (95% CI 0.867–0.881), *R*^2^ = 0.478; external validation cohort: AUC = 0.851 (95% CI 0.841–0.861), *R*^2^ = 0.507].

**Conclusions:**

This study presents a promising precision medicine approach to evaluate treatment response, which has the potential to aid clinicians in making informed decisions about APD treatment for patients with SCZ.

*Trial registration* Chinese Clinical Trial Registry (https://www.chictr.org.cn/), 18. Aug 2009 retrospectively registered: CAPOC—ChiCTR-RNC-09000521 (https://www.chictr.org.cn/showproj.aspx?proj=9014), CAPEC—ChiCTR-RNC-09000522 (https://www.chictr.org.cn/showproj.aspx?proj=9013).

**Supplementary Information:**

The online version contains supplementary material available at 10.1186/s40779-023-00459-7.

## Background

Schizophrenia (SCZ), a complex mental disorder that affects 1% of people worldwide [1], leads to severe personal disability and imposes considerable burdens on public health and the economy [2]. Early and appropriate treatment with antipsychotic drug (APD) can control or improve symptoms and reduce the risk of relapse [3]. However, treatment response to APD, as measured by the reduction in scores on the Positive and Negative Syndrome Scales (PANSS), varies widely among individuals with SCZ and is hard to predict due to the lack of effective biomarkers. As a result, decision-making for medication treatment in SCZ can be a challenging and imprecise process, as it often involves a trial-and-error approach. This can lead to reduced adherence, thereby hindering the progress of precision medicine in psychiatry.

Studies have revealed the contribution of both genetic and epigenetic factors to the onset of SCZ [4, 5] and its response to treatment [6, 7]. Multiple therapeutic targets have been identified, such as muscarinic acetylcholine receptors [8], glutamate receptors (e.g., N-methyl-D-aspartate receptor), gamma-aminobutyric acid receptors, and oxytocin [9], leading to the recognition of SCZ subtypes with distinct neurobiological underpinnings [10]. Research on patients with treatment-resistant schizophrenia (TRS) also showed that genetic [11] and epigenetic [12] factors impact the outcome of antipsychotic drug treatment, suggesting subtype-specific responses in SCZ patients. However, limitations such as small sample sizes, potential bias from nonrandomized studies, a lack of comprehensive evaluation for different APD, and nonreproducible results impede the development of clinical biomarkers and personalized treatment for SCZ and require further attention. To date, no study has investigated the interaction between genetics and epigenetics or yielded a predictive biomarker for treatment response.

**Fig. 1:**
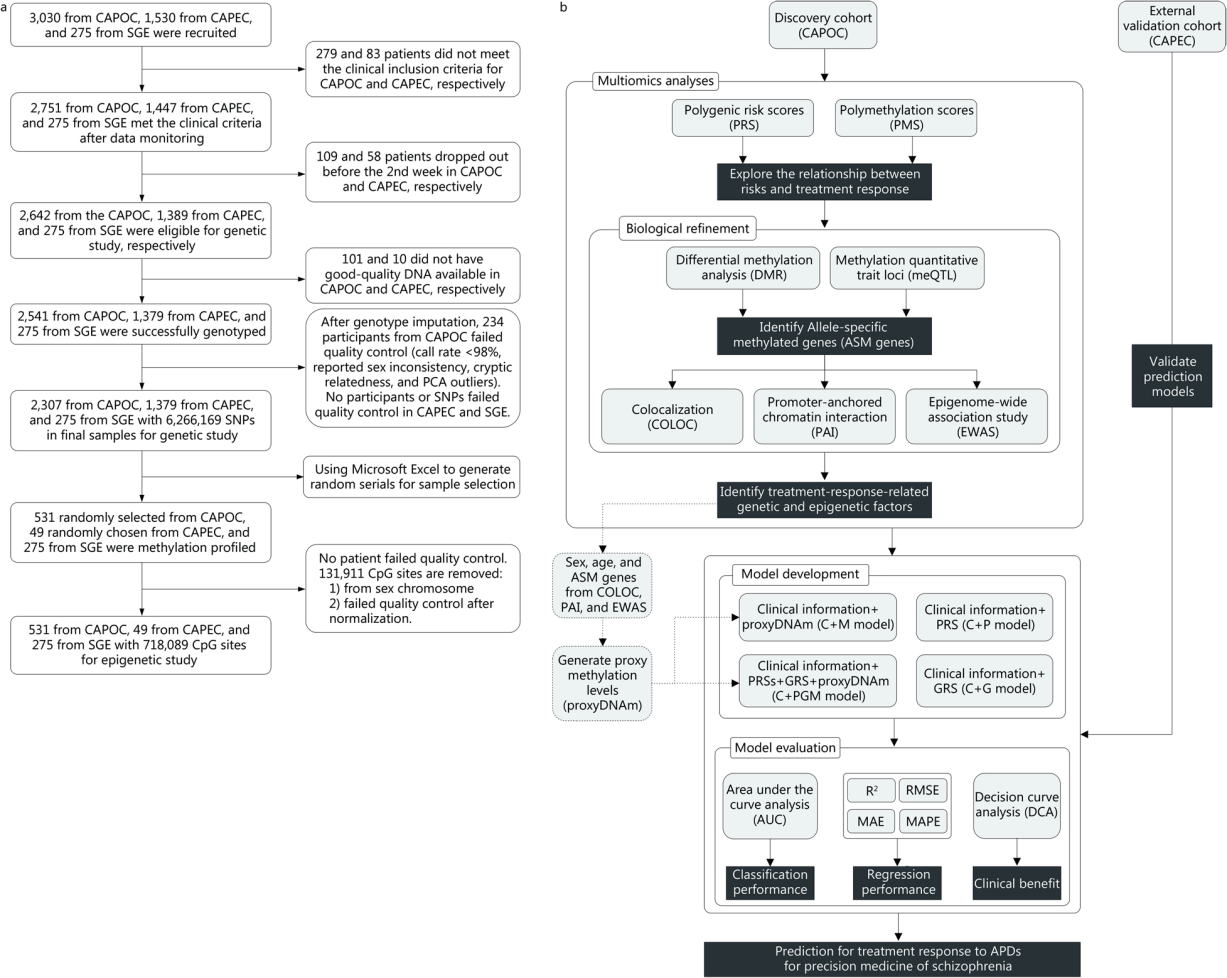
Research flow diagram. The flow diagrams illustrate the trial profiles and research design. **a** This study used participants from two randomized trials: CAPOC and CAPEC. The flow diagram describes the detailed profile of the trials. **b** Multiomics analyses were conducted to investigate the relationship between genetic/epigenetic risks of SCZ and treatment response to APDs, and developed a prediction model for treatment response in the discovery cohort (CAPOC). We used an external validation cohort (CAPEC) to validate the prediction models. *CAPOC* Chinese Antipsychotics Pharmacogenetics Consortium; *CAPEC* Chinese Antipsychotics Pharmacogenomics Consortium

To identify the genetic or epigenetic factors that determine a patient’s response to APD and develop practical predictive biomarkers for treatment response, we employed participants with SCZ from two large, multicenter, randomized trials as our discovery (CAPOC, *n* = 2307) [6] and external validation cohorts (CAPEC, *n* = 1379) [13]. As illustrated in Fig. 1, in the discovery cohort, we examined the correlations between treatment response to APD, genetic risk reflected by polygenic risk scores (PRS), and epigenetic risk reflected by polymethylation scores (PMS). A variety of techniques, including differential methylation analysis, methylation quantitative trait loci (meQTL), colocalization, promoter-anchored chromatin interaction (PAI), and epigenome-wide association study (EWAS), were utilized to identify specific factors associated with APD treatment response. We then developed and validated a prediction model for treatment response that incorporated clinical information, PRS, genetic risk score (GRS, a biomarker reported in our previous study [6]) and proxyDNAm. This model was robust, generalizable, and clinically useful, therefore helping to inform treatment decisions and support the use of a precision medicine approach for SCZ.

## Methods

### Study design and participants

This study followed the CONSORT [14] and TRIPOD [15] reporting guidelines for investigation and modeling. A total of 2307 patients with SCZ who received Olanzapine, Aripiprazole, Risperidone, Quetiapine, Haloperidol, Ziprasidone, or Perphenazine treatment for six weeks were recruited from the Chinese Antipsychotics Pharmacogenomics Consortium (CAPOC) across five research centers, including Peking University Sixth Hospital, West China Hospital of Sichuan University, the Second Xiangya Hospital of Central South University, Beijing Anding Hospital Affiliated to Capital Medical University, and Beijing Huilongguan Hospital [6]. A total of 1379 patients with SCZ who received Aripiprazole, Olanzapine, or Risperidone treatment for 8 weeks were recruited from the Chinese Antipsychotics Pharmacogenetics Consortium (CAPEC) across multiple hospitals, including Peking University Sixth Hospital, Beijing Huilongguan Hospital, the Sixth Hospital of Hebei Province, Jinzhou Kangning Hospital, and Xi’an Mental Health Centre) [13]. To calculate the PRS and conduct the risk-related methylation analysis, 275 healthy controls (HCs) were employed and were genotyped and methylation profiled under the same pipeline. HCs were recruited from our Schizophrenia × Gene × Environment project (SGE) [16] and were recruited from the local community through advertisement under the screening of Structured Clinical Interview for Diagnostic and Statistical Manual of Mental Disorders IV (DSM-IV, nonpatient edition). HCs had no lifetime history of psychotic illness and no family history of psychosis. All participants were of Han Chinese ancestry and were right-handed. The age of HCs was (24.7 ± 3.2) years old, and the ratio of males to females was 138:137. To ensure that sample sizes met the statistical requirements for subsequent analyses, we calculated the statistical power of sample size by G*power software (version 3.1, https://www.psychologie.hhu.de/arbeitsgruppen/allgemeine-psychologie-und-arbeitspsychologie/gpower) under the models of Pearson correlation, *χ*^2^ correlation, analysis of variance (ANOVA), and multiple linear regression (see Additional file 2: Fig. S1). All study protocols were approved by the Institutional Ethics Review Boards at each site and can be accessed in the Chinese Clinical Trial Registry (https://www.chictr.org.cn/showproj.aspx?proj=9013 and https://www.chictr.org.cn/showproj.aspx?proj=9014). Written informed consent was obtained from all participants.

### Inclusion and exclusion criteria

*Inclusion criteria* (1) had a diagnosis of SCZ based on the Structured Clinical Interview of DSM-IV; (2) were of Han Chinese ancestry; (3) were aged 18–45 years; (4) scored more than 60 on the PANSS and scored more than four on at least three positive items; (5) were physically healthy with all laboratory parameters within normal limits; (6) were able to provide informed consent. Both first-episode and relapsed patients with SCZ were enrolled from the inpatient departments of the psychiatric hospitals affiliated with CAPOC or CAPEC.

*Exclusion criteria* (1) were diagnosed with schizoaffective disorder, delusional disorder, brief psychotic disorder, schizophreniform disorder, psychosis associated with substance use or medical conditions, learning disability, pervasive developmental disorder, delirium, dementia, amnesia, or other cognitive disorders; (2) had severe, unstable physical diseases (such as diabetes, thyroid diseases, hypertension, and cardiac diseases), malignant syndrome or acute dystonia, well documented histories of epilepsy and hyperpyretic convulsion, a DSM-IV diagnosis of alcohol or drug dependence, or a history of drug-induced neuroleptic malignant syndrome; (3) required long-acting injectable medication to maintain treatment adherence; (4) were regularly treated with clozapine for treatment resistance [17] during the past month (patients who had taken clozapine for reasons other than treatment resistance were eligible); (5) were treated with electroconvulsive therapy during the last month; (6) had previously attempted suicide, or had experienced the symptoms of severe excitement and agitation; (7) had abnormal liver or renal function (i.e., aspartate aminotransferase ≥ 80 U/L, alanine aminotransferase ≥ 80 U/L, blood urea nitrogen ≥ 9.75 mmol/L, urine creatinine ≥ 21.6 mmol/d); (8) did not have a legal guardian (it was a hospital stipulation that written informed consent was required from the patient's legal guardian); (9) had QTc prolongation, a history of congenital QTc prolongation, or recent (i.e., within the past 6 months) myocardial infarction; (10) were pregnant or breastfeeding; or 11) had a contraindication to any of the drugs to which they could be assigned (only applicable to patients).

### Randomization

As described in our previous study [6], we used a Microsoft Excel randomization generator without any stratification factors to establish the group assignment. A trained research assistant who had no further role in the trial generated the random allocation sequence, which would be concealed until after baseline assessments. The researchers performing both the baseline and the follow-up assessments were masked to the group assignments of each participant. Patients and psychiatrists were unmasked to assigned APD: in the CAPOC cohort, we randomly and equally allocated consecutive eligible patients to the Aripiprazole, Olanzapine, Quetiapine, Risperidone, Ziprasidone, or one of the first-generation APD (Haloperidol or Perphenazine) groups; those randomly assigned to the first-generation APD group were subsequently randomly and equally assigned to receive Haloperidol or Perphenazine. In the CAPEC cohort, we randomly and equally assigned the eligible patients to the Olanzapine, Risperidone, or Aripiprazole group for 8-week treatment. Using the same randomization generator, we randomly selected participants for methylation profiling from both the CAPOC and CAPEC cohorts.

### Treatment procedure

According to the study protocol [6], the dosages of APD were appropriately adjusted within 2 weeks of randomization based on the effectiveness of the treatment. Each APD had a permissible range of dosages, with olanzapine ranging from 5 to 20 mg/d, risperidone ranging from 2 to 6 mg/d, quetiapine ranging from 400 to 750 mg/d, aripiprazole ranging from 10 to 30 mg/d, ziprasidone ranging from 80 to 160 mg/d, haloperidol ranging from 6 to 20 mg/d, and perphenazine ranging from 20 to 60 mg/d. Subsequently, the dosages were maintained at a constant level throughout the duration of the study. The equivalent dose for each APD used in this study was calculated according to the chlorpromazine equivalent dose part in Additional file 1.

### Primary outcome and subgrouping rule for multiomics analysis

The primary outcome as treatment response was evaluated by the PANSS reduction rate at the last follow-up, which can be calculated as described below:

$$PANSS\,reduction\,rate = \frac{{PANSS\,baseline\,total\,score - PANSS\,endpoint\,total\,score}}{{PANSS\,baseline\,total\,score - 30}} \times 100$$

As the treatment response is a continuous variable, we labeled participants with SCZ into two groups: response group (RES, PANSS reduction rate ≥ 50%) and nonresponse group (non-RES, PANSS reduction rate < 50%).

### Estimation of genetic and epigenetic risks

Polygenic risk scores (PRSs) were utilized to estimate the genetic risk. The calculation of PRS in the case–control study was conducted using the software PRSice2 (version v2.3.3, https://choishingwan.github.io/PRSice). The parameters for calculation included: (1) employing binary phenotype mode (case and control); (2) using summary level genome-wide association study data for SCZ, bipolar disorder (BP), and major depressive disorder (MDD) in the East Asian population from the *Psychiatric Genomics Consortium* (www.med.unc.edu/pgc) as the genetic reference; (3) determining empirical *P*-value through 10,000 permutations; (4) incorporating principal components that explained the 95% variances, batch number and genotyping platform as covariates.

The epigenetic risk was estimated using the epigenetic clocks and PMS. Epigenetic clocks were calculated by DNA Methylation Age Calculator (https://dnamage.genetics.ucla.edu), which normalized the data used in the calculation, as opposed to normalization by our methylation profiling pipeline. DNA methylation age and DNA methylation age acceleration, two recommended epigenetic clock measurements [18], were selected to estimate the epigenetic clock profile. The PMS was calculated in two steps by the R package BioMM [5]. The first step involved constructing machine learning models for each pathway, where CpG sites were mapped. The second step entailed constructing a collective model of the pathway models. The final output was a quantified score representing the likelihood of participants having SCZ. For each step, 1000 bootstrapping iterations were conducted.

### Multiomics analyses

Genotyping and DNA methylation data were obtained from peripheral whole blood samples of participants. Through high-throughput sequencing procedures and quality control (see Genotyping and methylation quantification in Additional file 1), genotyping data for 6,266,169 SNPs from 3961 participants (2307 participants in CAPOC, 1379 participants in CAPEC, and 275 participants in SGE) were acquired. Among these participants with genotyping data, DNA methylation detection was conducted on 855 participants (531 participants randomly selected from CAPOC, 49 participants randomly chosen from CAPEC, and 275 participants in SGE), and methylation data for 718,089 CpG sites were obtained. For participants with methylation data, technical replication (see Technical replication of DNA methylation profiling in Additional file 1) was conducted: 194 participants were randomly chosen for Illumina sequencing-based BSP detection to verify the chip detection site results, and among these 194 participants, 20 were further randomly selected to undergo Sequenom MassARRAY® Methylation validation.

Differential methylation analysis was conducted to identify risk-related differentially methylated regions (risk-DMRs) between cases (patients with SCZ) and controls (HCs) as well as response-related DMRs (RES-DMRs) between the RES and non-RES groups. meQTL analysis was used to find genetic-epigenetic interactions and locate allele-specific methylated (ASM) genes from risk-DMRs and RES-DMRs, which were validated in the mQTL Database (http://www.mqtldb.org). Biological refinement was performed for ASM genes: (1) Bayesian colocalization analysis was used to find a locus affecting traits of treatment response and risk of SCZ; (2) PAI analysis was used to estimate chromatin accessibility and to identify the RES-related altered PAI genes from ASM genes; (3) EWAS was used to determine which CpG site’s (from ASM genes) methylation level was associated with treatment response. A detailed protocol for each analysis can be found in the Additional file 1 section, including methylation quantitative trait loci (meQTL) analysis, Bayesian colocalization analysis, epigenome-wide association analysis, epigenome-wide differential methylation analysis, and prediction of promoter-anchored chromatin interaction.

### Machine-learning model development

A machine-learning approach was utilized to regress the methylation level (output) from the genotype of meQTL (input, with covariates including age and sex) as a proxy DNA methylation (proxyDNAm) model by the R package caret (https://github.com/topepo/caret). The epigenome-wide methylation-profiled samples from the discovery cohort and SGE cohort were divided into the training dataset (a total of 602 participants) and the test dataset (a total of 258 participants) at a 7:3 ratio. Due to the sensitivity of machine-learning algorithms to data distribution, data in the training dataset were cantered, scaled, and Gaussian-distributed mapped before model development by using the “preProcessing” function from the R package caret. The preprocessing pattern (mean and standard error) from the training dataset was stored and applied to the test dataset to prevent data leakage. Quantile random forest (QRF), random forest (RF), and support vector machines with polynomial kernel (SVMPoly) were utilized to build the proxy models. To mitigate the underfitted or overfitted issues, a 10-time repeated tenfold cross-validation (10 × tenfold CV) or leave-one-out cross-validation (LOOCV) was performed in the training stage. Hyperparameters [n_estimators (the number of trees in the forest), max_depth (the maximum depth of the trees), min_samples_split (the minimum number of samples required to split an internal node), min_samples_leaf (the minimum number of samples required to be at a leaf node), and max_features (the number of features to consider when looking for the best split) for QRF and RF; C (the regularization parameter), degree (the degree of the polynomial kernel), gamma (the kernel coefficient), coef0 (the independent term in the polynomial kernel function), shrinking (whether to use the shrinking heuristic), tol (the tolerance for stopping criterion), and max_iter (the maximum number of iterations) for SVMPoly] for each algorithm were optimized by the random search function. The proxy models were built for 28 CpG sites that have a significant correlation of methylation level between brain and blood. The performance of the proxy models was assessed by determining the Pearson correlation coefficient between raw and predicted values. Sixteen proxy models with correlation significance (Pearson’s *P *value ≤ 0.05) in the test dataset were deemed suitable for generating the proxy methylation level.

We used the clinical information (PANSS baseline score, sex, age, and APD), PRS, GRS, and proxy methylation level (proxyDNAm) in four combination patterns (clinical information + PRS, clinical information + GRS, clinical information + proxyDNAm, and clinical information + PRS + GRS + proxyDNAm) to develop the prediction model for treatment response (RES-prediction model) with the algorithms including quantile random forest, random forest, and support vector machines with radial basis function kernel by R package caret. The preprocessing and optimization of the hyperparameters of the models were the same as those of proxyDNAm. We performed 10 times tenfold cross-validation or leave-one-out cross-validation (LOOCV) to train the model to avoid underfitting or overfitting issues. The performance of RES-prediction models in classification was evaluated by the area under the curve (AUC), while their performance in regression was evaluated by metrics including the mean absolute error (MAE), root mean square error (RMSE), mean absolute percentage error (MAPE), coefficient of determination (*R*^2^), and correlation coefficient by R package MLmetrics (https://github.com/yanyachen/MLmetrics). Clinical values of RES-prediction models were evaluated by decision curve analysis in the R package rmda (https://github.com/mdbrown/rmda).

### Statistical analysis

All statistical analyses were conducted through R package stats (version 4.2.2). Pearson correlation analysis was employed to estimate the correlation coefficient between two continuous variables. The Wilcoxon test was utilized to assess the difference between the means of continuous variables of two groups, considering a *P *value < 0.05 as significant.

## Results

### Characteristics of participants and study design

The demographic and clinical characteristics and the study design are described in Table 1 and Fig. 1, respectively. The statistical power of the given sample size was sufficient and is described in Additional file 2: Fig. S1. Technical replication of DNA methylation profiling indicated the nominal effect of APD on methylation and a trusted profiling result (Additional file 2: Fig. S2 and Table S1). The chlorpromazine equivalent doses of participants with SCZ are represented in Additional file 2: Table S2 and revealed that the final doses of ziprasidone, aripiprazole, and perphenazine were significantly higher in the non-RES group than in the RES group. Partial correlation analysis, controlling for drugs, sex, and age, revealed that chlorpromazine equivalent doses were not significantly correlated with the PANSS total score at baseline or the PANSS reduction rate.

**Table 1:** Demographic and clinical characteristics of participants recruited in analyses from CAPOC, CAPEC, and SGE (as healthy control, HC)

Item	CAPOC (*n* = 2307)							CAPEC (*n* = 1379)			HC (*n* = 275)
	Olanzapine (*n* = 398)	Aripiprazole (*n* = 386)	Risperidone (*n* = 394)	Quetiapine (*n* = 375)	Haloperidol (*n* = 175)	Ziprasidone (*n* = 386)	Perphenazine (*n* = 193)	Olanzapine (*n* = 459)	Aripiprazole (*n* = 462)	Risperidone (*n* = 458)	/
Male [*n* (%)]	200(50.3)	185(47.9)	228(57.9)	176(46.9)	85(48.6)	193(50.0)	89(46.1)	245(53.4)	235(50.9)	232(50.7)	137(50.1)
Age (years, mean ± SD)	30.2 ± 7.9	31.1 ± 7.8	30.6 ± 7.9	30.8 ± 7.7	32.7 ± 7.6	30.3 ± 7.9	31.8 ± 8.0	31.6 ± 6.3	32.8 ± 11.3	32.4 ± 10.4	24.7 ± 3.2
Baseline PANSS total score (mean ± SD)	88.6 ± 15.3	89.3 ± 15.0	89.3 ± 14.8	91.0 ± 15.1	90.9 ± 16.0	91.0 ± 15.4	92.33 ± 15.7	104.5 ± 22.5	94.1 ± 18.1	86.0 ± 13.5	–
Endpoint PANSS total score (mean ± SD)	54.2 ± 14.7	59.0 ± 19.0	54.4 ± 14.2	59.1 ± 17.1	57.0 ± 15.7	61.1 ± 18.0	60.5 ± 18.7	69.8 ± 22.8	67.3 ± 18.1	59.9 ± 16.9	–
PANSS reduction rate (mean ± SD)	55.9 ± 24.7	47.8 ± 29.1	55.3 ± 25.0	48.2 ± 28.2	53.4 ± 24.2	46.6 ± 28.2	49.2 ± 27.8	48.0 ± 22.9	41.6 ± 22.5	47.1 ± 26.0	–
RES [*n* (%)]	253(63.6)	189(49.0)	252(64.0)	191(50.9)	102(58.3)	181(46.9)	103(53.4)	268(58.4)	278(60.2)	280(61.1)	–
Follow-up (week)	6							8			–

*PANSS* positive and negative syndrome scale; *CAPOC* Chinese Antipsychotics Pharmacogenomics Consortium; *CAPEC* Chinese Antipsychotics Pharmacogenetics Consortium; *HC* healthy control; *RES* response

The endash symbol “–” means the item has no treatment or is not applicable for PANSS total score at baseline and endpoint, PANSS reduction rate, RES percentage, or follow-up duration

### Genetic and epigenetic risks of SCZ reflected treatment response

Given the shared symptoms of anhedonia, abnormal social behavior, and impaired brain connectivity and the demonstrated genetic coheritability among SCZ, BP, and MDD [19], we calculated the PRSs of SCZ (PRS-SCZ) [4], BP (PRS-BP) [20], and MDD (PRS-MDD) [21] based on three large GWAS (genome-wide association study) studies in the East Asian population to investigate the relationship between treatment response and genetic risks of SCZ, BP, and MDD. A significant correlation between PRS-SCZ and PRS-BP was identified (*r* = 0.106, 95% CI 0.066–0.146, *P* = 2.73 × 10^–7^), but not between the PRS-SCZ and PRS-MDD (*r* = 0.027, 95% CI − 0.013 to 0.067, *P* = 0.190; Additional file 2: Fig. S3). Furthermore, a significant correlation was observed between treatment response and PRS-SCZ (*r* = − 0.045, 95% CI − 0.085 to − 0.003, *P* = 0.032; Additional file 2: Fig. S4). While PRS-BP and PRS-MDD did not display significant correlations with treatment response, their contribution to explaining the variance in treatment response was noted (Additional file 2: Fig. S5).

Subsequently, the relationship between treatment response and epigenetic risks for SCZ, as estimated by two epigenomic assessments (epigenetic clock [18] and genome-wide PMS [5]), was investigated in methylation-profiled participants (*n*_case_ = 531, *n*_control_ = 280). A significant correlation between treatment response and PMS was found (*r* = − 0.150, *P* = 3.7 × 10^–4^; Additional file 2: Fig. S6), whereas no significant correlation with the epigenetic clock was observed (Additional file 2: Fig. S7).

### Identify treatment response-related genetic and/or epigenetic factors

Differential methylation (differentially methylated region, DMR) analysis and meQTL analysis were conducted to detect DNA methylation changes and examine the genetic-epigenetic interactions. A total of 9707 DMRs (risk-DMRs, including 107,095 CpG sites mapped to 10,666 genes; top 20 risk-DMRs are listed in Additional file 2: Table S3) were identified between cases and controls (*n*_case_ = 531, *n*_control_ = 280), as well as 266 DMRs (RES-DMRs, including 6474 CpG sites mapped to 421 genes; top 20 RES-DMRs are listed in Additional file 2: Table S4) between non-RES and RES (*n*_non-RES_ = 196, *n*_RES_ = 384). Furthermore, 378,825 single-nucleotide polymorphism (SNP)-CpG pairs were identified as meQTLs, with 168,978 SNPs affecting 55,712 CpG sites (*P*_adj_ < 1 × 10^–8^). The CpG sites from DMRs and meQTLs were mainly enriched in the region of promoters (e.g., 1st exon, 5'UTR, TSS200 and TSS1500) within the CpG island, suggesting the regulation of allele-specific methylation on the gene expression of transcription factors (Additional file 2: Fig. S8).

**Fig. 2:**
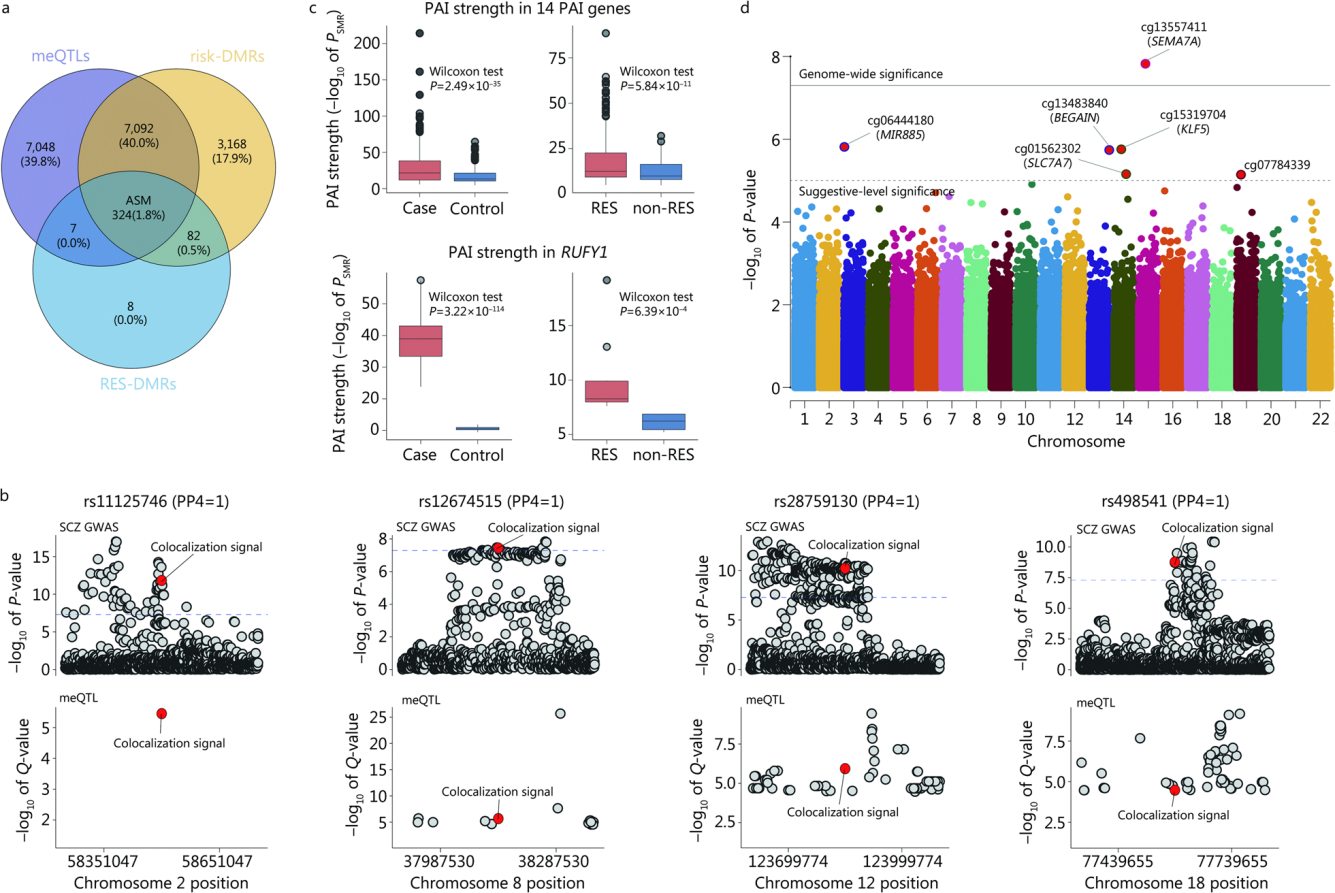
Multiomics analyses. Genetic and epigenetic factors reflecting treatment response were investigated by meQTL and DMR, and the results were refined using three different approaches. **a** Upon comparison of meQTLs, risk-DMRs, and RES-DMRs, 324 genes were identified as ASM genes associated with SCZ risk and treatment response. **b** Within the ASM genes, colocalization identified four signals, including rs11125746 in LINC01795, rs12674515 in DDHD2, rs28759130 in SBNO1, and rs498541 in KCNG2. **c** PAI identified 14 genes showing significant differences in overall PAI strength under the case‒control condition and non-RES versus RES condition. Among them, RUFY1 displayed brain‒blood consistency in methylation, transcription, and chromatin interaction, along with a significant difference in PAI strength. **d** EWAS identified one genome-wide significant (*P*_adj_ < 1 × 10^–8^) signal located in the *SEMA7A* gene. Solid and dashed gray lines represent genome-wide and suggestive significance, respectively. meQTL. Methylation quantitative trait loci; DMR. Differentially methylated region; PAI. Promoter-anchored chromatin interaction; SCZ. Schizophrenia; RES. Response; ASM. Allele-specific methylated; GWAS. Genome-wide association study; PP4. Posterior probability for a shared signal; SMR. Summary-based Mendelian randomization; LINC01795. Long intergenic non-protein coding RNA 1795; DDHD2. DDHD domain containing 2; SBNO1. Strawberry notch homolog 1; KCNG2. Potassium voltage-gated channel modifier subfamily G member 2; RUFY1. RUN and FYVE domain containing 1; MIR885. microRNA 885, BEGAIN brain enriched guanylate kinase associated; SLC7A7. Solute carrier family 7 member 7; KLF5. KLF transcription factor 5; SEMA7A. Semaphorin 7A

A total of 324 SCZ risk- and treatment response-related genes were discovered to exhibit ASM (ASM genes, Fig. 2a). These genes demonstrated high expression levels in brain regions such as the cerebral cortex, hippocampus, and cerebellum and were involved in metabolic processes, protein binding, and intracellular anatomical structures. Additionally, they were associated with abnormal brain morphologies and neurological or psychiatric diseases, such as Alzheimer's disease, Parkinson’s disease, intellectual disability, and cocaine addiction (Additional file 2: Fig. S9 and Table S5).

Colocalization analysis identified 1047, 1042, and 1 meQTL colocalized with SCZ risk loci from the summary GWAS results of SCZ, BP, and MDD, respectively (PP4 > 0.8, PP4: posterior probability for a shared signal). Four colocalization signals were identified in the ASM genes (called COLOC genes, Fig. 2b), including rs11125746 (chr2:58501047 G > A, *LINC01795*), rs12674515 (chr8:38137530 A > G, *DDHD2*), rs28759130 (chr12:123849774 C > A, *SBNO1*) and rs498541 (chr18:77589655 G > A, *KCNG2*).

PAI analysis [22] revealed a significant difference in chromatin interaction strength (− log_10_ of PAI) in 14 ASM genes (*P*_SMR_ < 5% FDR and *P*_HEIDI_ > 0.01 (SMR: Summary-based Mendelian Randomization;HEIDI: Heterogeneity in dependent instruments), called PAI genes, Fig. 2c) between cases and controls (Wilcoxon test, two-tailed, *P* = 2.49 × 10^–35^) and between non-RES and RES groups (Wilcoxon test, two-tailed, *P* = 5.84 × 10^–11^). Data investigation of sequenced methylation, transcription, and chromatin interaction in multiple tissues found that *RUFY1* (Chr5: 179550554—179610012), which is a gene associated with endolysosomal recycling [23], cortical surface area, and cortical thickness [24], showed blood‒brain consistency in methylation, transcription, and chromatin interaction (Additional file 2: Fig. S10).

EWAS detected one genome-wide significant (*P*_adj_ < 1 × 10^–8^) signal located in *SEMA7A* (called the EWAS gene, Fig. 2d) from the ASM genes.

Linkage disequilibrium analysis suggested that the genes from COLOC, PAI, and EWAS were associated with cortical morphology (Additional file 2: Table S6–S11). The genetic-epigenetic interactions from the meQTLs of the genes identified in COLOC, PAI, and EWAS were validated by the mQTL database, with the exception of rs11125746 from *LINC01795*.

### Development, validation, and evaluation of the predictive model for treatment response

According to allele-specific methylation in genetic-epigenetic interactions, information on age, sex and meQTLs from *LINC01795*, *DDHD2*, *SBNO1*, *KCNG2*, *SEMA7A*, and *RUFY1* was included to generate proxyDNAm models (*n*_train_ = 568, *n*_test_ = 243) for the CpG sites that were affected by the meQTLs and showed high brain-blood correlation in DNA methylation (Additional file 2: Table S12). Finally, proxyDNAm models for 18 CpG sites from five meQTL-validated genes (*DDHD2*, *SBNO1*, *KCNG2*, *SEMA7A*, and *RUFY1*) were established (Additional file 2: Fig. S11 and Table S13).

**Fig. 3:**
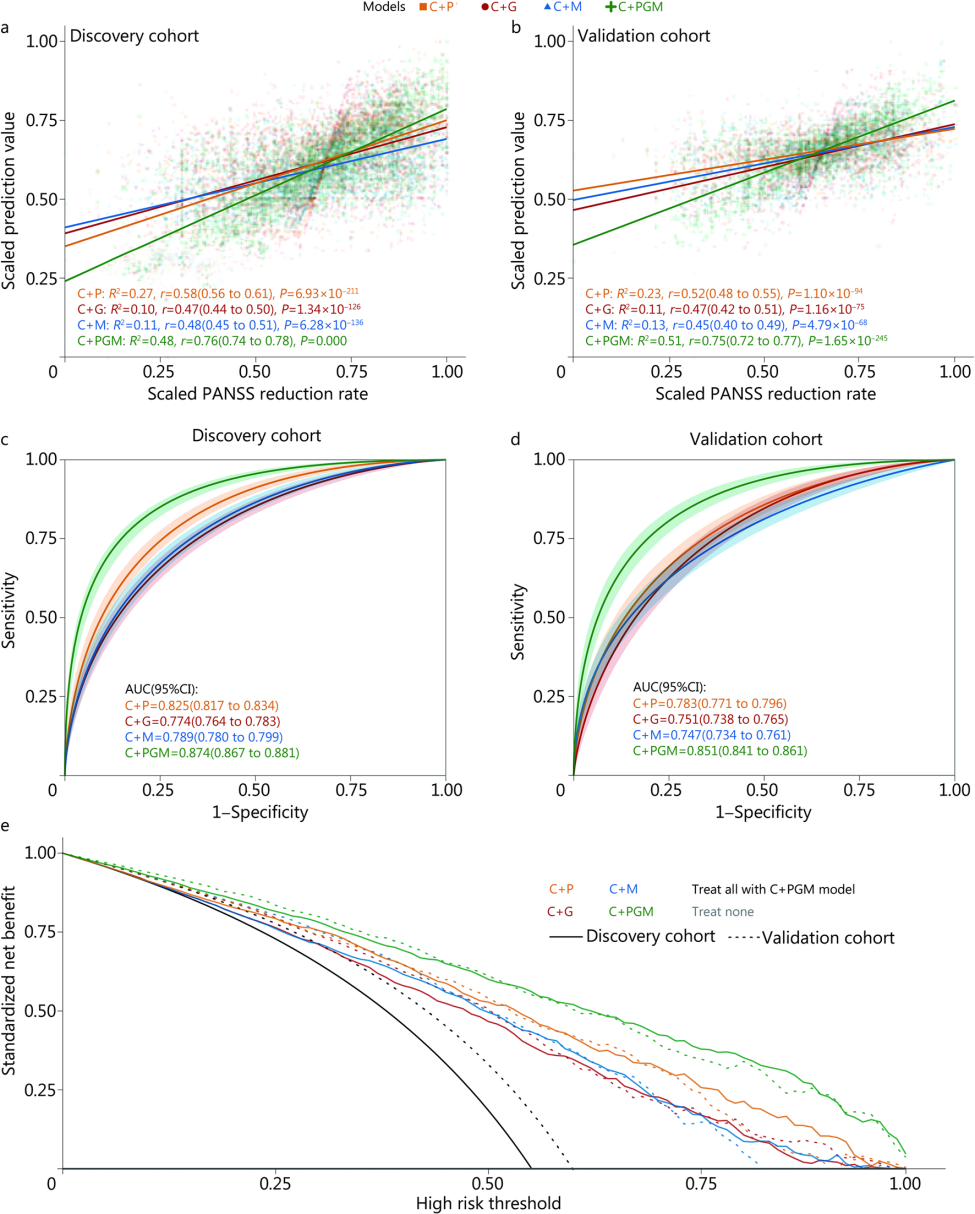
Performance of the optimal prediction model for treatment response in the discovery and validation cohorts. Visualization of regression performance of four RES-prediction models in **a** discovery cohort and **b** external validation cohort. The solid line represents the linear relationship between the scaled prediction value and scaled PANSS reduction rate. **c, d** illustrate the AUCs of four RES-prediction models. Solid lines in different colors represent receiver operating curves for different RES-prediction models, and the ribbons in different colors represent the confidence intervals of different models in the **c** discovery cohort and **d** external validation cohort. **e** Decision curves for four RES-prediction models in the discovery cohort (solid line) and external validation cohort (dashed line); lines in different colors represent different models. PANSS. Positive and negative syndrome scale; RES. Response; AUC. Area under the curve; C + P. Clinical information + PRS; C + G. Clinical information + GRS; C + M. Clinical information + proxyDNAm; C + PGM. Clinical information + PRS + GRS + proxyDNAm; PRS. Polygenic risk score; GRS. Genetic risk score

Our primary objective was to develop a prediction model for treatment response. To accomplish this, we used clinical information (PANSS baseline score, APD, sex, and age), proxyDNAm, PRSs, and GRS to assess which combination pattern of data was adequate to develop the RES-prediction model [clinical information + PRSs (C + P model), clinical information + GRS (C + G model), clinical information + proxyDNAm (C + M model), and clinical information + PRSs + GRS + proxyDNAm (C + PGM model)]. Figure 3a, b illustrate the regression performance of four RES-prediction models in the discovery cohort (*n* = 2307) and external validation cohort (*n* = 1379), respectively. Figure 3c and d illustrate the classification performance of four RES-prediction models in the discovery cohort and external validation cohort, respectively. The C + PGM model [discovery cohort: AUC = 0.874 (95% CI 0.867–0.881), *R*^2^ = 0.478, *r* = 0.76 (95% CI 0.74–0.78); external validation cohort: AUC = 0.851 (95% CI 0.841–0.861), *R*^2^ = 0.507, *r* = 0.75 (95% CI 0.72–0.77)] outperformed other models (Additional file 2: Table S14) in predicting treatment response. The performance of the C + PGM model in different APD and in males and females is described in Table 2.

**Table 2:** Classification and regression performance of the optimal prediction model (C + PGM model) for treatment response

Cohort		AUC	MAE^*^	MAPE^*^	RMSE^*^	*R*^2^
*Discovery cohort (CAPOC, n = 2307)*						
APD	All	0.874	0.209	1.332	0.272	0.478
	Aripiprazole	0.882	0.220	1.203	0.290	0.481
	Haloperidol	0.850	0.179	2.243	0.236	0.501
	Olanzapine	0.878	0.180	1.644	0.239	0.510
	Perphenazine	0.901	0.211	1.185	0.267	0.517
	Quetiapine	0.856	0.241	1.081	0.305	0.392
	Risperidone	0.892	0.181	1.089	0.239	0.521
	Ziprasidone	0.865	0.235	1.291	0.299	0.414
Sex	Male	0.875	0.196	1.316	0.256	0.536
	Female	0.880	0.221	1.347	0.287	0.418
*Validation cohort (CAPEC, n = 1379)*						
APD	All	0.851	0.154	1.470	0.205	0.507
	Aripiprazole	0.882	0.151	1.114	0.195	0.564
	Olanzapine	0.840	0.155	1.628	0.208	0.468
	Risperidone	0.831	0.155	1.671	0.212	0.484
Sex	Male	0.843	0.151	1.926	0.205	0.500
	Female	0.860	0.156	1.043	0.205	0.513

*AUC* area under the curve; *MAE* mean absolute error; *MAPE* mean absolute percentage error; *RMSE* root mean squared error; *R*^2^ coefficient of determination; *APD* antipsychotic drug; *CAPOC* Chinese Antipsychotics Pharmacogenomics Consortium; *CAPEC* Chinese Antipsychotics Pharmacogenetics Consortium;

^*^Predicted value and record positive and negative syndrome scales (PANSS) reduction rate were scaled into the range from − 1 to 1

Decision curve analysis revealed that the C + PGM model offered the highest standardized net benefit at all risk thresholds (Fig. 3d, solid line and dashed line in green) compared to other RES-prediction models in both the discovery cohort and external validation cohort, suggesting its potential as a precision medicine approach.

## Discussion

In this study, we found the correlation between genetic/epigenetic risks of SCZ, BP, and MDD and treatment response to APDs, in which we located the genetic-epigenetic interactions from six genes involved in cortical morphology. Based on the observation, we developed and externally validated a prediction model to estimate the treatment response of patients with SCZ when receiving different APDs, which incorporated the clinical information (age, sex, and APD), genetic risks of SCZ, BP, and MDD, proxyDNAm, and GRS [6]. The prediction model in external validation showed good regression performance as well as clinical net benefit across all thresholds of risks, suggesting that it can inform clinicians of the estimated treatment response to APDs, thereby aiding in the choice of APDs and improving the treatment outcomes of patients with SCZ.

The high biological interpretability of the results was the first advantage of our study. Abnormalities in cortical morphology (e.g., thinner cortex and smaller cortical surface area) were unique to SCZ [25] and distinguished it from BP and MDD [26]. Previous studies have identified overlapped risk loci between SCZ, cortical thickness, and cortical surface area [27, 28] and established connections between treatment response and cortical morphology [29]. Our study identified six genes (*LINC01795*, *DDHD2*, *SBNO1*, *KCNG2, RUFY1,* and *SEMA7A*) that are associated with cortical morphology and play crucial roles in neurofunction. For example, *DDHD2* encodes a phospholipase enzyme involved in endosomal membrane trafficking [30], while *KCNG2* encodes a potassium voltage-gated channel-related protein[31]. *SEMA7A* regulates axon guidance, synapse elimination, hippocampal neurogenesis, mesolimbic dopaminergic pathways, and maturation of the cortical circuit [32, 33]. *RUFY1* encodes an effector protein for small GTPases, influences receptor surface expression and modulates dopamine release, synaptic current, glutamatergic transmission, membrane excitability, and long-term depression [34–39]. The CpG sites identified in our study may influence gene transcriptional expression, as methylation levels in promoter regions can inhibit transcription, while gene body methylation can enhance transcription [40]. Additionally, individual DNA methylation levels are stable in the long-term and are less affected by antipsychotic drug treatment [41–43], thus enabling us to reflect DNA methylation changes in the brain through peripheral blood samples, which involve a low economic burden for patients and are widely accessible. Therefore, the model can inform the selection of APDs before treatment and the adjustment of APDs during treatment.

The second strength of our study was the flexibility and robustness of our prediction model. The proxy methylation models (proxyDNAm) were developed to infer DNA methylation levels from meQTL, serving as a middleware that leverages genotype information to provide epigenetic information for the RES-prediction model. The RES-prediction model is flexible in terms of input and can save costs compared to methylation profiling, and it can also cooperate with other genetic biomarkers for improved prediction accuracy. The RES-prediction model was externally validated and demonstrated net benefit in predicting treatment response for all risk thresholds, offering accurate predictions for classification (respond/not respond) and regression (response quality) to guide APDs choices and improve adherence. The RES-prediction model, which was externally validated and showed good performance in classification (AUC = 0.851, 95% CI 0.841–0.861) and regression (*R*^2^ = 0.507) as well as the net clinical benefit, performed equally well in different treatment options and sexes, demonstrating its potential clinical utility for evaluating treatment response and guiding treatment choices as a promising precision medicine implementation. In comparison with other studies, a search of PubMed for articles on “treatment response”, “antipsychotic drugs”, and “schizophrenia” between 2012 and 2022 found 346 articles, including 42 clinical trials, and only one study established an externally validated prediction model (*n* = 21, *R*^2^ = 0.515) [44].

The third benefit of our study is its larger sample size in comparison to other studies. Our study, with a sample size of 3,686 patients with SCZ and robust prediction model through external validation, is the largest multiomics study of treatment response to date (the second and the third largest studies had 2586 [45] and 1100 patients [46], respectively). The sample sizes of the trials we reviewed ranged from 21 to 764, with a median of 117. Larger sample sizes are important in clinical research, as they increase statistical power and improve the generalizability of results.

The fourth advantage of our investigation is the examination of various types of APDs. Our review of published research revealed that a majority of the studies investigated the efficacy of single APD treatment using olanzapine, risperidone, or aripiprazole. This narrow focus, however, restricts the generalizability of the findings from such studies.

Studies have indicated that SCZ is influenced by a combination of genetics and environmental factors such as life events and maternal exposures [47]. This highlights the role of both genetic and epigenetic factors, as well as their interaction in the pathogenesis of SCZ [48, 49]. Previous studies have also reported a genetic overlap between SCZ pathogenesis and the mechanism of action of APDs [50, 51], which supported our observation. Changes in DNA methylation have been linked to treatment response in SCZ, particularly in cases of TRS [12, 52]. However, to date, no study has explored the relationship between interaction of genetics and epigenetics and treatment response. The genes identified in our study have also been linked to other mental health disorders, including Alzheimer’s disease [23] and opioid dependence [31]. This suggests that therapeutic targets for these disorders may have potential for use in the treatment of SCZ.

## Limitations

The current study has several limitations that need to be addressed in future research. First, the time frame for measuring treatment response was restricted, and the study was conducted in a controlled setting. Second, the study did not include an investigation of patients with TRS. Last, the study only investigated a limited selection of machine learning algorithms.

To address these limitations, future research needs to include a broader range of clinical measurements and settings, a comprehensive evaluation of genetic and epigenetic factors by including patients with TRS, and the development of models incorporating a wider range of machine learning algorithms. Additionally, it should be noted that the findings of this study are specific to the Chinese Han population and need to be replicated in other ethnic groups to determine their generalizability.

## Conclusions

This study found correlations between genetic and epigenetic risks and treatment response and identified novel genetic-epigenetic interactions that impact treatment response and cortical morphology. The study also investigated various types of APDs, which broadens the generalizability of the results. A prediction model was developed to estimate treatment response to APDs, and its robustness, generalizability, and clinical utility were demonstrated. The model is more accessible than neuroimaging biomarkers, outperformed other genetic biomarkers to date, and provides highly accurate treatment response estimation equivalent to the PANSS reduction rate, which is well received by psychiatrists. It can also utilize existing genetic resources to predict treatment response without methylation profiling. Overall, this study provides a valuable tool for precision medicine and clinical decision-making in SCZ treatment. Further research in diverse populations is necessary to enhance the model's effectiveness in future studies.

## Supplementary Information

**Additional file 1.** Supplementary methods.

**Additional file 2: Table S1**. Amplicon sequences for technical replication of methylation profiling.** Table S2**. Chlorpromazine equivalent doses for the antipsychotic drugs (mg, mean±SD).** Table S3**. Top 20 risk-DMRs.** Table S4**. Top 20 RES-DMRs.** Table S5**. Enrichment analysis of gene ontology and biological pathways for ASM genes (Top 20 terms in each category).** Table S6**. Linkage disequilibrium analysis of rs11125746 from LINC01795.** Table S7**. Linkage disequilibrium analysis of rs12674515 from DDHD2.** Table S8**. Linkage disequilibrium analysis of rs28759130 from SBNO1.** Table S9**. Linkage disequilibrium analysis of rs498541 from KCNG2.** Table S10**. Linkage disequilibrium analysis of rs56370020 from RUFY1.** Table S11**. Linkage disequilibrium analysis of rs72728886 from SEMA7A.** Table S12**. Pearson correlation of methylation levels from proxy-model CpG sites between blood and brain tissues.** Table S13**. Evaluation of methylation proxy models.** Table S14**. Performance evaluation for RES-prediction models.** Fig.S1**. Statistical power of samples.** Fig.S2**. Technical replication of methylation profiling.** Fig.S3**. Correlations between PRSs.** Fig.S4**. Relationship between treatment response and PRSs.** Fig.S5**. Variable importance plot of PRSs.** Fig.S6**. Correlation of PMS to PANSS reduction rate.** Fig.S7**. Correlation of epigenetic clocks and PANSS reduction rate.** Fig.S8**. Heatmap for the distribution of CpG sites from meQTLs, risk-DMRs, RES-DMRs, and ASM genes.** Fig.S9**. Enrichment analysis of gene ontology and biological pathways for ASM genes.** Fig.S10**. Comparison between peripheral blood and brain tissues in transcription, methylation, and chromatin interaction of RUFY1.** Fig.S11**. Performance of the optimal proxyDNAm models.

## Data Availability

All datasets used and/or analyzed in this article are stored on the data server of the corresponding authors’ lab and can be accessed by E-mail request to the corresponding authors.

## Abbreviations

ANOVA: Analysis of variance
APD: Antipsychotic drug
ASM: Allele-specific methylated
AUC: Area under the curve
BEGAIN: Brain enriched guanylate kinase associated
BP: Bipolar disorder
C + G: Clinical information + GRS
C + M: Clinical information + proxyDNAm
C + P: Clinical information + PRSs
CAPEC: Chinese Antipsychotics Pharmacogenetics Consortium
CAPOC: Chinese Antipsychotics Pharmacogenomics Consortium
COLOC: Colocalization
CV: Cross validation
DDHD2: DDHD domain containing 2
DMR: Differentially methylated region
DSM-IV: Diagnostic and statistical manual of mental disorders IV
EWAS: Epigenome-wide association study
GRS: Genetic risk score
GWAS: Genome-wide association study
HC: Health control
HEIDI: Heterogeneity in dependent instruments
KLF5: KLF transcription factor 5
LINC01795: Long intergenic non-protein coding RNA 1795
LOOCV: Leave one out cross-validation
MAE: Mean absolute error
MAPE: Mean absolute percentage error
MDD: Major depressive disorder
meQTL: Methylation quantitative trait loci
MIR885: MicroRNA 885
PAI: Promoter-anchored chromatin interaction
PANSS: Positive and Negative Syndrome Scales
PMS: Polymethylation scores
PP4: Posterior probability for a shared signal
proxyDNAm: Proxy DNA methylation model
PRS: Polygenic risk scores
QRF: Quantile random forest
R2: Coefficient of determination
RES: Response
RF: Random forest
RMSE: Root mean square error
RUFY1: RUN and FYVE domain containing 1
SBNO1: Strawberry notch homolog 1
SCZ: Schizophrenia
SEMA7A: Semaphorin 7A
SGE: Schizophrenia × gene × environment project
SLC7A7: Solute carrier family 7 member 7
SMR: Summary-based Mendelian randomization
SNP: Single-nucleotide polymorphism
SVMPoly: Support vector machines with polynomial kernel
TRS: Treatment-resistant schizophrenia
TSS: Transcription start site
