# Supplementary material for: Prediction of treatment response to antipsychotic drugs for precision medicine approach to schizophrenia: randomized trials and multiomics analysis
Source: Mil Med Res. 2023 Jun 2;10:24. doi: 10.1186/s40779-023-00459-7 (PMC10236828; doi:10.1186/s40779-023-00459-7)
Supplement: Supplementary file 1 — Additional file 1. Supplementary methods. [file 40779_2023_459_MOESM1_ESM.pdf]

Supplementary methods

Genotyping and methylation quantification

Genomic DNA was extracted with the QIAamp DNA Mini Kit (QIAGEN, Hilden, Germany) from whole blood.

All participants were genotyped via Illumina Human Omni ZhongHua-8 Beadchips (Illumina, San Diego, CA, USA) for CAPOC and Sequenom MassARRAY system (San Diego, CA, USA) for CAPEC. Quality control for genotyping was conducted before the analysis: 1) samples were excluded if the genotype call rate was less than 98%, or gender discordance, or they were first-degree or second-degree relatives, or if they were genetic outliers; 2) SNPs were excluded if minor allele frequency was less than 0.01, or the genotype call rate was less than 98%, or *P* values for Hardy-Weinberg equilibrium were less than 1×10^{-5}; 3) Genotype imputation for the discovery sample was done with the pre-phasing imputation stepwise approach implemented in IMPUTE2 [1] and SHAPEIT [2] (version 2.r727); 4) Haplotypes derived from phase I of the 1000 Genomes Project (release version 3) [3] were used as references; SNPs with low imputation quality scores (info score < 0.9) were excluded; 5) Linkage disequilibrium (LD) analysis was conducted by PLINK (version 1.90) and SNPs with LD-$R^2 > 0.5$ were removed. All genomic locations are given as Genome Reference Consortium Human Build 37 (GRCh37).

Genome-wide DNA methylation was assessed using Infinium®HumanMethylationEPIC BeadChip Kit. Quality control for methylation profiling was conducted before the analysis: 1) samples with at least 5% of the probes that did not pass a 0.05 detection *P* value threshold will be filtered; 2) the probes with >0.01 detection *P* value in more than 5% samples and the probes with < 3 bead count in at least 5% samples will be removed; 3) probes with annotated SNPs together or with probes located on sex chromosomes or align to multiple locations as identified by Nordlund et al. [4] and Zhou et al. (https://zwdzwd.github.io/InfiniumAnnotation) will be removed; 4) Beta values (ranging from 0 to 1) were then generated to represent methylation ratios at a given CpG site, and higher beta values indicate higher methylation levels; 5) Technical differences between two different probe types were then normalized by beta-mixture quantile normalization method (BMIQ) [5] as implemented in the "ChAMP" R package [6]; 6) Function champ.runCombat from ChAMP R package was used to conduct the correction of batch effect; 7) Cell proportion was estimated by DNA Methylation Age Calculator and was applied into linear model by R package limma to reduce the confounding from blood cell proportion. The methylation profiling was validated by two methods including Illumina-sequencing-based BSP and Sequenom MassARRAY® Methylation.

Technical replication of DNA methylation profiling

The validation of the chip results was conducted using Illumina-sequencing-based BSP and Sequenom MassARRAY® Methylation, employing the amplicon sequences described in Supplementary Table S1. DNA methylation in one target gene was assessed by a next-generation sequencing-based BSP via the platform of Illumina-sequencing-based BSP, according to previously published method [7, 8]. In brief, BSP primers were designed using the online MethPrimer software. 1 μg of genomic DNA was converted using the ZYMO EZ DNA Methylation-Gold Kit (Zymo Research, Irvine, CA, USA) and one twentieth of the elution products were used as templates for PCR amplification with 35 cycles using KAPA 2G

Robust HotStart PCR Kit (Kapa Biosystems, Wilmington, MA, USA). For each sample, BSP products of multiple genes were pooled equally, 5'-phosphorylated, 3'-dA-tailed and ligated to barcoded adapter using T_4 DNA ligase (NEB). Barcoded libraries from all samples were sequenced on Illumina platform. For the bisulfite sequencing reads of each sample, firstly, adapters and low-quality reads were removed using software Trimmomatic-0.36. After removing the adapter sequences and filtering out the low-quality reads, the clean sequencing reads were directly aligned to the target sequences using software Bsmap (v2.73) with the default parameters, which combines genome hashing and bitwise masking to achieve fast and accurate bisulfite mapping. Methylation levels are defined as the fraction of read counts of 'C' in the total read counts of both 'C' and 'T' for each covered C site. On the basis of such read fraction, methylated cytosine was called using a binomial distribution as in the method described by Lister et al [9]. whereby a probability mass function is calculated for each methylation context (CpG, CHG, CHH). Two-tailed Fisher's Exact Test was used to identify cytosines that are differentially methylated between two samples or groups. Only those CG covered by at least 200 reads in at least one sample were considered for testing. *P* value thresholds were selected such that the number of significance level is less than 0.001.

In addition, part of BSP-verified samples was validated by Sequenom MassARRAY® Methylation. The primer was designed by the software Epidesigner provided by Sequenom company (http://www.epidesigner.com). 10mer tag was added to the left primer (forward prime) at the terminal of 5'UTR to balance the condition of PCR reactivity. T7-promoter was added to the right primer (reverse prime) at the terminal of 5'UTR for the followed in-vitro transcription. The DNA extraction and processing were same as in EPIC profiling. We followed the protocol provided by Sequenom to conduct the bisulfite treatment including amplification, dephosphorylation, in vitro transcription and RNase A cleavage, sample conditioning, and sample transfer. The result was generated by MassARRY system and was analyzed by EpiTYPER software.

Calculation of Chlorpromazine equivalent doses

Using the chlorpromazine dose of 100 mg as a reference, the equivalent doses for the other medications based on literature [10-16] are as follows: haloperidol 2 mg, perphenazine 8 mg, aripiprazole 7.5 mg, olanzapine 5 mg, quetiapine 75 mg, risperidone 1 mg, and ziprasidone 60 mg.

Methylation quantitative trait loci (meQTL) analysis

R package MatrixEQTL was utilized to conduct the cis-meQTL analysis; The detailed approach was described by Shabalin et al [17]. MatrixEQTL linear model (phenotype to genotype) is performed in cis-meQTL analysis with the covariates: sex, drug, age at trial entry, principal components that explained the 90% variance in genotype, methylation profiling batch number, and cell count measures of CD8 T cells, CD4 T cells, natural killer cells, B cells, monocytes and granulocyte estimated by DNA Methylation Age Calculator (https://dnamage.genetics.ucla.edu). The cis-meQTL analysis is carried out for SNP-CpG combination spanning<500kb and SNP-CpG pairs with *P* value$<1\times10^{-8}$ were considered as meQTL. The FDR-corrected *P*-value<0.01 of meQTL is considered as significant.

Bayesian colocalization analysis

Bayesian colocalization analysis, which tests the colocalization of multiple genetic traits, was conducted using R package coloc [18]. Firstly, the genome-wide significance ($P < 5\times10^{-8}$) loci in GWAS were selected and analysed for colocalization analysis with meQTL summary data, with variants that are

located upstream or downstream 250 kb of selected loci being included in the colocalization test, regardless of their significance. To avoid selection bias, a non-select approach was employed, with a test window in 500kb and all variants within this window would be included in colocalization test without pre-selection based on their significance. The window was shifted by 10 kb per time until all chromosomes were scanned. Variants with posterior probability (PP4) > 0.95 in both methods of colocalization are considered as colocalization signals (one common causal variants for traits).

Epigenome-wide association study

The associations between CpG sites and treatment response (PANSS reduction rate) were examined through an epigenome-wide association study (EWAS) utilizing OSCA (OmicS-data-based Complex trait Analysis) software [19]. The EWAS employed the approximate MOMENT model (Multi-component MLM-based association excluding the target), recommended by OSCA, to test the associations. Sex, drug, age at trial entry, principal components that explained the 90% variance in genotype, methylation profiling batch number, and cell count measures of CD8 T cells, CD4T cells, natural killer cells, B cells, monocytes, and granulocyte estimated by DNA Methylation Age Calculator (https://dnamage.genetics.ucla.edu) served as covariates. The thresholds of epigenome-wide significance and suggestive-level significance were set to $P < 5\times10^{-8}$ and 1×10^{-5}, respectively.

Epigenome-wide differential methylation analysis

Region-level differential methylation analysis was performed to detect differentially methylate with "ChAMP.DMR" function from ChAMP package in R and "Bumphunter" algorithm with parameters as (1) Δbeta > 0.1 (Δbeta=beta value$_{group\ A}$- beta value$_{group\ B}$; (2) 1000 times of bootstrapping for reducing the bias in sampling; (3) beta value smoothing was performed for each CpG site; (4) *P*-values were corrected by the Benjamini-Hochberg method, with the threshold set at an adjusted *P*-value < 0.01. For each DMR, a minimum number of three consecutive CpG sites were required to constitute a DMR. Gene ontology analysis in DMR genes was conducted by g:Profiler [20].

Prediction of promoter-anchored chromatin interaction

We use an analytical approach to predict promoter-anchored chromatin interaction (PAI) based on two recently developed methods, the summary-data–based Mendelian randomization (SMR) test and the heterogeneity in dependent instruments test (HEIDI) [21]. In PAI prediction, a probe within the promoter regions (the first exon, 5'UTR, transcription start site 200, transcription start site 1500) was considered as the exposure probe; a probe within 500 kb of the exposure probe was considered as the outcome probe. We used SMR to test whether the association of DNAm level between exposure probe and outcome probe is due to a set of shared causal variants and applied the HEIDI test to distinguish the pleiotropic effect from the linkage. The detected PAI with $P_{SMR} < 0.05$ and $P_{HEIDI} > 0.1$ was considered as significant. For more specific methods, please see the description by Wu et al. [21].

References:

1. Howie BN, Donnelly P, Marchini J. A flexible and accurate genotype imputation method for the next generation of genome-wide association studies. PLoS Genet. 2009;5(6):e1000529.

2. Delaneau O, Zagury JF, Marchini J. Improved whole-chromosome phasing for disease and population genetic studies. Nat Methods. 2013;10(1):5-6.

3. Genomes Project C, Auton A, Brooks LD, Durbin RM, Garrison EP, Kang HM, et al. A global reference for human genetic variation. Nature. 2015;526(7571):68-74.

4. Nordlund J, Bäcklin CL, Wahlberg P, Busche S, Berglund EC, Eloranta ML, et al. Genome-wide signatures of differential DNA methylation in pediatric acute lymphoblastic leukemia. Genome Biol. 2013;14(9):r105.

5. Zhou W, Laird PW, Shen H. Comprehensive characterization, annotation and innovative use of infinium DNA methylation BeadChip probes. Nucleic Acids Res. 2017;45(4):e22.

6. Tian Y, Morris TJ, Webster AP, Yang Z, Beck S, Feber A, et al. ChAMP: updated methylation analysis pipeline for Illumina BeadChips. Bioinformatics. 2017;33(24):3982-4.

7. Gao F, Zhang J, Jiang P, Gong D, Wang JW, Xia Y, et al. Marked methylation changes in intestinal genes during the perinatal period of preterm neonates. BMC Genomics. 2014;15(1):716.

8. Gao F, Liang H, Lu H, Wang J, Xia M, Yuan Z, et al. Global analysis of DNA methylation in hepatocellular carcinoma by a liquid hybridization capture-based bisulfite sequencing approach. Clin Epigenetics. 2015;7(1):86.

9. Lister R, Pelizzola M, Dowen RH, Hawkins RD, Hon G, Tonti-Filippini J, et al. Human DNA methylomes at base resolution show widespread epigenomic differences. Nature. 2009;462(7271):315-22.

10. Picon FA. Manual of clinical psychopharmacology. Revista de Psiquiatria do Rio Grande do Sul. 2007;29(2):239-40.

11. Jenkins JA, Kontos N. The maudsley prescribing guidelines in psychiatry, 12th ed. J Clin Psychiatry. 2016;77(4):e469.

12. Rothe PH, Heres S, Leucht S. Dose equivalents for second generation long-acting injectable antipsychotics: the minimum effective dose method. Schizophr Res. 2018;193:23-8.

13. Patel MX, Arista IA, Taylor M, Barnes TR. How to compare doses of different antipsychotics: a systematic review of methods. Schizophr Res. 2013;149(1-3):141-8.

14. Leucht S, Samara M, Heres S, Patel MX, Furukawa T, Cipriani A, et al. Dose equivalents for second-generation antipsychotic drugs: The classical mean dose method. Schizophr Bull. 2015;41(6):1397-402.

15. Leucht S, Samara M, Heres S, Patel MX, Woods SW, Davis JM. Dose equivalents for second-generation antipsychotics: The minimum effective dose method. Schizophr Bull. 2014;40(2):314-26.

16. Woods SW. Chlorpromazine equivalent doses for the newer atypical antipsychotics. J Clin Psychiatry. 2003;64(6):663-7.

17. Shabalin AA. Matrix eQTL: ultra fast eQTL analysis via large matrix operations. Bioinformatics. 2012;28(10):1353-8.

18. Giambartolomei C, Vukcevic D, Schadt EE, Franke L, Hingorani AD, Wallace C, et al. Bayesian test for colocalisation between pairs of genetic association studies using summary statistics. PLoS Genet. 2014;10(5):e1004383.

19. Zhang F, Chen W, Zhu Z, Zhang Q, Nabais MF, Qi T, et al. OSCA: a tool for omic-data-based complex trait analysis. Genome Biol. 2019;20(1):107.

20. Raudvere U, Kolberg L, Kuzmin I, Arak T, Adler P, Peterson H, et al. G:Profiler: a web server for functional enrichment analysis and conversions of gene lists (2019 update). Nucleic Acids Res. 2019;47(W1):W191-8.

21. Wu Y, Qi T, Wang H, Zhang F, Zheng Z, Phillips-Cremins JE, et al. Promoter-anchored chromatin interactions predicted from genetic analysis of epigenomic data. Nat Commun. 2020;11(1):2061.
