# Supplementary material for: Prediction of treatment response to antipsychotic drugs for precision medicine approach to schizophrenia: randomized trials and multiomics analysis
Source: Mil Med Res. 2023 Jun 2;10:24. doi: 10.1186/s40779-023-00459-7 (PMC10236828; doi:10.1186/s40779-023-00459-7)
Supplement: Supplementary file 2 — Additional file 2: Table S1. Amplicon sequences for technical replication of methylation profiling. Table S2. Chlorpromazine equivalent doses for the antipsychotic drugs (mg, mean±SD). Table S3. Top 20 risk-DMRs. Table S4. Top 20 RES-DMRs. Table S5. Enrichment analysis of gene ontology and biological pathways for ASM genes (Top 20 terms in each category). Table S6. Linkage disequilibrium analysis of rs11125746 from LINC01795. Table S7. Linkage disequilibrium analysis of rs12674515 from DDHD2. Table S8. Linkage disequilibrium analysis of rs28759130 from SBNO1. Table S9. Linkage disequilibrium analysis of rs498541 from KCNG2. Table S10. Linkage disequilibrium analysis of rs56370020 from RUFY1. Table S11. Linkage disequilibrium analysis of rs72728886 from SEMA7A. Table S12. Pearson correlation of methylation levels from proxy-model CpG sites between blood and brain tissues. Table S13. Evaluation of methylation proxy models. Table S14. Performance evaluation for RES-prediction models. Fig.S1. Statistical power of samples. Fig.S2. Technical replication of methylation profiling. Fig.S3. Correlations between PRSs. Fig.S4. Relationship between treatment response and PRSs. Fig.S5. Variable importance plot of PRSs. Fig.S6. Correlation of PMS to PANSS reduction rate. Fig.S7. Correlation of epigenetic clocks and PANSS reduction rate. Fig.S8. Heatmap for the distribution of CpG sites from meQTLs, risk-DMRs, RES-DMRs, and ASM genes. Fig.S9. Enrichment analysis of gene ontology and biological pathways for ASM genes. Fig.S10. Comparison between peripheral blood and brain tissues in transcription, methylation, and chromatin interaction of RUFY1. Fig.S11. Performance of the optimal proxyDNAm models. [file 40779_2023_459_MOESM2_ESM.docx]

# Tables

1. Amplicon sequences for technical replication of methylation profiling

| Amplicon name | Left primer | Right primer |
| --- | --- | --- |
| LHX6-9 | TTTTTTAGGTGATGGTTTAGTTAGGG | ACCTTAAAAACAAATCTAAAACCCC |
| LHX6-23 | TTTTTTTGTAATTTAAGTTTTTGAGGG | AAAAATACTTTTTCCTCCCCTT |
| LHX6-25 | GATTTTGGTTGGGTTATTATTTGG | CCCCCTCAAAAACTTAAATTACAA |
| LHX6-31 | TTAGGATTTGGAAGTAAGAGGATTTG | AAAACCCCAAATTTATCTCCAAAA |
| RUFY1-1 | GGTGAAATGTTTAGGTTGTTTTTTTT | AACCAAATACTCTCTAAAACCCAAT |
| RUFY1-3 | TTGGGATTTTTTTGTTAGGATTTTT | AACTTTTCCAACCTTCTCTCTTACC |
| RUFY1-7 | GGTAAGAGAGAAGGTTGGAAAAGTT | CTAAACCTCCTACTCCAAAAACCTC |
| RUFY1-11 | AGGAGGTTTAGTGGTTTTTTTGATT | TCACCTTTCAACCCATATTTAAAAC |

*LHX6* LIM homeobox 6, *RUFY1* RUN and FYVE domain containing 1

1. Chlorpromazine equivalent doses for the antipsychotic drugs (mg, mean ± SD)

| Antipsychotic drug | Chlorpromazine equivalent doses in discovery cohort | | | | | |
| --- | --- | --- | --- | --- | --- | --- |
|  | Overall (*n* = 2307) | | RES (*n* = 1271) | | Non-RES (*n* = 1036) | |
|  | Initial dose | Treatment dose | Initial dose | Treatment dose | Initial dose | Treatment dose |
| Olanzapine | 362.9±96.5 | 376.8±83.6 | 366.2±79.4^#^ | 372.1±83.5 | 356.9±120.8 | 384.8±83.4 |
| Haloperidol | 720.6±301.9 | 742.0±315.4 | 695.1±256.6 | 712.25±287.3 | 756.2±354.7 | 783.6±348.8 |
| Quetiapine | 839.9±200.4 | 871.6±158.8 | 854.5±155.11 | 869.5±143.9 | 824.8±238.3 | 873.9±173.3 |
| Ziprasidone | 184.8±68.0 | 202.3±52.8 | 188.3±52.4 | 195.8±53.2^*^ | 181.7±79.2 | 208.1±52.0^*^ |
| Risperidone | 463.3±132.3 | 478.9±115.8 | 481.8±104.6^#^ | 488.3±105.5 | 430.6±166.3^#^ | 462.3±130.8 |
| Aripiprazole | 294.4±103.5 | 322.3±124.2 | 301.8±78.7 | 310.9±73.5^*^ | 287.3±122.4 | 333.2±157.5^*^ |
| Perphenazine | 317.9±141.8 | 348.8±135.4 | 299.5±111.4^#^ | 324.6±116.5^*^ | 338.8±168.2^#^ | 376.4±150.1^*^ |

*RES* response, ^#^Initial dose comparison, RES vs. Non-RES, Wilcoxon test, *P* < 0.05; ^*^Treatment dose comparison, RES vs. Non-RES, Wilcoxon test, *P* < 0.05

1. Top 20 risk-DMRs

| ID | Chromosome | Start | End | Width | T-statistic | Gene |
| --- | --- | --- | --- | --- | --- | --- |
| DMR_1 | 6 | 31935801 | 31939112 | 3311 | -15.02 | *SKIV2L/STK19* |
| DMR_2 | 6 | 33265267 | 33268071 | 2804 | 12.69 | *TAPBP/RGL2* |
| DMR_3 | 12 | 115134148 | 115136308 | 2160 | 15.08 |  |
| DMR_4 | 6 | 31854251 | 31857100 | 2849 | -14.15 | *EHMT2* |
| DMR_5 | 6 | 30709670 | 30712559 | 2889 | 12.50 | *FLOT1/IER3* |
| DMR_6 | 6 | 33130696 | 33134325 | 3629 | -16.61 | *COL11A2* |
| DMR_7 | 6 | 33286244 | 33289280 | 3036 | -14.25 | *DAXX/ZBTB22* |
| DMR_8 | 6 | 30523242 | 30525527 | 2285 | 12.21 | *PRR3/GNL1* |
| DMR_9 | 11 | 66034784 | 66036115 | 1331 | 11.69 | *KLC2/RAB1B* |
| DMR_10 | 6 | 31598379 | 31601654 | 3275 | -14.49 | *BAT2* |
| DMR_11 | 6 | 32938824 | 32941389 | 2565 | 11.13 | *BRD2* |
| DMR_12 | 6 | 31632320 | 31634657 | 2337 | 11.88 | *GPANK1/CSNK2B/BAT4* |
| DMR_13 | 10 | 8094093 | 8096991 | 2898 | 12.53 | *FLJ45983/GATA3* |
| DMR_14 | 6 | 32811181 | 32813582 | 2401 | 11.46 | *PSMB8/TAP1/PSMB81* |
| DMR_15 | 20 | 36148133 | 36149750 | 1617 | -13.01 | *BLCAP* |
| DMR_16 | 17 | 1956668 | 1958478 | 1810 | 12.60 | *HIC1* |
| DMR_17 | 10 | 89621419 | 89623360 | 1941 | 10.51 | *PTEN/KILLIN* |
| DMR_18 | 6 | 28890399 | 28892379 | 1980 | 12.21 | *TRIM27* |
| DMR_19 | 6 | 32820410 | 32822565 | 2155 | 11.93 | *PSMB9* |
| DMR_20 | 6 | 32133929 | 32135396 | 1467 | -13.96 | *EGFL8* |

*SKIV2L* SKI2 subunit of superkiller complex, *STK19* serine/threonine kinase 19, *TAPBP* TAP binding protein, *RGL2* ral guanine nucleotide dissociation stimulator like 2, *EHMT2* euchromatic histone lysine methyltransferase 2, *FLOT1* flotillin 1, IER3 immediate early response 3, *COL11A2* collagen type XI alpha 2 chain, *DAXX* death domain associated protein, *ZBTB22* zinc finger and BTB domain containing 22, *PRR3* proline rich 3, *GNL1* G protein nucleolar 1, *KLC2* kinesin light chain 2, *RAB1B* member RAS oncogene family, *BAT2* HLA-B associated transcript 2, *BRD2* bromodomain containing 2, *GPANK1* G patch domain and ankyrin repeat-containing protein 1, *CSNK2B* casein kinase 2 beta, *BAT4* HLA-B associated transcript 4, *FLJ45983* hypothetical protein FLJ45983, *GATA3* GATA binding protein 3, *PSMB8* proteasome subunit beta 8, *TAP1* transporter 1 ATP-binding cassette sub-family B member, *PSMB81* proteasome subunit beta 1, *BLCAP* bladder cancer-associated protein, *HIC1* hypermethylated in cancer 1, *PTEN* phosphatase and tensin homolog, *KILLIN* killin p53-regulated DNA replication inhibitor, *TRIM27* tripartite motif containing 27, *PSMB9* proteasome subunit beta 9, *EGFL8* EGF-like domain multiple 8

1. Top 20 RES-DMRs

| ID | Chromosome | Start | End | Width | T-statistic | Gene |
| --- | --- | --- | --- | --- | --- | --- |
| DMR_1 | 6 | 33265424 | 33268071 | 2647 | -7.27 | *RGL2/TAPBP* |
| DMR_2 | 6 | 32938824 | 32941389 | 2565 | -7.80 | *BRD2* |
| DMR_3 | 11 | 66034784 | 66036115 | 1331 | -7.64 | *KLC2/RAB1B* |
| DMR_4 | 6 | 31935801 | 31939322 | 3521 | 6.23 | *STK19/SKIV2L* |
| DMR_5 | 12 | 115134688 | 115136308 | 1620 | -8.22 | *..* |
| DMR_6 | 6 | 33385056 | 33386504 | 1448 | -7.44 | *CUTA* |
| DMR_7 | 6 | 32820249 | 32822565 | 2316 | -7.14 | *PSMB9/TAP1* |
| DMR_8 | 10 | 89621419 | 89623360 | 1941 | -6.54 | *PTEN/KILLIN* |
| DMR_9 | 3 | 37033791 | 37035625 | 1834 | -8.78 | *EPM2AIP1/MLH1* |
| DMR_10 | 11 | 108092087 | 108094111 | 2024 | -9.23 | *ATM/NPAT* |
| DMR_11 | 6 | 133561614 | 133562776 | 1162 | -8.72 | *EYA4* |
| DMR_12 | 6 | 30709670 | 30712559 | 2889 | -4.99 | *FLOT1/IER3* |
| DMR_13 | 6 | 33286244 | 33289280 | 3036 | 6.76 | *DAXX/ZBTB22* |
| DMR_14 | 6 | 31632320 | 31634657 | 2337 | -5.93 | *GPANK1/CSNK2B/BAT4* |
| DMR_15 | 20 | 36155925 | 36156827 | 902 | -8.34 | *BLCAP* |
| DMR_16 | 7 | 27168609 | 27171528 | 2919 | 9.43 | *HOXA4* |
| DMR_17 | 6 | 32811495 | 32813715 | 2220 | -6.08 | *PSMB8/TAP1/PSMB8-AS1* |
| DMR_18 | 6 | 33280390 | 33282418 | 2028 | -6.71 | *TAPBP* |
| DMR_19 | 6 | 30640154 | 30641072 | 918 | -7.79 | *DHX16* |
| DMR_20 | 6 | 30180688 | 30182244 | 1556 | -6.45 | *TRIM26* |

*RGL2* ral guanine nucleotide dissociation stimulator like 2, *TAPBP* TAP binding protein, *BRD2* bromodomain containing 2, *KLC2* kinesin light chain 2, *RAB1B* member RAS oncogene family, *STK19* serine/threonine kinase 19, *SKIV2L* SKI2 subunit of superkiller complex, *CUTA* cutA divalent cation tolerance homolog, *PSMB9* proteasome subunit beta 9, *TAP1* transporter 1 ATP-binding cassette subfamily B, *PTEN* phosphatase and tensin homolog, *KILLIN* killin p53-regulated DNA replication inhibitor, *EPM2AIP1* EPM2A interacting protein 1, *MLH1* MutL homolog 1, *ATM* ATM serine/threonine kinase, *NPAT* nuclear protein coactivator of histone transcription, *EYA4* eyes absent homolog 4, *FLOT1* flotillin 1, *IER3* immediate early response 3, *DAXX* death domain associated protein, *ZBTB22* zinc finger and BTB domain containing 22, *GPANK1* G patch domain and ankyrin repeat-containing protein 1, *CSNK2B* casein kinase 2 beta, *BAT4* HLA-B associated transcript 4, *BLCAP* bladder cancer associated-protein, *HOXA4* homeobox A4, *PSMB8* proteasome subunit beta 8, *PSMB8-AS1* PSMB8 antisense RNA 1, *DHX16* DEAH-box helicase 16, *TRIM26* tripartite motif containing 26

1. Enrichment analysis of gene ontology and biological pathways for ASM genes (Top 20 terms in each category)

| Source | Term name | Term ID | Adjusted *P-*value |
| --- | --- | --- | --- |
| GO:MF | Molecular function | GO:0003674 | 1.48×10^-117^ |
| GO:MF | Binding | GO:0005488 | 2.22×10^-101^ |
| GO:MF | Protein binding | GO:0005515 | 3.40×10^-93^ |
| GO:MF | Heterocyclic compound binding | GO:1901363 | 4.83×10^-32^ |
| GO:MF | Organic cyclic compound binding | GO:0097159 | 1.73×10^-31^ |
| GO:MF | Catalytic activity | GO:0003824 | 1.35×10^-30^ |
| GO:MF | Nucleic acid binding | GO:0003676 | 3.53×10^-25^ |
| GO:MF | RNA binding | GO:0003723 | 1.22×10^-13^ |
| GO:MF | Enzyme binding | GO:0019899 | 5.76×10^-13^ |
| GO:MF | Catalytic activity, acting on a nucleic acid | GO:0140640 | 2.83×10^-11^ |
| GO:MF | Hydrolase activity | GO:0016787 | 5.89×10^-11^ |
| GO:MF | DNA binding | GO:0003677 | 3.02×10^-10^ |
| GO:MF | Catalytic activity, acting on RNA | GO:0140098 | 3.49×10^-9^ |
| GO:MF | Transcription regulator activity | GO:0140110 | 9.83×10^-9^ |
| GO:MF | Identical protein binding | GO:0042802 | 1.49×10^-8^ |
| GO:MF | Transferase activity | GO:0016740 | 2.71×10^-8^ |
| GO:MF | Ion binding | GO:0043167 | 8.16×10^-8^ |
| GO:MF | Catalytic activity, acting on a protein | GO:0140096 | 8.85×10^-8^ |
| GO:MF | ATP-dependent activity | GO:0140657 | 1.42×10^-7^ |
| GO:MF | Sequence-specific DNA binding | GO:0043565 | 4.04×10^-7^ |
| GO:BP | Biological process | GO:0008150 | 1.62×10^-119^ |
| GO:BP | Cellular process | GO:0009987 | 4.52×10^-114^ |
| GO:BP | Metabolic process | GO:0008152 | 3.10×10^-74^ |
| GO:BP | Organic substance metabolic process | GO:0071704 | 6.92×10^-72^ |
| GO:BP | Cellular metabolic process | GO:0044237 | 1.02×10^-70^ |
| GO:BP | Primary metabolic process | GO:0044238 | 1.81×10^-70^ |
| GO:BP | Nitrogen compound metabolic process | GO:0006807 | 4.84×10^-67^ |
| GO:BP | Macromolecule metabolic process | GO:0043170 | 2.26×10^-61^ |
| GO:BP | Biological regulation | GO:0065007 | 1.38×10^-53^ |
| GO:BP | Cellular nitrogen compound metabolic process | GO:0034641 | 7.67×10^-51^ |
| GO:BP | Organic cyclic compound metabolic process | GO:1901360 | 3.48×10^-48^ |
| GO:BP | Regulation of biological process | GO:0050789 | 1.68×10^-46^ |
| GO:BP | Heterocycle metabolic process | GO:0046483 | 1.05×10^-45^ |
| GO:BP | Cellular aromatic compound metabolic process | GO:0006725 | 2.11×10^-45^ |
| GO:BP | Nucleobase-containing compound metabolic process | GO:0006139 | 3.54×10^-45^ |
| GO:BP | Gene expression | GO:0010467 | 7.51×10^-45^ |
| GO:BP | Nucleic acid metabolic process | GO:0090304 | 2.31×10^-42^ |
| GO:BP | Regulation of cellular process | GO:0050794 | 1.52×10^-40^ |
| GO:BP | Cellular macromolecule metabolic process | GO:0044260 | 3.27×10^-38^ |
| GO:BP | Biosynthetic process | GO:0009058 | 4.30×10^-38^ |
| GO:CC | Cellular component | GO:0005575 | 1.61×10^-110^ |
| GO:CC | Cellular anatomical entity | GO:0110165 | 2.05×10^-106^ |
| GO:CC | Intracellular anatomical structure | GO:0005622 | 5.33×10^-99^ |
| GO:CC | Intracellular organelle | GO:0043229 | 1.07×10^-87^ |
| GO:CC | Organelle | GO:0043226 | 3.22×10^-87^ |
| GO:CC | Membrane-bounded organelle | GO:0043227 | 6.73×10^-86^ |
| GO:CC | Intracellular membrane-bounded organelle | GO:0043231 | 3.33×10^-83^ |
| GO:CC | Cytoplasm | GO:0005737 | 3.24×10^-64^ |
| GO:CC | Membrane-enclosed lumen | GO:0031974 | 1.66×10^-47^ |
| GO:CC | Intracellular organelle lumen | GO:0070013 | 1.66×10^-47^ |
| GO:CC | Organelle lumen | GO:0043233 | 1.66×10^-47^ |
| GO:CC | Nucleus | GO:0005634 | 6.89×10^-47^ |
| GO:CC | Protein-containing complex | GO:0032991 | 1.36×10^-41^ |
| GO:CC | Nuclear lumen | GO:0031981 | 2.26×10^-37^ |
| GO:CC | Nucleoplasm | GO:0005654 | 1.93×10^-36^ |
| GO:CC | Cytosol | GO:0005829 | 1.87×10^-24^ |
| GO:CC | Intracellular non-membrane-bounded organelle | GO:0043232 | 7.97×10^-24^ |
| GO:CC | Non-membrane-bounded organelle | GO:0043228 | 8.09×10^-24^ |
| GO:CC | Membrane | GO:0016020 | 5.40×10^-23^ |
| GO:CC | Endomembrane system | GO:0012505 | 1.67×10^-18^ |
| KEGG | KEGG root term | KEGG:00000 | 3.64×10^-40^ |
| KEGG | Parkinson disease | KEGG:05012 | 4.09×10^-7^ |
| KEGG | Pathways in cancer | KEGG:05200 | 9.42×10^-7^ |
| KEGG | Alzheimer disease | KEGG:05010 | 3.70×10^-6^ |
| KEGG | Epstein-Barr virus infection | KEGG:05169 | 6.70×10^-6^ |
| KEGG | P53 signaling pathway | KEGG:04115 | 1.24×10^-5^ |
| KEGG | Metabolic pathways | KEGG:01100 | 2.84×10^-5^ |
| KEGG | Pathways of neurodegeneration - multiple diseases | KEGG:05022 | 5.00×10^-5^ |
| KEGG | Endocrine resistance | KEGG:01522 | 5.87×10^-5^ |
| KEGG | Prion disease | KEGG:05020 | 6.35×10^-5^ |
| KEGG | Human papillomavirus infection | KEGG:05165 | 8.20×10^-5^ |
| KEGG | Amyotrophic lateral sclerosis | KEGG:05014 | 1.19×10^-4^ |
| KEGG | Aminoacyl-tRNA biosynthesis | KEGG:00970 | 3.45×10^-4^ |
| KEGG | Bladder cancer | KEGG:05219 | 3.45×10^-4^ |
| KEGG | Thyroid hormone signaling pathway | KEGG:04919 | 3.71×10^-4^ |
| KEGG | Measles | KEGG:05162 | 9.11×10^-4^ |
| KEGG | Focal adhesion | KEGG:04510 | 9.34×10^-4^ |
| KEGG | Small cell lung cancer | KEGG:05222 | 1.08×10^-3^ |
| KEGG | PI3K-Akt signaling pathway | KEGG:04151 | 1.37×10^-3^ |
| KEGG | Endometrial cancer | KEGG:05213 | 1.82×10^-3^ |
| REAC | REACTOME root term | REAC:0000000 | 2.03×10^-71^ |
| REAC | Gene expression (transcription) | REAC:R-HSA-74160 | 3.00×10^-23^ |
| REAC | Metabolism of proteins | REAC:R-HSA-392499 | 2.08×10^-22^ |
| REAC | RNA polymerase II transcription | REAC:R-HSA-73857 | 3.46×10^-22^ |
| REAC | Generic transcription pathway | REAC:R-HSA-212436 | 5.88×10^-21^ |
| REAC | Signal transduction | REAC:R-HSA-162582 | 2.02×10^-14^ |
| REAC | Cell cycle | REAC:R-HSA-1640170 | 2.71×10^-13^ |
| REAC | Disease | REAC:R-HSA-1643685 | 1.33×10^-12^ |
| REAC | Post-translational protein modification | REAC:R-HSA-597592 | 3.81×10^-11^ |
| REAC | Transcriptional Regulation by TP53 | REAC:R-HSA-3700989 | 1.82×10^-9^ |
| REAC | Cell cycle, mitotic | REAC:R-HSA-69278 | 2.01×10^-9^ |
| REAC | Metabolism | REAC:R-HSA-1430728 | 3.10×10^-9^ |
| REAC | Metabolism of RNA | REAC:R-HSA-8953854 | 5.69×10^-8^ |
| REAC | Translation | REAC:R-HSA-72766 | 1.45×10^-6^ |
| REAC | Mitotic G_1_ phase and G_1_/S transition | REAC:R-HSA-453279 | 2.05×10^-6^ |
| REAC | S phase | REAC:R-HSA-69242 | 4.94×10^-6^ |
| REAC | G_2_/M checkpoints | REAC:R-HSA-69481 | 7.45×10^-6^ |
| REAC | G_1_/S transition | REAC:R-HSA-69206 | 1.13×10^-5^ |
| REAC | Transcriptional regulation by RUNX3 | REAC:R-HSA-8878159 | 1.47×10^-5^ |
| REAC | Regulation of PTEN stability and activity | REAC:R-HSA-8948751 | 3.48×10^-5^ |
| WP | WIKIPATHWAYS | WP:000000 | 1.88×10^-44^ |
| WP | miRNA regulation of DNA damage response | WP:WP1530 | 4.03×10^-6^ |
| WP | DNA damage response | WP:WP707 | 1.11×10^-5^ |
| WP | Small cell lung cancer | WP:WP4658 | 1.14×10^-4^ |
| WP | Ovarian infertility | WP:WP34 | 1.29×10^-4^ |
| WP | G_1_ to S cell cycle control | WP:WP45 | 1.45×10^-4^ |
| WP | Breast cancer pathway | WP:WP4262 | 2.18×10^-4^ |
| WP | Melanoma | WP:WP4685 | 2.54×10^-4^ |
| WP | MECP2 and associated Rett syndrome | WP:WP3584 | 3.30×10^-4^ |
| WP | Malignant pleural mesothelioma | WP:WP5087 | 3.69×10^-4^ |
| WP | Bladder cancer | WP:WP2828 | 4.08×10^-4^ |
| WP | Integrated cancer pathway | WP:WP1971 | 7.43×10^-4^ |
| WP | Apoptosis | WP:WP254 | 1.04×10^-3^ |
| WP | Retinoblastoma gene in cancer | WP:WP2446 | 1.11×10^-3^ |
| WP | Measles virus infection | WP:WP4630 | 1.16×10^-3^ |
| WP | Linoleic acid metabolism affected by SARS-CoV-2 | WP:WP4853 | 1.17×10^-3^ |
| WP | Metabolism of alpha-linolenic acid | WP:WP4586 | 1.17×10^-3^ |
| WP | PI3K-Akt signaling pathway | WP:WP4172 | 1.45×10^-3^ |
| WP | NIPBL role in DNA damage - Cornelia de Lange syndrome | WP:WP5119 | 1.86×10^-3^ |
| WP | Notch signaling pathway | WP:WP61 | 2.04×10^-3^ |
| HPA | HPA root | HPA:0000000 | 2.47×10^-67^ |
| HPA | Adrenal gland; glandular cells | HPA:0020051 | 3.38×10^-67^ |
| HPA | Adrenal gland | HPA:0020000 | 3.87×10^-67^ |
| HPA | Endometrium 2 | HPA:0170000 | 1.25×10^-66^ |
| HPA | Rectum | HPA:0400000 | 1.82×10^-66^ |
| HPA | Kidney | HPA:0270000 | 2.55×10^-66^ |
| HPA | Small intestine | HPA:0480000 | 2.82×10^-66^ |
| HPA | Cervix | HPA:0630000 | 1.74×10^-65^ |
| HPA | Bronchus | HPA:0060000 | 4.00×10^-65^ |
| HPA | Colon | HPA:0130000 | 1.38×10^-64^ |
| HPA | Duodenum | HPA:0150000 | 3.41×10^-64^ |
| HPA | Gallbladder | HPA:0220000 | 3.43×10^-64^ |
| HPA | Gallbladder; glandular cells | HPA:0220051 | 3.43×10^-64^ |
| HPA | Epididymis | HPA:0180000 | 5.85×10^-64^ |
| HPA | Epididymis; glandular cells | HPA:0180051 | 5.85×10^-64^ |
| HPA | Fallopian tube | HPA:0210000 | 7.04×10^-64^ |
| HPA | Thyroid gland | HPA:0590000 | 8.34×10^-64^ |
| HPA | Thyroid gland; glandular cells | HPA:0590051 | 8.34×10^-64^ |
| HPA | Cerebral cortex | HPA:0100000 | 1.54×10^-63^ |
| HPA | Endometrium 2; glandular cells | HPA:0170051 | 1.76×10^-63^ |
| HP | Phenotypic abnormality | HP:0000118 | 1.01×10^-35^ |
| HP | HP root | HP:0000001 | 1.13×10^-35^ |
| HP | Mode of inheritance | HP:0000005 | 1.17×10^-35^ |
| HP | Abnormality of the musculoskeletal system | HP:0033127 | 1.65×10^-33^ |
| HP | Abnormality of the nervous system | HP:0000707 | 5.67×10^-31^ |
| HP | Abnormality of head or neck | HP:0000152 | 1.01×10^-30^ |
| HP | Abnormality of the skeletal system | HP:0000924 | 1.24×10^-29^ |
| HP | Abnormal skeletal morphology | HP:0011842 | 6.14×10^-29^ |
| HP | Abnormality of the head | HP:0000234 | 1.99×10^-28^ |
| HP | Abnormality of the musculature | HP:0003011 | 1.75×10^-27^ |
| HP | Abnormality of the face | HP:0000271 | 2.79×10^-27^ |
| HP | Abnormal nervous system physiology | HP:0012638 | 5.14×10^-27^ |
| HP | Abnormality of the eye | HP:0000478 | 7.76×10^-26^ |
| HP | Abnormal nervous system morphology | HP:0012639 | 1.18×10^-25^ |
| HP | Abnormality of brain morphology | HP:0012443 | 6.54×10^-25^ |
| HP | Morphological central nervous system abnormality | HP:0002011 | 1.83×10^-24^ |
| HP | Abnormal axial skeleton morphology | HP:0009121 | 2.27×10^-24^ |
| HP | Abnormality of the cardiovascular system | HP:0001626 | 5.72×10^-24^ |
| HP | Neurodevelopmental abnormality | HP:0012759 | 4.24×10^-23^ |
| HP | Abnormality of the digestive system | HP:0025031 | 8.42×10^-23^ |

*GO* gene ontology, *GO:MF* GO molecular function, *GO:BP* GO biological process, *GO:CC* GO cellular component, *KEGG* kyoto encyclopedia of genes and genomes, *REAC* reactome, *WP* wikipathways, *HPA* human protein atlas, *CORUM* comprehensive resource of mammalian protein complexes, *HP* human phenotype ontology

1. Linkage disequilibrium analysis of rs11125746 from LINC01795

| GWAS trait | RS number | Position (GRCh37) | Alleles | *R^2^* | D' | Risk allele | *β* or *OR* | 95% CI | *P*-value |
| --- | --- | --- | --- | --- | --- | --- | --- | --- | --- |
| Waist circumference adjusted for body mass index | rs112345168 | chr2:58007905 | A=0.273, T=0.727 | 0.14 | 0.63 | .. | 0.02 | 0.016-0.029 | 2.00×10^-12^ |
| Alcohol consumption (drinks per week) | rs1568450 | chr2:58011697 | A=0.378, G=0.622 | 0.16 | 0.53 | 0.37 | 0.01 | 0.01-0.019 | 1.00×10^-10^ |
| Depression | rs1568452 | chr2:58012833 | C=0.662, T=0.338 | 0.17 | 0.55 | 0.39 | 1.03 | 1.025-1.034 | 3.00×10^-40^ |
| Major depressive disorder or stroke (pleiotropy) | rs1568452 | chr2:58012833 | C=0.662, T=0.338 | 0.17 | 0.55 | .. | .. | .. | 1.00×10^-8^ |
| Alcohol use disorder (consumption score) | rs2717071 | chr2:58030962 | A=0.627, G=0.373 | 0.15 | 0.53 | 0.63 | 0.03 | 0.023-0.045 | 1.00×10^-9^ |
| Problematic alcohol use (MTAG) | rs2717018 | chr2:58035491 | C=0.374, T=0.626 | 0.15 | 0.53 | 0.63 | 0.02 | 0.012-0.021 | 5.00×10^-15^ |
| Alcohol consumption (drinks per week) (MTAG) | rs2717018 | chr2:58035491 | C=0.374, T=0.626 | 0.15 | 0.53 | 0.64 | 0.01 | 0.0089-0.0159 | 5.00×10^-12^ |
| Alcohol use disorder (consumption score) | rs2683616 | chr2:58035555 | A=0.626, G=0.374 | 0.15 | 0.53 | 0.62 | 6.22 | .. | 5.00×10^-10^ |
| Major depressive disorder | rs2717046 | chr2:58041936 | C=0.662, T=0.338 | 0.17 | 0.54 | 0.37 | 1.03 | .. | 1.00×10^-8^ |
| Language functional connectivity | rs2717046 | chr2:58041936 | C=0.662, T=0.338 | 0.17 | 0.54 | 0.38 | 7.48 | (-) | 8.00×10^-14^ |
| Alcohol use disorder | rs1402398 | chr2:58042241 | A=0.622, G=0.378 | 0.16 | 0.53 | 0.63 | 5.94 | .. | 3.00×10^-9^ |
| Problematic alcohol use | rs1402398 | chr2:58042241 | A=0.622, G=0.378 | 0.16 | 0.53 | 0.63 | 7.10 | .. | 1.00×10^-12^ |
| Generalized epilepsy | rs1402398 | chr2:58042241 | A=0.622, G=0.378 | 0.16 | 0.53 | 0.36 | 6.79 | .. | 1.00×10^-11^ |
| Childhood absence epilepsy | rs12185644 | chr2:58051769 | A=0.634, C=0.366 | 0.16 | 0.56 | 0.29 | 6.24 | .. | 5.00×10^-10^ |
| Epilepsy | rs2947349 | chr2:58059803 | A=0.633, C=0.367 | 0.17 | 0.56 | 0.26 | 1.23 | 1.16-1.31 | 1.00×10^-8^ |
| Brain rsFMRI measurement (functional connectivity) (Net100_Pair21_49) | rs2717077 | chr2:58062489 | G=0.364, T=0.636 | 0.16 | 0.50 | .. | 0.05 | .. | 3.00×10^-11^ |
| Fish- and plant-related diet | rs17049185 | chr2:58072660 | G=0.755, T=0.245 | 0.15 | 0.72 | .. | 0.02 | 0.011-0.022 | 3.00×10^-9^ |
| Generalized epilepsy | rs2717068 | chr2:58094873 | A=0.396, C=0.604 | 0.10 | 0.41 | 0.41 | 1.27 | 1.16-1.40 | 4.00×10^-7^ |
| Whole brain restricted directional diffusion (multivariate analysis) | rs2717068 | chr2:58094873 | A=0.396, C=0.604 | 0.10 | 0.41 | .. | .. | .. | 2.00×10^-16^ |
| Alcohol consumption (drinks per week) (MTAG) | rs2717063 | chr2:58110969 | A=0.616, C=0.384 | 0.15 | 0.52 | 0.59 | 0.01 | 0.0062-0.0115 | 5.00×10^-11^ |
| Brain morphology (MOSTest) | rs2678915 | chr2:58119574 | C=0.327, T=0.673 | 0.22 | 0.71 | .. | .. | .. | 6.00×10^-17^ |
| Vertex-wise cortical surface area | rs1356544 | chr2:58123724 | C=0.673, T=0.327 | 0.22 | 0.71 | 0.41 | 11.52 | .. | 1.00×10^-30^ |
| Cortical surface area (MOSTest) | rs908730 | chr2:58135011 | A=0.654, G=0.346 | 0.20 | 0.63 | .. | .. | .. | 4.00×10^-9^ |
| Waist circumference adjusted for body mass index | rs2678904 | chr2:58137755 | G=0.327, T=0.673 | 0.23 | 0.71 | .. | .. | .. | 7.00×10^-19^ |
| Cortical surface area | rs2678904 | chr2:58137755 | G=0.327, T=0.673 | 0.23 | 0.71 | 0.40 | .. | .. | 1.00×10^-17^ |
| Brain rsFMRI measurement (functional connectivity) (Net100_Pair28_47) | rs2678904 | chr2:58137755 | G=0.327, T=0.673 | 0.23 | 0.71 | .. | 0.05 | .. | 6.00×10^-12^ |
| Brain rsFMRI measurement (functional connectivity) (Net100_Pair15_45) | rs2678871 | chr2:58153602 | A=0.667, G=0.333 | 0.22 | 0.70 | .. | 0.06 | .. | 4.00×10^-13^ |
| Vertex-wise sulcal depth | rs2678871 | chr2:58153602 | A=0.667, G=0.333 | 0.22 | 0.70 | 0.41 | 16.16 | .. | 9.00×10^-59^ |
| Whole brain restricted isotropic diffusion (multivariate analysis) | rs2139053 | chr2:58156539 | C=0.688, T=0.312 | 0.22 | 0.73 | .. | .. | .. | 1.00×10^-16^ |
| Cortical thickness | rs2678880 | chr2:58158283 | C=0.328, T=0.672 | 0.23 | 0.71 | 0.40 | .. | .. | 1.00×10^-17^ |
| Vertex-wise cortical thickness | rs2678880 | chr2:58158283 | C=0.328, T=0.672 | 0.23 | 0.71 | 0.41 | 14.70 | .. | 6.00×10^-49^ |
| Neuroticism conditioned on average household income before tax (multi-trait conditioning and joint analysis) | rs2717031 | chr2:58166468 | C=0.675, T=0.325 | 0.22 | 0.71 | .. | .. | .. | 4.00×10^-11^ |
| Neuroticism conditioned on educational attainment (multi-trait conditioning and joint analysis) | rs2717031 | chr2:58166468 | C=0.675, T=0.325 | 0.22 | 0.71 | .. | .. | .. | 3.00×10^-11^ |
| Neuroticism conditioned on cognitive performance (multi-trait conditioning and joint analysis) | rs2717031 | chr2:58166468 | C=0.675, T=0.325 | 0.22 | 0.71 | .. | .. | .. | 1.00×10^-10^ |
| Irritable mood | rs2717033 | chr2:58166769 | C=0.676, T=0.324 | 0.23 | 0.72 | 0.39 | 5.55 | .. | 3.00×10^-8^ |
| Subjective well-being (MTAG) | rs2678892 | chr2:58167140 | A=0.675, G=0.325 | 0.22 | 0.71 | 0.40 | 0.01 | 0.0067-0.0141 | 3.00×10^-8^ |
| Neuroticism conditioned on self-rated math ability (multi-trait conditioning and joint analysis) | rs2678893 | chr2:58167143 | A=0.33, G=0.67 | 0.22 | 0.70 | .. | .. | .. | 1.00×10^-8^ |
| Neuroticism conditioned on highest math class (multi-trait conditioning and joint analysis) | rs2678893 | chr2:58167143 | A=0.33, G=0.67 | 0.22 | 0.70 | .. | .. | .. | 8.00×10^-10^ |
| Neuroticism conditioned on Townsend deprivation index (multi-trait conditioning and joint analysis) | rs2678893 | chr2:58167143 | A=0.33, G=0.67 | 0.22 | 0.70 | .. | .. | .. | 6.00×10^-11^ |
| Depressive symptoms (MTAG) | rs2717036 | chr2:58168098 | C=0.675, T=0.325 | 0.22 | 0.71 | 0.39 | 0.01 | 0.01-0.019 | 1.00×10^-11^ |
| Neuroticism (MTAG) | rs2717036 | chr2:58168098 | C=0.675, T=0.325 | 0.22 | 0.71 | 0.40 | 0.02 | 0.013-0.024 | 6.00×10^-11^ |
| Depressive symptoms | rs2717036 | chr2:58168098 | C=0.675, T=0.325 | 0.22 | 0.71 | 0.39 | 0.01 | 0.01-0.02 | 9.00×10^-10^ |
| Experiencing mood swings | rs2678897 | chr2:58169418 | A=0.675, G=0.325 | 0.22 | 0.71 | 0.39 | 5.93 | .. | 3.00×10^-9^ |
| Neuroticism | rs2678897 | chr2:58169418 | A=0.675, G=0.325 | 0.22 | 0.71 | .. | 0.07 | 0.045-0.103 | 4.00×10^-7^ |
| Depressive symptoms | rs2678897 | chr2:58169418 | A=0.675, G=0.325 | 0.22 | 0.71 | 0.61 | 0.01 | 0.0046-0.0085 | 2.00×10^-11^ |
| Neuroticism | rs2678897 | chr2:58169418 | A=0.675, G=0.325 | 0.22 | 0.71 | 0.61 | 0.01 | 0.0073-0.0134 | 2.00×10^-11^ |
| Well-being spectrum (multivariate analysis) | rs2678897 | chr2:58169418 | A=0.675, G=0.325 | 0.22 | 0.71 | 0.61 | 0.01 | 0.0046-0.0083 | 8.00×10^-12^ |
| Self-reported math ability (MTAG) | rs2678897 | chr2:58169418 | A=0.675, G=0.325 | 0.22 | 0.71 | 0.61 | 0.01 | 0.01-0.017 | 2.00×10^-13^ |
| Self-reported math ability | rs2678897 | chr2:58169418 | A=0.675, G=0.325 | 0.22 | 0.71 | 0.39 | 0.02 | 0.012-0.019 | 9.00×10^-15^ |
| Neuroticism | rs2678897 | chr2:58169418 | A=0.675, G=0.325 | 0.22 | 0.71 | .. | .. | .. | 2.00×10^-11^ |
| Smoking initiation | rs1518393 | chr2:58171220 | A=0.321, C=0.679 | 0.22 | 0.72 | .. | 0.98 | .. | 4.00×10^-8^ |
| Smoking initiation (ever regular vs never regular) (MTAG) | rs1518393 | chr2:58171220 | A=0.321, C=0.679 | 0.22 | 0.72 | 0.62 | 0.01 | 0.0061-0.0105 | 1.00×10^-13^ |
| Smoking initiation (ever regular vs never regular) | rs1518393 | chr2:58171220 | A=0.321, C=0.679 | 0.22 | 0.72 | 0.62 | 0.02 | 0.012-0.022 | 1.00×10^-10^ |
| Daytime nap | rs1518394 | chr2:58171287 | A=0.32, G=0.68 | 0.22 | 0.72 | .. | 0.00 | 0.0031-0.0067 | 1.00×10^-8^ |
| Cortical surface area (min-P) | rs2717041 | chr2:58172107 | C=0.324, T=0.676 | 0.23 | 0.72 | .. | .. | .. | 2.00×10^-8^ |
| Bread consumption (slices per week) (UKB data field 1438) | rs2717041 | chr2:58172107 | C=0.324, T=0.676 | 0.23 | 0.72 | 0.41 | 0.02 | 0.013-0.021 | 3.00×10^-15^ |
| Neurociticism | rs2717043 | chr2:58179019 | C=0.324, T=0.676 | 0.23 | 0.72 | 0.37 | 6.37 | .. | 2.00×10^-10^ |
| Depressed affect | rs2717043 | chr2:58179019 | C=0.324, T=0.676 | 0.23 | 0.72 | .. | 0.02 | 0.011-0.02 | 2.00×10^-10^ |
| Depression | rs2717043 | chr2:58179019 | C=0.324, T=0.676 | 0.23 | 0.72 | 0.39 | 0.02 | 0.02-0.03 | 7.00×10^-27^ |
| Depressive symptoms | rs12713370 | chr2:58200748 | A=0.308, G=0.692 | 0.21 | 0.72 | .. | .. | .. | 2.00×10^-9^ |
| Depression | rs12713370 | chr2:58200748 | A=0.308, G=0.692 | 0.21 | 0.72 | .. | 6.04 | .. | 2.00×10^-9^ |
| Lamb consumption | rs10172295 | chr2:58201202 | A=0.454, G=0.546 | 0.13 | 0.37 | .. | 0.01 | 0.0089-0.0187 | 3.00×10^-8^ |
| Schizophrenia | rs1518395 | chr2:58208074 | A=0.308, G=0.692 | 0.21 | 0.72 | .. | 1.16 | 1.12-1.2 | 4.00×10^-13^ |
| Neuroticism | rs1518395 | chr2:58208074 | A=0.308, G=0.692 | 0.21 | 0.72 | .. | 1.01 | 1.01-1.01 | 1.00×10^-6^ |
| Positive affect | rs1518395 | chr2:58208074 | A=0.308, G=0.692 | 0.21 | 0.72 | 0.61 | 0.01 | 0.0051-0.0098 | 4.00×10^-10^ |
| Life satisfaction | rs1518395 | chr2:58208074 | A=0.308, G=0.692 | 0.21 | 0.72 | 0.61 | 0.01 | 0.0052-0.0105 | 7.00×10^-9^ |
| Major depressive disorder | rs1518395 | chr2:58208074 | A=0.308, G=0.692 | 0.21 | 0.72 | 0.61 | 0.03 | 0.02-0.044 | 4.00×10^-12^ |
| Schizophrenia | rs1518395 | chr2:58208074 | A=0.308, G=0.692 | 0.21 | 0.72 | .. | 1.14 | 1.1-1.17 | 1.00×10^-12^ |
| Waist-hip ratio | rs6717480 | chr2:58210522 | A=0.457, C=0.543 | 0.14 | 0.39 | .. | .. | .. | 1.00×10^-9^ |
| Feeling miserable | rs2312147 | chr2:58222928 | C=0.675, T=0.325 | 0.24 | 0.74 | 0.38 | 7.34 | .. | 2.00×10^-13^ |
| Schizophrenia | rs2312147 | chr2:58222928 | C=0.675, T=0.325 | 0.24 | 0.74 | .. | 1.09 | .. | 3.00×10^-7^ |
| A body shape index | rs7573056 | chr2:58229428 | A=0.463, C=0.537 | 0.31 | 0.62 | .. | 0.02 | 0.013-0.025 | 2.00×10^-9^ |
| Age at first sexual intercourse | rs961522 | chr2:58272502 | C=0.585, T=0.415 | 0.11 | 0.42 | 0.39 | 0.03 | 0.022-0.033 | 9.00×10^-23^ |
| Hip circumference adjusted for BMI | rs961522 | chr2:58272502 | C=0.585, T=0.415 | 0.11 | 0.42 | .. | 0.02 | 0.013-0.024 | 1.00×10^-10^ |
| Waist-to-hip ratio adjusted for BMI | rs986512 | chr2:58275666 | A=0.586, G=0.414 | 0.11 | 0.41 | 0.64 | 0.01 | 0.0075-0.0149 | 2.00×10^-9^ |
| Hirschsprung disease | rs4672229 | chr2:58276280 | A=0.454, C=0.546 | 0.16 | 0.42 | .. | 2.48 | 1.80-3.41 | 3.00×10^-8^ |
| Highest math class taken | rs10205421 | chr2:58297664 | A=0.675, G=0.325 | 0.26 | 0.77 | 0.34 | 0.02 | 0.012-0.022 | 5.00×10^-13^ |
| Highest math class taken (MTAG) | rs10205421 | chr2:58297664 | A=0.675, G=0.325 | 0.26 | 0.77 | 0.66 | 0.01 | 0.0068-0.0134 | 7.00×10^-9^ |
| Experiencing mood swings | rs10188070 | chr2:58299326 | A=0.32, G=0.68 | 0.28 | 0.80 | 0.35 | 5.66 | .. | 2.00×10^-8^ |
| Neuroticism | rs10188070 | chr2:58299326 | A=0.32, G=0.68 | 0.28 | 0.80 | 0.35 | 6.19 | .. | 6.00×10^-10^ |
| Neuroticism | rs10188070 | chr2:58299326 | A=0.32, G=0.68 | 0.28 | 0.80 | .. | 6.08 | .. | 1.00×10^-9^ |
| Schizophrenia | rs1051061 | chr2:58316814 | A=0.588, G=0.412 | 0.56 | 0.86 | .. | 1.17 | 1.12-1.22 | 1.00×10^-10^ |
| Insomnia symptoms (never/rarely vs. sometimes/usually) | rs12713372 | chr2:58377014 | C=0.477, T=0.523 | 0.68 | 0.82 | 0.57 | 1.02 | 1.01-1.03 | 8.00×10^-9^ |
| Insomnia symptoms (never/rarely vs. usually) | rs12713372 | chr2:58377014 | C=0.477, T=0.523 | 0.68 | 0.82 | 0.57 | 1.02 | 1.01-1.03 | 8.00×10^-6^ |
| Schizophrenia | rs848293 | chr2:58382490 | A=0.496, G=0.504 | 0.61 | 0.82 | .. | 1.12 | .. | 9.00×10^-17^ |
| Depression | rs848293 | chr2:58382490 | A=0.496, G=0.504 | 0.61 | 0.82 | .. | 5.64 | .. | 2.00×10^-8^ |
| Waist circumference adjusted for body mass index | rs848293 | chr2:58382490 | A=0.496, G=0.504 | 0.61 | 0.82 | .. | 0.02 | 0.012-0.024 | 3.00×10^-9^ |
| A body shape index | rs848293 | chr2:58382490 | A=0.496, G=0.504 | 0.61 | 0.82 | .. | 0.02 | 0.013-0.025 | 1.00×10^-9^ |
| Schizophrenia | rs7596038 | chr2:58383820 | C=0.492, T=0.508 | 0.66 | 0.84 | .. | 1.09 | 1.07-1.11 | 7.00×10^-22^ |
| Neuroticism | rs7596038 | chr2:58383820 | C=0.492, T=0.508 | 0.66 | 0.84 | .. | 5.59 | .. | 2.00×10^-8^ |
| Neuroticism | rs7596038 | chr2:58383820 | C=0.492, T=0.508 | 0.66 | 0.84 | 0.43 | 5.95 | .. | 3.00×10^-9^ |
| Schizophrenia | rs7596038 | chr2:58383820 | C=0.492, T=0.508 | 0.66 | 0.84 | .. | 1.08 | 1.06-1.1 | 1.00×10^-13^ |
| Schizophrenia | rs7596038 | chr2:58383820 | C=0.492, T=0.508 | 0.66 | 0.84 | .. | 1.07 | 1.05-1.09 | 2.00×10^-9^ |
| Schizophrenia | rs7596038 | chr2:58383820 | C=0.492, T=0.508 | 0.66 | 0.84 | .. | 1.07 | 1.05-1.09 | 2.00×10^-12^ |
| Waist-to-hip ratio adjusted for BMI | rs1100190 | chr2:58399905 | C=0.297, G=0.703 | 0.28 | 0.85 | .. | 0.02 | 0.014-0.027 | 7.00×10^-10^ |
| Waist-hip index | rs1100190 | chr2:58399905 | C=0.297, G=0.703 | 0.28 | 0.85 | .. | 0.02 | 0.015-0.028 | 8.00×10^-11^ |
| Fruit consumption | rs72618695 | chr2:58462616 | C=0.712, G=0.288 | 0.27 | 0.86 | .. | 0.02 | 0.011-0.021 | 5.00×10^-10^ |
| Waist circumference adjusted for body mass index | rs2215961 | chr2:58476015 | C=0.302, G=0.698 | 0.37 | 0.89 | .. | 0.02 | 0.011-0.024 | 4.00×10^-8^ |
| Experiencing mood swings | rs11690710 | chr2:58484172 | A=0.708, G=0.292 | 0.29 | 0.88 | 0.34 | 5.60 | .. | 2.00×10^-8^ |
| Waist-to-hip ratio adjusted for BMI | rs138378949 | chr2:58491770 | C=0.687, T=0.313 | 0.41 | 0.99 | .. | 0.02 | 0.014-0.027 | 2.00×10^-9^ |
| Waist-hip index | rs138378949 | chr2:58491770 | C=0.687, T=0.313 | 0.41 | 0.99 | .. | 0.02 | 0.015-0.028 | 3.00×10^-10^ |
| Waist circumference adjusted for body mass index | rs138378949 | chr2:58491770 | C=0.687, T=0.313 | 0.41 | 0.99 | .. | 0.02 | 0.015-0.028 | 9.00×10^-11^ |
| A body shape index | rs138378949 | chr2:58491770 | C=0.687, T=0.313 | 0.41 | 0.99 | .. | 0.02 | 0.016-0.029 | 1.00×10^-11^ |
| Schizophrenia | rs9789483 | chr2:58492345 | A=0.492, G=0.508 | 0.92 | 0.99 | .. | 1.05 | .. | 1.00×10^-6^ |
| Age at first sexual intercourse | rs9789483 | chr2:58492345 | A=0.492, G=0.508 | 0.92 | 0.99 | 0.63 | 0.01 | 0.0089-0.0173 | 2.00×10^-9^ |
| Feeling miserable | rs59187049 | chr2:58494656 | C=0.313, T=0.687 | 0.41 | 0.99 | 0.30 | 6.23 | .. | 5.00×10^-10^ |
| Neuroticism | rs17398913 | chr2:58505679 | C=0.701, T=0.299 | 0.38 | 0.99 | 0.30 | 6.08 | .. | 1.00×10^-9^ |
| Neuroticism | rs17398913 | chr2:58505679 | C=0.701, T=0.299 | 0.38 | 0.99 | .. | 5.58 | .. | 2.00×10^-8^ |
| Tea consumption | rs1156588 | chr2:58515375 | A=0.807, G=0.193 | 0.18 | 0.89 | 0.79 | 0.01 | 0.0046-0.012 | 9.00×10^-6^ |
| Self-reported math ability (MTAG) | rs6708046 | chr2:58565501 | C=0.512, T=0.488 | 0.53 | 0.74 | 0.62 | 0.01 | 0.009-0.016 | 1.00×10^-11^ |
| Self-reported math ability | rs6708046 | chr2:58565501 | C=0.512, T=0.488 | 0.53 | 0.74 | 0.62 | 0.01 | 0.0087-0.0165 | 4.00×10^-10^ |
| Highest math class taken (MTAG) | rs6708046 | chr2:58565501 | C=0.512, T=0.488 | 0.53 | 0.74 | 0.62 | 0.01 | 0.0083-0.0149 | 1.00×10^-11^ |
| Educational attainment | rs7570799 | chr2:58577350 | A=0.296, G=0.704 | 0.20 | 0.71 | 0.19 | 0.02 | 0.013-0.018 | 1.00×10^-31^ |
| Cognitive performance (MTAG) | rs17049487 | chr2:58601888 | C=0.349, G=0.651 | 0.26 | 0.73 | 0.19 | 0.02 | 0.011-0.023 | 4.00×10^-9^ |
| Body mass index in physically active individuals | rs2075171 | chr2:58688907 | A=0.239, G=0.761 | 0.16 | 0.68 | 0.77 | 0.03 | 0.016-0.04/2 | 8.00×10^-6^ |
| Body mass index in physically active individuals | rs2075171 | chr2:58688907 | A=0.239, G=0.761 | 0.16 | 0.68 | 0.76 | 0.03 | 0.016-0.036/2 | 4.00×10^-7^ |
| Body mass index in physically active individuals | rs2075171 | chr2:58688907 | A=0.239, G=0.761 | 0.16 | 0.68 | 0.77 | 0.03 | 0.015-0.035/2 | 4.00×10^-7^ |
| Body mass index | rs2075171 | chr2:58688907 | A=0.239, G=0.761 | 0.16 | 0.68 | 0.77 | 0.03 | 0.017-0.035/2 | 3.00×10^-9^ |
| Body mass index | rs2075171 | chr2:58688907 | A=0.239, G=0.761 | 0.16 | 0.68 | 0.76 | 0.03 | 0.017-0.035/2 | 8.00×10^-9^ |
| Body mass index | rs2075171 | chr2:58688907 | A=0.239, G=0.761 | 0.16 | 0.68 | 0.76 | 0.03 | 0.019-0.042/2 | 1.00×10^-7^ |
| Body mass index | rs2075171 | chr2:58688907 | A=0.239, G=0.761 | 0.16 | 0.68 | 0.77 | 0.03 | 0.021-0.043/2 | 1.00×10^-8^ |
| Body mass index (joint analysis main effects and physical activity interaction) | rs2075171 | chr2:58688907 | A=0.239, G=0.761 | 0.16 | 0.68 | .. | .. | .. | 3.00×10^-7^ |
| Body mass index (joint analysis main effects and physical activity interaction) | rs2075171 | chr2:58688907 | A=0.239, G=0.761 | 0.16 | 0.68 | .. | .. | .. | 5.00×10^-8^ |
| Body mass index (joint analysis main effects and physical activity interaction) | rs2075171 | chr2:58688907 | A=0.239, G=0.761 | 0.16 | 0.68 | .. | .. | .. | 2.00×10^-8^ |
| Body mass index (joint analysis main effects and physical activity interaction) | rs2075171 | chr2:58688907 | A=0.239, G=0.761 | 0.16 | 0.68 | .. | .. | .. | 2.00×10^-8^ |
| Body mass index | rs2075171 | chr2:58688907 | A=0.239, G=0.761 | 0.16 | 0.68 | .. | 0.02 | .. | 1.00×10^-8^ |
| Predicted visceral adipose tissue | rs7608397 | chr2:58769042 | G=0.416, T=0.584 | 0.11 | 0.42 | 0.43 | 0.02 | 0.012-0.022 | 2.00×10^-11^ |
| Body mass index | rs10197655 | chr2:58791420 | A=0.577, G=0.423 | 0.11 | 0.40 | 0.60 | 0.02 | 0.014-0.026 | 4.00×10^-9^ |
| Waist-hip ratio | rs929641 | chr2:58792377 | A=0.43, G=0.57 | 0.11 | 0.41 | 0.59 | 0.01 | 0.0094-0.016 | 8.00×10^-14^ |
| Body mass index | rs929641 | chr2:58792377 | A=0.43, G=0.57 | 0.11 | 0.41 | 0.58 | 0.02 | 0.01-0.022/2 | 5.00×10^-8^ |
| Body mass index | rs929641 | chr2:58792377 | A=0.43, G=0.57 | 0.11 | 0.41 | 0.59 | 0.02 | 0.012-0.028/2 | 4.00×10^-7^ |
| Body mass index | rs929641 | chr2:58792377 | A=0.43, G=0.57 | 0.11 | 0.41 | 0.59 | 0.02 | 0.011-0.023/2 | 1.00×10^-7^ |
| Waist-hip ratio | rs929641 | chr2:58792377 | A=0.43, G=0.57 | 0.11 | 0.41 | 0.59 | 0.02 | 0.013-0.026 | 4.00×10^-9^ |
| Waist-hip ratio | rs929641 | chr2:58792377 | A=0.43, G=0.57 | 0.11 | 0.41 | 0.59 | 0.02 | 0.013-0.031 | 7.00×10^-07^ |
| Waist circumference | rs929641 | chr2:58792377 | A=0.43, G=0.57 | 0.11 | 0.41 | 0.59 | 0.02 | 0.014-0.027 | 1.00×10^-9^ |
| Waist circumference | rs929641 | chr2:58792377 | A=0.43, G=0.57 | 0.11 | 0.41 | 0.59 | 0.02 | 0.016-0.033 | 4.00×10^-8^ |
| Body mass index | rs929641 | chr2:58792377 | A=0.43, G=0.57 | 0.11 | 0.41 | .. | 0.02 | .. | 3.00×10^-14^ |
| Educational attainment (MTAG) | rs6718563 | chr2:58805945 | A=0.42, C=0.58 | 0.11 | 0.41 | 0.56 | 0.01 | 0.0054-0.0104 | 2.00×10^-9^ |
| Educational attainment | rs72813988 | chr2:58842908 | A=0.256, C=0.744 | 0.10 | 0.52 | 0.21 | 0.01 | 0.01-0.015 | 2.00×10^-23^ |
| Daytime nap | rs17049683 | chr2:58905715 | A=0.601, G=0.399 | 0.14 | 0.44 | .. | 0.01 | 0.0038-0.0074 | 7.00×10^-10^ |

*GWAS* genome-wide association study, *LINC01795* long intergenic non-protein coding RNA 1795

1. Linkage disequilibrium analysis of rs12674515 from DDHD2

| GWAS trait | RS number | Position (GRCh37) | Alleles | *R^2^* | D' | Risk allele | *β* or *OR* | 95% CI | *P*-value |
| --- | --- | --- | --- | --- | --- | --- | --- | --- | --- |
| Menopause (age at onset) | rs28807105 | chr8:37893517 | A=0.643, G=0.357 | 0.11 | 0.35 | 0.30 | 0.16 | 0.1-0.22 | 2.00×10^-8^ |
| Detectable estradiol levels | rs28807105 | chr8:37893517 | A=0.643, G=0.357 | 0.11 | 0.35 | 0.23 | 0.02 | 0.015-0.028 | 1.00×10^-9^ |
| Detectable estradiol levels in premenopausal women | rs28807105 | chr8:37893517 | A=0.643, G=0.357 | 0.11 | 0.35 | 0.23 | 0.04 | 0.022-0.051 | 7.00×10^-7^ |
| Detectable estradiol levels in postmenopausal women | rs28807105 | chr8:37893517 | A=0.643, G=0.357 | 0.11 | 0.35 | 0.23 | 0.03 | 0.022-0.045 | 9.00×10^-9^ |
| Detectable estradiol levels | rs28807105 | chr8:37893517 | A=0.643, G=0.357 | 0.11 | 0.35 | .. | 6.11 | .. | 1.00×10^-9^ |
| Menopause (age at onset) | rs2720044 | chr8:37980587 | A=0.749, C=0.251 | 0.20 | 0.53 | 0.84 | 0.29 | 0.23-0.35 | 7.00×10^-22^ |
| Systolic blood pressure | rs145151767 | chr8:38025511 | A=0.324, G=0.676 | 0.96 | 0.99 | .. | .. | .. | 4.00×10^-14^ |
| Schizophrenia | rs16887244 | chr8:38031345 | A=0.674, G=0.326 | 0.95 | 0.99 | 0.68 | 1.19 | 1.14-1.27 | 1.00×10^-10^ |
| Diastolic blood pressure | rs2306899 | chr8:38095662 | C=0.681, T=0.319 | 0.99 | 1.00 | 0.24 | 0.11 | 0.079-0.142 | 8.00×10^-12^ |
| Hypertension | rs2306899 | chr8:38095662 | C=0.681, T=0.319 | 0.99 | 1.00 | 0.24 | 7.21 | .. | 6.00×10^-13^ |
| Pulse pressure | rs2306899 | chr8:38095662 | C=0.681, T=0.319 | 0.99 | 1.00 | 0.24 | 0.13 | 0.093-0.165 | 8.00×10^-14^ |
| Systolic blood pressure | rs2306899 | chr8:38095662 | C=0.681, T=0.319 | 0.99 | 1.00 | 0.24 | 0.23 | 0.18-0.28 | 9.00×10^-21^ |
| Urate levels | rs2306899 | chr8:38095662 | C=0.681, T=0.319 | 0.99 | 1.00 | 0.24 | 0.02 | 0.011-0.019 | 2.00×10^-12^ |
| Lymphocyte counts | rs9198 | chr8:38120721 | C=0.32, T=0.68 | 1.00 | 1.00 | 0.25 | .. | .. | 3.00×10^-12^ |
| Systolic blood pressure | rs1906672 | chr8:38130025 | A=0.337, G=0.663 | 0.92 | 1.00 | 0.23 | 0.27 | 0.21-0.33 | 1.00×10^-20^ |
| Schizophrenia vs Tourette's syndrome and other tic disorders [ordinary least squares (OLS)] | rs7845911 | chr8:38135412 | C=0.319, T=0.681 | 0.99 | 1.00 | .. | 0.02 | .. | 8.00×10^-9^ |
| Red cell distribution width | rs12674515 | chr8:38137530 | A=0.679, G=0.321 | 1.00 | 1.00 | .. | .. | .. | 2.00×10^-23^ |
| Serum uric acid levels | rs57011135 | chr8:38193011 | A=0.682, C=0.318 | 0.99 | 1.00 | .. | 0.02 | 0.011-0.019 | 5.00×10^-14^ |
| Systolic blood pressure | rs11785041 | chr8:38206242 | C=0.681, T=0.319 | 0.99 | 1.00 | 0.22 | 0.24 | 0.18-0.3 | 3.00×10^-14^ |
| Schizophrenia | rs10156310 | chr8:38209129 | A=0.681, T=0.319 | 0.99 | 1.00 | .. | 1.08 | 1.05-1.1 | 6.00×10^-10^ |
| Red cell distribution width | rs138062971 | chr8:38230452 | C=0.317, G=0.683 | 0.98 | 1.00 | 0.25 | 0.02 | 0.019-0.028 | 8.00×10^-25^ |
| Pulse pressure | rs56322953 | chr8:38243227 | A=0.319, T=0.681 | 0.98 | 1.00 | 0.23 | 0.15 | 0.11-0.2 | 2.00×10^-11^ |
| Schizophrenia | rs112537273 | chr8:38248306 | C=0.32, T=0.68 | 0.99 | 1.00 | .. | 1.07 | .. | 2.00×10^-7^ |
| Schizophrenia | rs112537273 | chr8:38248306 | C=0.32, T=0.68 | 0.99 | 1.00 | .. | 1.08 | 1.06-1.1 | 1.00×10^-11^ |
| Schizophrenia | rs112537273 | chr8:38248306 | C=0.32, T=0.68 | 0.99 | 1.00 | .. | 1.08 | 1.06-1.1 | 1.00×10^-12^ |
| Schizophrenia | rs112537273 | chr8:38248306 | C=0.32, T=0.68 | 0.99 | 1.00 | .. | 1.07 | 1.04-1.09 | 1.00×10^-6^ |
| Bipolar disorder | rs12677998 | chr8:38249201 | A=0.088, G=0.912 | 0.20 | 1.00 | 0.89 | 1.09 | .. | 7.00×10^-6^ |
| Schizophrenia | rs11986274 | chr8:38259481 | C=0.351, T=0.649 | 0.86 | 0.99 | .. | 1.08 | .. | 1.00×10^-8^ |
| Systolic blood pressure | rs13248631 | chr8:38260815 | C=0.388, T=0.612 | 0.29 | 0.98 | .. | 0.23 | 0.16-0.3 | 4.00×10^-10^ |
| Schizophrenia | rs7001340 | chr8:38262233 | C=0.349, T=0.651 | 0.85 | 0.98 | .. | 1.07 | 1.06-1.09 | 2.00×10^-13^ |
| Schizophrenia (MTAG) | rs7001340 | chr8:38262233 | C=0.349, T=0.651 | 0.85 | 0.98 | 0.76 | 0.07 | 0.047-0.099 | 4.00×10^-8^ |
| Bipolar disorder (MTAG) | rs7001340 | chr8:38262233 | C=0.349, T=0.651 | 0.85 | 0.98 | 0.76 | 0.07 | 0.049-0.1 | 1.00×10^-8^ |
| Bipolar disorder (MTAG) | rs7001340 | chr8:38262233 | C=0.349, T=0.651 | 0.85 | 0.98 | .. | .. | .. | 2.00×10^-8^ |
| Nonsyndromic cleft lip with cleft palate | rs13317 | chr8:38269514 | C=0.351, T=0.649 | 0.86 | 0.99 | 0.66 | 1.18 | .. | 4.00×10^-8^ |
| Schizophrenia (MTAG) | rs4647903 | chr8:38272582 | A=0.317, C=0.683 | 0.97 | 1.00 | .. | .. | .. | 4.00×10^-8^ |
| Anorexia nervosa, attention-deficit/hyperactivity disorder, autism spectrum disorder, bipolar disorder, major depression, obsessive-compulsive disorder, schizophrenia, or Tourette syndrome (pleiotropy) | rs4647903 | chr8:38272582 | A=0.317, C=0.683 | 0.97 | 1.00 | .. | .. | .. | 2.00×10^-9^ |
| Calcium levels | rs4647903 | chr8:38272582 | A=0.317, C=0.683 | 0.97 | 1.00 | .. | 0.02 | 0.015-0.027 | 1.00×10^-12^ |
| Urate levels | rs4647903 | chr8:38272582 | A=0.317, C=0.683 | 0.97 | 1.00 | .. | 0.02 | 0.012-0.023 | 1.00×10^-9^ |
| Calcium levels | rs4647903 | chr8:38272582 | A=0.317, C=0.683 | 0.97 | 1.00 | .. | 0.02 | 0.014-0.024 | 7.00×10^-14^ |
| Serum total protein level | rs4647903 | chr8:38272582 | A=0.317, C=0.683 | 0.97 | 1.00 | .. | 0.01 | 0.0091-0.0185 | 8.00×10^-9^ |
| Height | rs3925 | chr8:38281658 | A=0.322, G=0.678 | 0.92 | 0.96 | .. | .. | .. | 3.00×10^-13^ |
| Red cell distribution width | rs3925 | chr8:38281658 | A=0.322, G=0.678 | 0.92 | 0.96 | 0.76 | .. | .. | 5.00×10^-23^ |
| Bipolar disorder (MTAG) | rs6984358 | chr8:38284581 | C=0.68, T=0.32 | 0.90 | 0.95 | .. | .. | .. | 1.00×10^-8^ |
| Calcium levels | rs10101096 | chr8:38292147 | A=0.655, C=0.345 | 0.78 | 0.93 | .. | 0.02 | 0.013-0.024 | 3.00×10^-10^ |
| Urate levels | rs10101096 | chr8:38292147 | A=0.655, C=0.345 | 0.78 | 0.93 | .. | 0.02 | 0.011-0.022 | 2.00×10^-9^ |
| Calcium levels | rs9657190 | chr8:38295928 | A=0.647, G=0.353 | 0.76 | 0.94 | 0.25 | 0.00 | 0.0013-0.0024/ | 4.00×10^-12^ |
| Autism spectrum disorder | rs60527016 | chr8:38299624 | C=0.32, T=0.68 | 0.86 | 0.93 | 0.79 | 1.19 | 1.11-1.27 | 5.00×10^-8^ |
| Autism spectrum disorder | rs60527016 | chr8:38299624 | C=0.32, T=0.68 | 0.86 | 0.93 | 0.79 | 1.08 | 1.04-1.1 | 3.00×10^-6^ |
| Cortical surface area | rs66504003 | chr8:38340154 | C=0.459, G=0.541 | 0.11 | 0.44 | 0.47 | .. | .. | 4.00×10^-8^ |

*GWAS* genome-wide association study, *DDHD2* DDHD domain containing 2

1. Linkage disequilibrium analysis of rs28759130 from SBNO1

| GWAS trait | RS number | Position (GRCh37) | Alleles | *R^2^* | D' | Risk allele | *β* or *OR* | 95% CI | *P*-value |
| --- | --- | --- | --- | --- | --- | --- | --- | --- | --- |
| IgG glycosylation | rs2270788 | chr12:123414349 | C=0.204, T=0.796 | 0.10 | 0.37 | 0.10 | 0.24 | 0.13-0.34 | 7.00×10^-6^ |
| C-reactive protein | rs113707571 | chr12:123785526 | A=0.877, C=0.123 | 0.11 | 0.52 | .. | 0.03 | 0.018-0.037 | 8.00×10^-9^ |
| Triglyceride levels in large HDL | rs113707571 | chr12:123785526 | A=0.877, C=0.123 | 0.11 | 0.52 | 0.93 | 0.05 | 0.035-0.066 | 2.00×10^-10^ |
| Waist-to-hip ratio adjusted for BMI | rs74615202 | chr12:123387633 | G=0.152, T=0.848 | 0.12 | 0.47 | .. | 0.04 | 0.03-0.056 | 7.00×10^-11^ |
| Waist circumference adjusted for body mass index | rs74615202 | chr12:123387633 | G=0.152, T=0.848 | 0.12 | 0.47 | .. | 0.04 | 0.028-0.054 | 4.00×10^-10^ |
| Waist-to-hip ratio adjusted for BMI | rs80024005 | chr12:123383147 | G=0.785, T=0.215 | 0.18 | 0.47 | .. | .. | .. | 1.00×10^-9^ |
| Insomnia | rs585522 | chr12:123550813 | A=0.166, G=0.834 | 0.22 | 0.62 | .. | 0.01 | 0.009-0.013 | 2.00×10^-23^ |
| Multiple sclerosis | rs7975763 | chr12:123604053 | C=0.835, T=0.165 | 0.23 | 0.63 | .. | 1.09 | .. | 3.00×10^-13^ |
| Type 2 diabetes | rs940904 | chr12:123491572 | A=0.826, G=0.174 | 0.23 | 0.61 | 0.26 | 0.05 | 0.034-0.066 | 2.00×10^-9^ |
| Waist-to-hip ratio adjusted for BMI | rs940904 | chr12:123491572 | A=0.826, G=0.174 | 0.23 | 0.61 | .. | 0.03 | 0.025-0.038 | 1.00×10^-22^ |
| A body shape index | rs940904 | chr12:123491572 | A=0.826, G=0.174 | 0.23 | 0.61 | .. | 0.02 | 0.016-0.029 | 3.00×10^-12^ |
| Hip index | rs940904 | chr12:123491572 | A=0.826, G=0.174 | 0.23 | 0.61 | .. | 0.02 | 0.014-0.027 | 2.00×10^-9^ |
| Waist-hip index | rs940904 | chr12:123491572 | A=0.826, G=0.174 | 0.23 | 0.61 | .. | 0.03 | 0.024-0.037 | 3.00×10^-21^ |
| Type 2 diabetes | rs4148856 | chr12:123450765 | C=0.868, G=0.132 | 0.24 | 0.74 | 0.78 | 1.05 | 1.03-1.07 | 2.00×10^-10^ |
| Venous thromboembolism | rs4148856 | chr12:123450765 | C=0.868, G=0.132 | 0.24 | 0.74 | .. | 0.06 | 0.04-0.089 | 2.00×10^-7^ |
| A body shape index | rs74418502 | chr12:123400869 | A=0.207, T=0.793 | 0.27 | 0.60 | .. | 0.04 | 0.029-0.055 | 2.00×10^-10^ |
| Waist-hip index | rs74418502 | chr12:123400869 | A=0.207, T=0.793 | 0.27 | 0.60 | .. | 0.04 | 0.03-0.056 | 4.00×10^-11^ |
| Mean platelet volume | rs12820906 | chr12:123493123 | A=0.854, G=0.146 | 0.28 | 0.75 | 0.25 | 0.03 | 0.019-0.036 | 7.00×10^-11^ |
| Body fat distribution (leg fat ratio) | rs12820906 | chr12:123493123 | A=0.854, G=0.146 | 0.28 | 0.75 | 0.24 | 0.01 | .. | 3.00×10^-8^ |
| Body fat distribution (leg fat ratio) | rs12820906 | chr12:123493123 | A=0.854, G=0.146 | 0.28 | 0.75 | 0.24 | 0.01 | .. | 1.00×10^-7^ |
| Body fat distribution (trunk fat ratio) | rs12820906 | chr12:123493123 | A=0.854, G=0.146 | 0.28 | 0.75 | 0.24 | 0.02 | .. | 2.00×10^-9^ |
| Body fat distribution (trunk fat ratio) | rs12820906 | chr12:123493123 | A=0.854, G=0.146 | 0.28 | 0.75 | 0.24 | 0.01 | .. | 5.00×10^-6^ |
| Type 2 diabetes | rs12820906 | chr12:123493123 | A=0.854, G=0.146 | 0.28 | 0.75 | 0.24 | 0.04 | 0.033-0.054 | 2.00×10^-15^ |
| Blood protein levels | rs2510885 | chr12:123614813 | C=0.855, G=0.145 | 0.29 | 0.76 | 0.75 | 0.12 | 0.07-0.179 | 9.00×10^-6^ |
| Whole brain free water diffusion (multivariate analysis) | rs2510885 | chr12:123614813 | C=0.855, G=0.145 | 0.29 | 0.76 | .. | .. | .. | 1.00×10^-11^ |
| Hip circumference adjusted for BMI | rs1727294 | chr12:123616514 | A=0.145, G=0.855 | 0.29 | 0.76 | 0.21 | 0.04 | 0.031-0.052 | 6.00×10^-14^ |
| Hip circumference adjusted for BMI | rs1727294 | chr12:123616514 | A=0.145, G=0.855 | 0.29 | 0.76 | 0.21 | 0.03 | 0.024-0.041 | 8.00×10^-14^ |
| Schizophrenia | rs7299943 | chr12:123593485 | A=0.144, T=0.856 | 0.29 | 0.77 | .. | .. | .. | 2.00×10^-8^ |
| Cortical thickness | rs6488864 | chr12:123618362 | C=0.856, G=0.144 | 0.29 | 0.77 | 0.20 | .. | .. | 3.00×10^-12^ |
| Vertex-wise cortical thickness | rs6488864 | chr12:123618362 | C=0.856, G=0.144 | 0.29 | 0.77 | 0.22 | 8.07 | .. | 7.00×10^-16^ |
| Vertex-wise cortical surface area | rs937564 | chr12:123477311 | C=0.852, T=0.148 | 0.29 | 0.76 | 0.22 | 7.76 | .. | 9.00×10^-15^ |
| General cognitive ability | rs4275659 | chr12:123447928 | C=0.682, T=0.318 | 0.42 | 0.75 | .. | 4.82 | - | 1.00×10^-6^ |
| Type 2 diabetes | rs4275659 | chr12:123447928 | C=0.682, T=0.318 | 0.42 | 0.75 | 0.69 | 0.06 | 0.036-0.079 | 2.00×10^-7^ |
| Type 2 diabetes | rs4275659 | chr12:123447928 | C=0.682, T=0.318 | 0.42 | 0.75 | 0.69 | 0.04 | 0.027-0.059 | 2.00×10^-7^ |
| Serum albumin levels | rs4275659 | chr12:123447928 | C=0.682, T=0.318 | 0.42 | 0.75 | 0.69 | 0.02 | 0.014-0.03 | 4.00×10^-8^ |
| Attention deficit hyperactivity disorder or autism spectrum disorder or intelligence (pleiotropy) | rs4275659 | chr12:123447928 | C=0.682, T=0.318 | 0.42 | 0.75 | .. | .. | .. | 3.00×10^-9^ |
| Waist-hip ratio | rs61955196 | chr12:123451018 | C=0.678, G=0.322 | 0.43 | 0.77 | .. | .. | .. | 1.00×10^-11^ |
| Alzheimer's disease or educational attainment (pleiotropy) | rs55742290 | chr12:123466111 | C=0.356, T=0.644 | 0.43 | 0.83 | 0.71 | 66.47 | .. | 1.00×10^-13^ |
| High light scatter reticulocyte count | rs61955214 | chr12:123482424 | C=0.664, T=0.336 | 0.47 | 0.83 | 0.69 | 0.02 | 0.014-0.023 | 4.00×10^-14^ |
| Macular thickness | rs61955214 | chr12:123482424 | C=0.664, T=0.336 | 0.47 | 0.83 | .. | 0.47 | 0.33-0.62 | 4.00×10^-10^ |
| High light scatter reticulocyte percentage of red cells | rs61955214 | chr12:123482424 | C=0.664, T=0.336 | 0.47 | 0.83 | 0.68 | 0.02 | 0.012-0.022 | 4.00×10^-12^ |
| Insomnia | rs61955214 | chr12:123482424 | C=0.664, T=0.336 | 0.47 | 0.83 | .. | 0.01 | 0.004-0.008 | 2.00×10^-9^ |
| Reticulocyte fraction of red cells | rs61955214 | chr12:123482424 | C=0.664, T=0.336 | 0.47 | 0.83 | 0.68 | 0.02 | 0.012-0.021 | 2.00×10^-11^ |
| Reticulocyte count | rs61955214 | chr12:123482424 | C=0.664, T=0.336 | 0.47 | 0.83 | 0.68 | 0.02 | 0.015-0.024 | 1.00×10^-15^ |
| Hip circumference adjusted for BMI | rs61955214 | chr12:123482424 | C=0.664, T=0.336 | 0.47 | 0.83 | .. | 0.02 | 0.016-0.028 | 2.00×10^-13^ |
| Hip circumference adjusted for BMI | rs61955214 | chr12:123482424 | C=0.664, T=0.336 | 0.47 | 0.83 | .. | 0.02 | 0.013-0.026 | 7.00×10^-9^ |
| Body mass index | rs3897102 | chr12:123492112 | C=0.338, T=0.662 | 0.47 | 0.84 | .. | .. | .. | 2.00×10^-9^ |
| Schizophrenia | rs1984658 | chr12:123483426 | A=0.663, G=0.337 | 0.48 | 0.84 | .. | 1.11 | .. | 9.00×10^-14^ |
| Platelet count | rs7296418 | chr12:123457619 | C=0.333, T=0.667 | 0.48 | 0.84 | .. | .. | .. | 2.00×10^-6^ |
| Household income (MTAG) | rs4148863 | chr12:123569375 | A=0.332, G=0.668 | 0.49 | 0.84 | 0.28 | 0.01 | 0.0084-0.017 | 8.00×10^-9^ |
| Intelligence (MTAG) | rs1727307 | chr12:123575742 | A=0.329, G=0.671 | 0.50 | 0.84 | .. | 0.02 | 0.017-0.03 | 5.00×10^-13^ |
| Platelet count | rs1727307 | chr12:123575742 | A=0.329, G=0.671 | 0.50 | 0.84 | .. | .. | .. | 3.00×10^-6^ |
| Intelligence | rs1727307 | chr12:123575742 | A=0.329, G=0.671 | 0.50 | 0.84 | .. | 5.93 | - | 3.00×10^-9^ |
| Pulse pressure | rs28569936 | chr12:123895263 | A=0.729, T=0.271 | 0.63 | 0.83 | 0.93 | 0.28 | 0.18-0.38 | 4.00×10^-8^ |
| Schizophrenia vs Tourette's syndrome and other tic disorders (OLS) | rs1727302 | chr12:123632930 | A=0.719, G=0.281 | 0.66 | 0.86 | .. | 0.03 | .. | 3.00×10^-16^ |
| Alzheimer's disease or educational attainment (pleiotropy) | rs1727302 | chr12:123632930 | A=0.719, G=0.281 | 0.66 | 0.86 | 0.73 | 77.26 | .. | 7.00×10^-16^ |
| Hip index | rs1727302 | chr12:123632930 | A=0.719, G=0.281 | 0.66 | 0.86 | .. | 0.02 | 0.012-0.026 | 2.00×10^-8^ |
| Schizophrenia | rs1615350 | chr12:123650335 | C=0.281, T=0.719 | 0.66 | 0.86 | .. | 1.09 | 1.07-1.11 | 6.00×10^-18^ |
| Cognitive ability, years of educational attainment or schizophrenia (pleiotropy) | rs1615350 | chr12:123650335 | C=0.281, T=0.719 | 0.66 | 0.86 | .. | 0.02 | 0.019-0.028 | 1.00×10^-25^ |
| Anorexia nervosa, attention-deficit/hyperactivity disorder, autism spectrum disorder, bipolar disorder, major depression, obsessive-compulsive disorder, schizophrenia, or Tourette syndrome (pleiotropy) | rs1615350 | chr12:123650335 | C=0.281, T=0.719 | 0.66 | 0.86 | .. | .. | .. | 5.00×10^-10^ |
| Schizophrenia | rs1615350 | chr12:123650335 | C=0.281, T=0.719 | 0.66 | 0.86 | .. | 1.09 | 1.07-1.11 | 1.00×10^-17^ |
| Schizophrenia | rs1615350 | chr12:123650335 | C=0.281, T=0.719 | 0.66 | 0.86 | .. | 1.09 | 1.06-1.11 | 4.00×10^-12^ |
| Schizophrenia (MTAG) | rs7312955 | chr12:123747783 | A=0.304, C=0.696 | 0.67 | 0.92 | .. | .. | .. | 4.00×10^-8^ |
| Schizophrenia (MTAG) | rs7312955 | chr12:123747783 | A=0.304, C=0.696 | 0.67 | 0.92 | .. | .. | .. | 4.00×10^-8^ |
| Highest math class taken | rs7312955 | chr12:123747783 | A=0.304, C=0.696 | 0.67 | 0.92 | 0.31 | 0.01 | 0.01-0.02 | 1.00×10^-9^ |
| Type 2 diabetes | rs10773000 | chr12:123736084 | G=0.688, T=0.312 | 0.70 | 0.97 | 0.67 | 0.03 | 0.026-0.042 | 6.00×10^-17^ |
| Endometriosis or asthma (pleiotropy) | rs10773000 | chr12:123736084 | G=0.688, T=0.312 | 0.70 | 0.97 | .. | 0.05 | .. | 8.00×10^-6^ |
| Waist-to-hip ratio adjusted for BMI | rs10773000 | chr12:123736084 | G=0.688, T=0.312 | 0.70 | 0.97 | .. | 0.02 | 0.019-0.031 | 1.00×10^-16^ |
| A body shape index | rs10773000 | chr12:123736084 | G=0.688, T=0.312 | 0.70 | 0.97 | .. | 0.02 | 0.013-0.025 | 2.00×10^-10^ |
| Waist-hip index | rs10773000 | chr12:123736084 | G=0.688, T=0.312 | 0.70 | 0.97 | .. | 0.02 | 0.018-0.03 | 6.00×10^-16^ |
| Hip circumference adjusted for BMI | rs10773000 | chr12:123736084 | G=0.688, T=0.312 | 0.70 | 0.97 | .. | 0.02 | 0.016-0.028 | 1.00×10^-13^ |
| Hip circumference adjusted for BMI | rs10773000 | chr12:123736084 | G=0.688, T=0.312 | 0.70 | 0.97 | .. | 0.02 | 0.014-0.026 | 7.00×10^-10^ |
| Total cholesterol levels | rs1109559 | chr12:123757861 | A=0.685, G=0.315 | 0.73 | 0.99 | 0.32 | 0.73 | .. | 3.00×10^-6^ |
| Intelligence (MTAG) | rs1109559 | chr12:123757861 | A=0.685, G=0.315 | 0.73 | 0.99 | .. | 0.02 | 0.014-0.026 | 3.00×10^-10^ |
| Total cholesterol levels | rs1109559 | chr12:123757861 | A=0.685, G=0.315 | 0.73 | 0.99 | 0.31 | 1.71 | 1.06-2.36 | 2.00×10^-7^ |
| Intelligence (MTAG) | rs2030401 | chr12:123751339 | A=0.686, G=0.314 | 0.74 | 0.99 | .. | 0.02 | 0.012-0.024 | 2.00×10^-8^ |
| General cognitive ability | rs2030401 | chr12:123751339 | A=0.686, G=0.314 | 0.74 | 0.99 | .. | 5.11 | - | 3.00×10^-7^ |
| Schizophrenia | rs61041384 | chr12:123644043 | C=0.258, T=0.742 | 0.74 | 0.87 | .. | 1.12 | 1.09-1.15 | 6.00×10^-13^ |
| Eosinophil counts | rs61953394 | chr12:123753372 | C=0.312, T=0.688 | 0.74 | 0.99 | .. | 0.02 | 0.013-0.023 | 6.00×10^-12^ |
| Alanine aminotransferase levels | rs28642812 | chr12:123893910 | C=0.696, T=0.304 | 0.76 | 0.98 | 0.72 | 7.97 | - | 2.00×10^-15^ |
| Mean corpuscular volume | rs28642812 | chr12:123893910 | C=0.696, T=0.304 | 0.76 | 0.98 | .. | 0.01 | 0.0086-0.0172 | 6.00×10^-9^ |
| General cognitive ability | rs56309603 | chr12:123895053 | C=0.306, T=0.694 | 0.76 | 0.99 | .. | 4.94 | - | 8.00×10^-7^ |
| High density lipoprotein cholesterol levels | rs28577594 | chr12:123895906 | C=0.694, G=0.306 | 0.76 | 0.99 | .. | 0.04 | 0.025-0.049 | 2.00×10^-9^ |
| Lymphocyte counts | rs28577594 | chr12:123895906 | C=0.694, G=0.306 | 0.76 | 0.99 | 0.72 | 0.03 | 0.021-0.037 | 6.00×10^-13^ |
| Triglycerides | rs28577594 | chr12:123895906 | C=0.694, G=0.306 | 0.76 | 0.99 | 0.29 | 0.02 | .. | 6.00×10^-6^ |
| Triglycerides | rs28577594 | chr12:123895906 | C=0.694, G=0.306 | 0.76 | 0.99 | .. | 0.02 | .. | 6.00×10^-8^ |
| High density lipoprotein cholesterol levels | rs28577594 | chr12:123895906 | C=0.694, G=0.306 | 0.76 | 0.99 | 0.29 | 0.03 | .. | 5.00×10^-12^ |
| High density lipoprotein cholesterol levels | rs28577594 | chr12:123895906 | C=0.694, G=0.306 | 0.76 | 0.99 | .. | 0.03 | .. | 1.00×10^-14^ |
| Mean arterial pressure | rs35556097 | chr12:123662407 | -=0.272, C=0.728 | 0.77 | 0.91 | .. | 0.02 | 0.011-0.022 | 6.00×10^-10^ |
| Schizophrenia (MTAG) | rs1790135 | chr12:123669235 | C=0.272, T=0.728 | 0.77 | 0.91 | 0.72 | 0.07 | 0.05-0.098 | 2.00×10^-9^ |
| Schizophrenia | rs2102949 | chr12:123676763 | A=0.728, G=0.272 | 0.77 | 0.91 | .. | 1.10 | .. | 2.00×10^-14^ |
| Schizophrenia vs autism spectrum disorder (OLS) | rs2102949 | chr12:123676763 | A=0.728, G=0.272 | 0.77 | 0.91 | .. | 0.02 | .. | 2.00×10^-11^ |
| Schizophrenia vs ADHD (OLS) | rs2102949 | chr12:123676763 | A=0.728, G=0.272 | 0.77 | 0.91 | .. | 0.03 | .. | 2.00×10^-13^ |
| Schizophrenia vs anorexia nervosa (OLS) | rs2102949 | chr12:123676763 | A=0.728, G=0.272 | 0.77 | 0.91 | .. | 0.02 | .. | 1.00×10^-12^ |
| Schizophrenia | rs1716180 | chr12:123682081 | A=0.728, G=0.272 | 0.77 | 0.91 | .. | 1.10 | 1.08-1.12 | 2.00×10^-23^ |
| Schizophrenia (MTAG) | rs11057189 | chr12:123688484 | G=0.728, T=0.272 | 0.77 | 0.91 | .. | .. | .. | 5.00×10^-10^ |
| HDL cholesterol | rs28496545 | chr12:123890893 | C=0.304, T=0.696 | 0.77 | 0.99 | 0.74 | 0.04 | 0.026-0.047 | 6.00×10^-12^ |
| Apolipoprotein A1 levels | rs10846497 | chr12:123761686 | A=0.692, G=0.308 | 0.77 | 1.00 | 0.91 | 0.05 | 0.043-0.056 | 5.00×10^-49^ |
| Waist circumference adjusted for body mass index | rs2851443 | chr12:123694250 | C=0.271, T=0.729 | 0.77 | 0.91 | .. | 0.02 | 0.012-0.025 | 2.00×10^-8^ |
| Albumin-globulin ratio | rs1727319 | chr12:123701353 | C=0.729, T=0.271 | 0.77 | 0.91 | .. | 0.03 | 0.02-0.042 | 2.00×10^-8^ |
| Waist-to-hip ratio adjusted for BMI | rs79652218 | chr12:123660100 | C=0.27, T=0.73 | 0.77 | 0.91 | .. | .. | .. | 9.00×10^-13^ |
| Waist circumference adjusted for body mass index | rs79652218 | chr12:123660100 | C=0.27, T=0.73 | 0.77 | 0.91 | .. | .. | .. | 9.00×10^-12^ |
| Knee osteoarthritis | rs56116847 | chr12:123835233 | A=0.699, G=0.301 | 0.79 | 0.99 | 0.36 | 1.06 | 1.04-1.08 | 3.00×10^-10^ |
| Venous thromboembolism or fibrinogen levels (pleiotropy) | rs11611680 | chr12:123817576 | C=0.3, G=0.7 | 0.79 | 0.99 | .. | .. | .. | 8.00×10^-11^ |
| Schizophrenia (MTAG) | rs2851447 | chr12:123665113 | C=0.734, G=0.266 | 0.79 | 0.92 | .. | .. | .. | 1.00×10^-9^ |
| Schizophrenia (MTAG) | rs2851447 | chr12:123665113 | C=0.734, G=0.266 | 0.79 | 0.92 | .. | .. | .. | 1.00×10^-9^ |
| Schizophrenia | rs2851447 | chr12:123665113 | C=0.734, G=0.266 | 0.79 | 0.92 | 0.26 | 1.09 | 1.07-1.12 | 2.00×10^-14^ |
| Autism spectrum disorder or schizophrenia | rs2851447 | chr12:123665113 | C=0.734, G=0.266 | 0.79 | 0.92 | .. | 1.08 | 1.05-1.1 | 8.00×10^-12^ |
| Schizophrenia | rs2851447 | chr12:123665113 | C=0.734, G=0.266 | 0.79 | 0.92 | .. | 1.09 | 1.07-1.11 | 6.00×10^-16^ |
| Type 2 diabetes | rs2851447 | chr12:123665113 | C=0.734, G=0.266 | 0.79 | 0.92 | .. | 0.04 | 0.028-0.057 | 8.00×10^-9^ |
| Insomnia | rs28498205 | chr12:123851874 | C=0.302, T=0.698 | 0.79 | 1.00 | .. | 0.01 | 0.007-0.011 | 1.00×10^-11^ |
| Lymphocyte counts | rs28533432 | chr12:123873242 | C=0.302, T=0.698 | 0.79 | 1.00 | 0.70 | 0.03 | 0.021-0.029 | 7.00×10^-33^ |
| Lymphocyte counts | rs28533432 | chr12:123873242 | C=0.302, T=0.698 | 0.79 | 1.00 | 0.70 | .. | .. | 5.00×10^-31^ |
| Eosinophil counts | rs28866344 | chr12:123885484 | A=0.299, G=0.701 | 0.79 | 0.99 | 0.91 | 0.03 | 0.021-0.036 | 8.00×10^-14^ |
| Serum albumin levels | rs11057273 | chr12:123814466 | C=0.702, T=0.298 | 0.80 | 0.99 | .. | 0.02 | 0.018-0.03 | 9.00×10^-16^ |
| Total cholesterol levels | rs11611694 | chr12:123817649 | C=0.298, T=0.702 | 0.80 | 0.99 | .. | 0.02 | 0.017-0.028 | 7.00×10^-15^ |
| HDL cholesterol levels | rs7298909 | chr12:123830939 | C=0.298, T=0.702 | 0.80 | 0.99 | .. | 0.01 | .. | 3.00×10^-17^ |
| HDL cholesterol levels | rs7298909 | chr12:123830939 | C=0.298, T=0.702 | 0.80 | 0.99 | .. | 0.01 | .. | 3.00×10^-14^ |
| HDL cholesterol levels × alcohol consumption (regular vs non-regular drinkers) interaction (2df) | rs7298909 | chr12:123830939 | C=0.298, T=0.702 | 0.80 | 0.99 | .. | .. | .. | 2.00×10^-29^ |
| HDL cholesterol levels × alcohol consumption (drinkers vs non-drinkers) interaction (2df) | rs7298909 | chr12:123830939 | C=0.298, T=0.702 | 0.80 | 0.99 | .. | .. | .. | 2.00×10^-31^ |
| HDL cholesterol levels × alcohol consumption (drinkers vs non-drinkers) interaction (2df) | rs7298909 | chr12:123830939 | C=0.298, T=0.702 | 0.80 | 0.99 | .. | .. | .. | 5.00×10^-15^ |
| HDL cholesterol levels × alcohol consumption (drinkers vs non-drinkers) interaction (2df) | rs7298909 | chr12:123830939 | C=0.298, T=0.702 | 0.80 | 0.99 | .. | .. | .. | 3.00×10^-15^ |
| HDL cholesterol levels in current drinkers | rs7298909 | chr12:123830939 | C=0.298, T=0.702 | 0.80 | 0.99 | .. | 0.01 | .. | 2.00×10^-11^ |
| HDL cholesterol levels in current drinkers | rs7298909 | chr12:123830939 | C=0.298, T=0.702 | 0.80 | 0.99 | .. | 0.01 | .. | 9.00×10^-24^ |
| HDL cholesterol levels in current drinkers | rs7298909 | chr12:123830939 | C=0.298, T=0.702 | 0.80 | 0.99 | .. | 0.01 | .. | 2.00×10^-10^ |
| HDL cholesterol levels × alcohol consumption (regular vs non-regular drinkers) interaction (2df) | rs7298909 | chr12:123830939 | C=0.298, T=0.702 | 0.80 | 0.99 | .. | .. | .. | 3.00×10^-13^ |
| HDL cholesterol levels × alcohol consumption (regular vs non-regular drinkers) interaction (2df) | rs7298909 | chr12:123830939 | C=0.298, T=0.702 | 0.80 | 0.99 | .. | .. | .. | 1.00×10^-14^ |
| HDL cholesterol levels × long total sleep time interaction (2df test) | rs7298909 | chr12:123830939 | C=0.298, T=0.702 | 0.80 | 0.99 | .. | 0.01 | 0.0055-0.0108 | 6.00×10^-12^ |
| HDL cholesterol | rs4759375 | chr12:123796238 | C=0.7, T=0.3 | 0.80 | 1.00 | 0.08 | 0.06 | .. | 3.00×10^-8^ |
| HDL cholesterol | rs4759375 | chr12:123796238 | C=0.7, T=0.3 | 0.80 | 1.00 | 0.06 | 0.86 | 0.55-1.17/ | 8.00×10^-9^ |
| HDL cholesterol levels | rs4759375 | chr12:123796238 | C=0.7, T=0.3 | 0.80 | 1.00 | 0.15 | 0.01 | 0.0056-0.0126 | 3.00×10^-7^ |
| Total cholesterol levels | rs4759375 | chr12:123796238 | C=0.7, T=0.3 | 0.80 | 1.00 | 0.90 | 0.04 | .. | 7.00×10^-6^ |
| Total cholesterol levels | rs4759375 | chr12:123796238 | C=0.7, T=0.3 | 0.80 | 1.00 | .. | 0.04 | .. | 5.00×10^-8^ |
| High density lipoprotein cholesterol levels | rs4759375 | chr12:123796238 | C=0.7, T=0.3 | 0.80 | 1.00 | .. | 0.03 | .. | 1.00×10^-9^ |
| HDL cholesterol | rs4759375 | chr12:123796238 | C=0.7, T=0.3 | 0.80 | 1.00 | 0.09 | 0.05 | 0.039-0.058-1 | 2.00×10^-24^ |
| Liver enzyme levels (gamma-glutamyl transferase) | rs4759375 | chr12:123796238 | C=0.7, T=0.3 | 0.80 | 1.00 | 0.92 | 0.01 | 0.0041-0.0078 | 6.00×10^-11^ |
| Apolipoprotein A1 levels | rs4759375 | chr12:123796238 | C=0.7, T=0.3 | 0.80 | 1.00 | 0.92 | 0.04 | 0.028-0.055 | 3.00×10^-9^ |
| Concentration of medium HDL particles | rs4759375 | chr12:123796238 | C=0.7, T=0.3 | 0.80 | 1.00 | 0.92 | 0.04 | 0.026-0.053 | 2.00×10^-8^ |
| Total cholesterol levels | rs28516750 | chr12:123867994 | A=0.7, G=0.3 | 0.80 | 1.00 | 0.90 | 0.03 | 0.022-0.039-1 | 2.00×10^-12^ |
| HDL cholesterol levels | rs10773003 | chr12:123775127 | A=0.299, G=0.701 | 0.80 | 1.00 | 0.31 | 0.03 | 0.02-0.04 | 6.00×10^-10^ |
| HDL cholesterol levels | rs10773003 | chr12:123775127 | A=0.299, G=0.701 | 0.80 | 1.00 | 0.09 | 0.04 | 0.032-0.054.. | 5.00×10^-17^ |
| HDL cholesterol levels | rs10773003 | chr12:123775127 | A=0.299, G=0.701 | 0.80 | 1.00 | 0.30 | 0.04 | 0.022-0.056 | 6.00×10^-6^ |
| Gamma glutamyl transpeptidase | rs11057248 | chr12:123781206 | C=0.299, G=0.701 | 0.80 | 1.00 | .. | 0.02 | 0.013-0.023 | 8.00×10^-11^ |
| Eosinophil counts | rs28532037 | chr12:123883406 | A=0.701, G=0.299 | 0.80 | 1.00 | 0.88 | .. | .. | 6.00×10^-16^ |
| Eosinophil counts | rs28532037 | chr12:123883406 | A=0.701, G=0.299 | 0.80 | 1.00 | 0.91 | 0.03 | 0.023-0.036 | 4.00×10^-18^ |
| Schizophrenia | rs11532322 | chr12:123731423 | A=0.258, G=0.742 | 0.84 | 0.92 | 0.32 | 1.09 | 1.06-1.13 | 2.00×10^-8^ |
| Educational attainment | rs1568427 | chr12:123738678 | A=0.259, G=0.741 | 0.84 | 0.93 | .. | .. | .. | 2.00×10^-19^ |
| Educational attainment (years of education) | rs10773002 | chr12:123746961 | A=0.259, T=0.741 | 0.85 | 0.93 | 0.25 | 0.02 | .. | 8.00×10^-18^ |
| Educational attainment (years of education) | rs10773002 | chr12:123746961 | A=0.259, T=0.741 | 0.85 | 0.93 | 0.25 | 0.02 | 0.015-0.022 | 3.00×10^-30^ |
| Educational attainment (MTAG) | rs10773002 | chr12:123746961 | A=0.259, T=0.741 | 0.85 | 0.93 | 0.25 | 0.02 | 0.016-0.022 | 2.00×10^-35^ |
| Alzheimer's disease or educational attainment (pleiotropy) | rs1969355 | chr12:123742061 | A=0.729, G=0.271 | 0.90 | 0.99 | 0.73 | 77.41 | .. | 6.00×10^-16^ |
| Cognitive performance (MTAG) | rs1969355 | chr12:123742061 | A=0.729, G=0.271 | 0.90 | 0.99 | 0.75 | 0.02 | 0.017-0.027 | 4.00×10^-17^ |
| Waist-to-hip ratio adjusted for BMI | rs1969355 | chr12:123742061 | A=0.729, G=0.271 | 0.90 | 0.99 | .. | 0.04 | 0.029-0.041 | 8.00×10^-27^ |
| A body shape index | rs1969355 | chr12:123742061 | A=0.729, G=0.271 | 0.90 | 0.99 | .. | 0.03 | 0.021-0.034 | 7.00×10^-17^ |
| Waist-hip index | rs1969355 | chr12:123742061 | A=0.729, G=0.271 | 0.90 | 0.99 | .. | 0.03 | 0.027-0.04 | 6.00×10^-25^ |
| Cognitive ability (MTAG) | rs10732573 | chr12:123743883 | C=0.729, G=0.271 | 0.90 | 0.99 | .. | 6.75 | .. | 1.00×10^-11^ |
| Educational attainment (years of education) | rs1980251 | chr12:123744955 | A=0.729, G=0.271 | 0.90 | 0.99 | .. | .. | .. | 3.00×10^-18^ |
| Waist-to-hip ratio adjusted for BMI | rs28768122 | chr12:123885974 | C=0.734, T=0.266 | 0.93 | 0.99 | .. | .. | .. | 8.00×10^-26^ |
| Educational attainment (years of education) | rs28768122 | chr12:123885974 | C=0.734, T=0.266 | 0.93 | 0.99 | 0.25 | 0.02 | 0.015-0.021 | 6.00×10^-27^ |
| Platelet-to-lymphocyte ratio | rs28768122 | chr12:123885974 | C=0.734, T=0.266 | 0.93 | 0.99 | 0.24 | .. | .. | 5.00×10^-22^ |

*GWAS* genome-wide association study, *SBNO1* strawberry notch homolog 1

1. Linkage disequilibrium analysis of rs498541 from KCNG2

| GWAS trait | RS number | Position (GRCh37) | Alleles | *R*^2^ | D' | Risk allele | *β* or *OR* | 95% CI | *P*-value |
| --- | --- | --- | --- | --- | --- | --- | --- | --- | --- |
| Schizophrenia | rs28735056 | chr18:77622879 | A=0.581, G=0.419 | 0.91 | 0.96 | .. | 1.07 | 1.05-1.08 | 1.00×10^-12^ |
| Schizophrenia | rs28735056 | chr18:77622879 | A=0.581, G=0.419 | 0.91 | 0.96 | .. | 1.06 | 1.04-1.08 | 5.00×10^-10^ |
| Schizophrenia | rs28735056 | chr18:77622879 | A=0.581, G=0.419 | 0.91 | 0.96 | .. | 1.06 | 1.04-1.08 | 5.00×10^-9^ |
| Schizophrenia | rs11665111 | chr18:77622996 | C=0.433, T=0.567 | 0.49 | 0.96 | .. | 1.09 | .. | 1.00×10^-8^ |
| Educational attainment (years of education) | rs12970264 | chr18:77627950 | A=0.379, G=0.621 | 0.42 | 0.98 | 0.44 | 0.01 | 0.0058-0.0112 | 2.00×10^-9^ |
| Educational attainment | rs12964590 | chr18:77632580 | A=0.399, G=0.601 | 0.45 | 0.98 | 0.45 | 0.01 | 0.006-0.0101 | 2.00×10^-14^ |
| Severe insulin-resistant type 2 diabetes | rs12928 | chr18:77663863 | A=0.397, G=0.603 | 0.35 | 0.87 | 0.35 | 0.76 | 0.683-0.844 | 4.00×10^-7^ |
| Schizophrenia | rs55642704 | chr18:77688124 | C=0.505, T=0.495 | 0.23 | 0.57 | .. | 1.09 | .. | 4.00×10^-11^ |

*GWAS* genome-wide association study, *KCNG2* potassium voltage-gated channel modifier subfamily G member 2

1. Linkage disequilibrium analysis of rs56370020 from RUFY1

| GWAS trait | RS number | Position (GRCh37) | Alleles | *R*^2^ | D' | Risk allele | *β* or *OR* | 95% CI | *P*-value |
| --- | --- | --- | --- | --- | --- | --- | --- | --- | --- |
| DNA methylation variation (age effect) | rs6875834 | chr5:178914711 | A=0.829, G=0.171 | 0.11 | 0.34 | .. | .. | .. | 4.00×10^-8^ |
| Lung cancer | rs10479542 | chr5:178981241 | A=0.716, T=0.284 | 0.18 | 0.57 | 0.39 | 1.06 | 1.03-1.08 | 4.00×10^-6^ |
| Metabolic biomarkers (multivariate analysis) | rs6899218 | chr5:178988608 | A=0.779, T=0.221 | 0.75 | 1.00 | 0.51 | .. | .. | 8.00×10^-10^ |
| Body fat percentage | rs6899218 | chr5:178988608 | A=0.779, T=0.221 | 0.75 | 1.00 | 0.51 | 0.01 | 0.01-0.019 | 1.00×10^-11^ |
| Cortical thickness | rs11958537 | chr5:178994087 | G=0.818, T=0.182 | 0.96 | 1.00 | 0.40 | .. | .. | 3.00×10^-9^ |
| Vertex-wise cortical thickness | rs11958537 | chr5:178994087 | G=0.818, T=0.182 | 0.96 | 1.00 | 0.38 | 6.26 | .. | 4.00×10^-10^ |
| Monocyte percentage of white cells | rs56370020 | chr5:179032355 | C=0.824, T=0.176 | 1.00 | 1.00 | 0.36 | 0.01 | 0.01-0.019 | 2.00×10^-10^ |
| Whole brain restricted directional diffusion (multivariate analysis) | rs4701140 | chr5:179034260 | A=0.784, G=0.216 | 0.72 | 0.96 | .. | .. | .. | 2.00×10^-10^ |
| Whole brain restricted isotropic diffusion (multivariate analysis) | rs4701140 | chr5:179034260 | A=0.784, G=0.216 | 0.72 | 0.96 | .. | .. | .. | 7.00×10^-11^ |
| Cortical surface area | rs61408162 | chr5:179042400 | C=0.828, T=0.172 | 0.96 | 0.99 | 0.36 | .. | .. | 7.00×10^-12^ |
| Vertex-wise cortical surface area | rs61408162 | chr5:179042400 | C=0.828, T=0.172 | 0.96 | 0.99 | 0.32 | 6.72 | .. | 2.00×10^-11^ |
| Neutrophil percentage of white cells | rs61408162 | chr5:179042400 | C=0.828, T=0.172 | 0.96 | 0.99 | 0.36 | 0.02 | 0.012-0.021 | 1.00×10^-12^ |
| Vertex-wise sulcal depth | rs61408162 | chr5:179042400 | C=0.828, T=0.172 | 0.96 | 0.99 | 0.32 | 9.47 | .. | 3.00×10^-21^ |

*GWAS* genome-wide association study, *RUFY1* RUN and FYVE domain containing 1

1. Linkage disequilibrium analysis of rs72728886 from *SEMA7A*

| GWAS trait | RS number | Position (GRCh37) | Alleles | *R*^2^ | D' | Risk allele | *β* or *OR* | 95% CI | *P*-value |
| --- | --- | --- | --- | --- | --- | --- | --- | --- | --- |
| Serum albumin levels | rs12900028 | chr15:74666027 | C=0.211, G=0.789 | 0.12 | 0.40 | .. | 0.01 | 0.0093-0.0179 | 4.00×10^-10^ |
| Alcohol consumption (drinks per week) | rs35807116 | chr15:74667953 | C=0.788, T=0.212 | 0.12 | 0.40 | 0.61 | 0.01 | 0.0094-0.0184 | 1.00×10^-9^ |
| Apolipoprotein A1 levels | rs28362901 | chr15:74712937 | A=0.464, C=0.536 | 0.14 | 0.90 | 0.91 | 0.02 | 0.018-0.031 | 9.00×10^-13^ |
| HDL cholesterol levels | rs28362901 | chr15:74712937 | A=0.464, C=0.536 | 0.14 | 0.90 | 0.91 | 0.02 | 0.017-0.031 | 1.00×10^-12^ |
| HDL cholesterol | rs28362901 | chr15:74712937 | A=0.464, C=0.536 | 0.14 | 0.90 | .. | 0.02 | 0.013-0.025 | 4.00×10^-10^ |
| High density lipoprotein cholesterol levels | rs28362901 | chr15:74712937 | A=0.464, C=0.536 | 0.14 | 0.90 | .. | 0.03 | 0.018-0.035 | 9.00×10^-10^ |
| Hypertension | rs8036030 | chr15:74716609 | A=0.398, G=0.602 | 0.26 | 0.92 | .. | 0.04 | .. | 8.00×10^-11^ |
| Airflow obstruction | rs8036030 | chr15:74716609 | A=0.398, G=0.602 | 0.26 | 0.92 | 0.61 | 1.18 | .. | 8.00×10^-6^ |
| Nonsyndromic cleft lip with cleft palate | rs2289187 | chr15:74744399 | C=0.427, T=0.573 | 0.26 | 0.98 | 0.48 | 1.21 | .. | 4.00×10^-11^ |
| Craniofacial microsomia | rs10459648 | chr15:74865440 | C=0.427, T=0.573 | 0.26 | 0.98 | 0.51 | 1.59 | 1.45-1.72 | 1.00×10^-23^ |
| Height | rs8025068 | chr15:74870045 | G=0.483, T=0.517 | 0.20 | 0.98 | .. | 0.02 | 0.018-0.029 | 2.00×10^-15^ |
| Height | rs8025068 | chr15:74870045 | G=0.483, T=0.517 | 0.20 | 0.98 | .. | 0.02 | 0.013-0.032 | 7.00×10^-6^ |
| Endometriosis | rs74781061 | chr15:74888196 | A=0.428, G=0.572 | 0.26 | 0.99 | 0.12 | 2.19 | 1.579-3.032 | 1.00×10^-6^ |
| Cleft lip with or without cleft palate | rs11072494 | chr15:74889163 | C=0.428, T=0.572 | 0.26 | 0.99 | .. | 1.19 | 1.05-1.33 | 2.00×10^-8^ |
| Nonsyndromic cleft lip with cleft palate | rs6495117 | chr15:74899500 | C=0.57, T=0.43 | 0.26 | 0.99 | 0.48 | 1.20 | .. | 6.00×10^-11^ |
| Lung adenocarcinoma | rs76354137 | chr15:75029998 | C=0.513, T=0.487 | 0.18 | 0.93 | 0.38 | 0.90 | 0.87-0.94 | 5.00×10^-7^ |
| Non-small cell lung cancer | rs76354137 | chr15:75029998 | C=0.513, T=0.487 | 0.18 | 0.93 | 0.38 | 0.92 | 0.88-0.95 | 6.00×10^-7^ |
| Coronary heart disease | rs2472299 | chr15:75033400 | A=0.324, G=0.676 | 0.23 | 0.74 | .. | .. | .. | 3.00×10^-6^ |
| Serum albumin levels | rs79755028 | chr15:75060413 | A=0.102, G=0.898 | 0.39 | 0.83 | .. | 0.04 | 0.026-0.052 | 5.00×10^-9^ |
| Medication use (beta blocking agents) | rs1543927 | chr15:75063573 | C=0.671, T=0.329 | 0.21 | 0.72 | 0.26 | 0.05 | 0.034-0.071 | 2.00×10^-8^ |
| Hypertension | rs936226 | chr15:75069282 | C=0.33, T=0.67 | 0.19 | 0.69 | .. | 0.05 | .. | 9.00×10^-15^ |
| Diastolic blood pressure | rs936226 | chr15:75069282 | C=0.33, T=0.67 | 0.19 | 0.69 | 0.72 | 0.36 | 0.28-0.44 | 1.00×10^-18^ |
| Systolic blood pressure | rs936226 | chr15:75069282 | C=0.33, T=0.67 | 0.19 | 0.69 | 0.72 | 0.55 | 0.42-0.68 | 3.00×10^-16^ |
| Waist-hip ratio | rs936226 | chr15:75069282 | C=0.33, T=0.67 | 0.19 | 0.69 | 0.73 | 0.01 | 0.0077-0.0151 | 2.00×10^-9^ |
| Waist-hip ratio | rs8033381 | chr15:75080685 | A=0.632, G=0.368 | 0.16 | 0.68 | 0.73 | 0.01 | 0.0077-0.0151 | 2.00×10^-9^ |
| Diastolic blood pressure × alcohol consumption (light vs heavy) interaction (2df test) | rs1350193 | chr15:75084281 | C=0.632, G=0.368 | 0.16 | 0.68 | 0.68 | .. | .. | 3.00×10^-13^ |
| Systolic blood pressure | rs72730503 | chr15:75086386 | A=0.63, G=0.37 | 0.16 | 0.68 | .. | 0.02 | 0.015-0.023 | 3.00×10^-20^ |
| Mean arterial pressure × alcohol consumption (light vs heavy) interaction (2df test) | rs2301249 | chr15:75092384 | C=0.631, T=0.369 | 0.14 | 0.64 | 0.32 | .. | .. | 1.00×10^-15^ |
| Systolic blood pressure × alcohol consumption (light vs heavy) interaction (2df test) | rs2301249 | chr15:75092384 | C=0.631, T=0.369 | 0.14 | 0.64 | 0.32 | .. | .. | 5.00×10^-14^ |
| Lymphocyte counts | rs2301249 | chr15:75092384 | C=0.631, T=0.369 | 0.14 | 0.64 | 0.70 | .. | .. | 2.00×10^-10^ |
| Celiac disease | rs1378938 | chr15:75096443 | C=0.633, T=0.367 | 0.15 | 0.65 | 0.28 | 1.13 | .. | 8.00×10^-9^ |
| LDL cholesterol levels | rs112987086 | chr15:75106719 | G=0.415, T=0.585 | 0.12 | 0.64 | 0.28 | 0.02 | 0.011-0.021 | 1.00×10^-11^ |
| Apolipoprotein B levels | rs112987086 | chr15:75106719 | G=0.415, T=0.585 | 0.12 | 0.64 | 0.28 | 0.01 | 0.0094-0.0186 | 3.00×10^-9^ |
| LDL cholesterol | rs112987086 | chr15:75106719 | G=0.415, T=0.585 | 0.12 | 0.64 | .. | 0.02 | 0.014-0.024 | 2.00×10^-12^ |

*GWAS* genome-wide association study, *SEMA7A* solute carrier family 7 member 7

1. Pearson correlation of methylation levels from proxy-model CpG sites between blood and brain tissues

| CpG | Source | Gene | PFC | | EC | | STG | | CER | |
| --- | --- | --- | --- | --- | --- | --- | --- | --- | --- | --- |
|  |  |  | *r* | *P*-value | *r* | *P-*value | *r* | *P*-value | *r* | *P-*value |
| cg06263834 | DMR | *SEMA7A* | 2.46×10^-2^ | 8.35×10^-1^ | 2.73×10^-1^ | 2.10×10^-2^ | 6.20×10^-2^ | 5.97×10^-1^ | 8.98×10^-2^ | 4.57×10^-1^ |
| cg08125401 | DMR | *SEMA7A* | 3.42×10^-1^ | 2.82×10^-3^ | 2.00×10^-1^ | 9.44×10^-2^ | 8.08×10^-2^ | 4.91×10^-1^ | 2.43×10^-2^ | 8.41×10^-1^ |
| cg13462576 | DMR | *SEMA7A* | 3.96×10^-2^ | 7.38×10^-1^ | -8.54×10^-2^ | 4.79×10^-1^ | 7.50×10^-2^ | 5.23×10^-1^ | 6.31×10^-2^ | 6.01×10^-1^ |
| cg14093936 | DMR | *SEMA7A* | 1.32×10^-1^ | 2.63×10^-1^ | 3.37×10^-2^ | 7.80×10^-1^ | 1.87×10^-1^ | 1.09×10^-1^ | 1.61×10^-1^ | 1.81×10^-1^ |
| cg14443380 | DMR | *SEMA7A* | -5.79×10^-2^ | 6.24×10^-1^ | 1.38×10^-1^ | 2.52×10^-1^ | 9.93×10^-2^ | 3.97×10^-1^ | 8.87×10^-2^ | 4.62×10^-1^ |
| cg18468560 | DMR | *SEMA7A* | 9.31×10^-2^ | 4.30×10^-1^ | -1.27×10^-1^ | 2.89×10^-1^ | 1.74×10^-1^ | 1.36×10^-1^ | 9.51×10^-2^ | 4.30×10^-1^ |
| cg20559000 | DMR | *SEMA7A* | -9.13×10^-2^ | 4.39×10^-1^ | -2.11×10^-1^ | 7.72×10^-2^ | 1.33×10^-1^ | 2.54×10^-1^ | -3.56×10^-2^ | 7.69×10^-1^ |
| cg20736847 | DMR | *SEMA7A* | 5.08×10^-2^ | 6.67×10^-1^ | -9.53×10^-2^ | 4.29×10^-1^ | -1.49×10^-1^ | 2.03×10^-1^ | 2.63×10^-1^ | 2.68×10^-2^ |
| cg20927731 | DMR | *SEMA7A* | 1.58×10^-1^ | 1.79×10^-1^ | 2.44×10^-1^ | 4.05×10^-2^ | 7.19×10^-2^ | 5.40×10^-1^ | 3.48×10^-1^ | 2.92×10^-3^ |
| cg22825002 | DMR | *SEMA7A* | -8.39×10^-3^ | 9.43×10^-1^ | -4.63×10^-2^ | 7.02×10^-1^ | -3.35×10^-2^ | 7.76×10^-1^ | 9.39×10^-2^ | 4.36×10^-1^ |
| cg24878071 | DMR | *SEMA7A* | 3.15×10^-2^ | 7.90×10^-1^ | 1.57×10^-1^ | 1.92×10^-1^ | -2.93×10^-3^ | 9.80×10^-1^ | 1.83×10^-2^ | 8.80×10^-1^ |
| cg26095405 | DMR | *SEMA7A* | 2.99×10^-2^ | 8.00×10^-1^ | 1.14×10^-1^ | 3.44×10^-1^ | -7.48×10^-2^ | 5.24×10^-1^ | -6.94×10^-2^ | 5.65×10^-1^ |
| cg26437123 | DMR | *SEMA7A* | 1.35×10^-1^ | 2.52×10^-1^ | 3.95×10^-3^ | 9.74×10^-1^ | 1.86×10^-2^ | 8.74×10^-1^ | 2.24×10^-1^ | 6.00×10^-2^ |
| cg13557411 | EWAS | *SEMA7A* | 1.85×10^-1^ | 1.16×10^-1^ | 1.65×10^-1^ | 1.69×10^-1^ | -6.57×10^-2^ | 5.76×10^-1^ | -1.03×10^-1^ | 3.94×10^-1^ |
| cg05964363 | Colocalization | *DDHD2* | 4.28×10^-2^ | 7.17×10^-1^ | 2.49×10^-1^ | 3.62×10^-2^ | 1.48×10^-1^ | 2.05×10^-1^ | 4.80×10^-2^ | 6.91×10^-1^ |
| cg25217636 | Colocalization | *DDHD2* | -7.04×10^-2^ | 5.51×10^-1^ | -1.67×10^-1^ | 1.64×10^-1^ | -5.79×10^-2^ | 6.22×10^-1^ | 6.86×10^-2^ | 5.70×10^-1^ |
| cg17516945 | DMR | *DDHD2* | -2.53×10^-2^ | 8.31×10^-1^ | 1.59×10^-1^ | 1.84×10^-1^ | -5.14×10^-2^ | 6.61×10^-1^ | 1.80×10^-1^ | 1.33×10^-1^ |
| cg03229959 | DMR | *DDHD2* | -2.99×10^-2^ | 8.00×10^-1^ | 1.37×10^-1^ | 2.53×10^-1^ | 1.14×10^-1^ | 3.28×10^-1^ | -1.32×10^-2^ | 9.13×10^-1^ |
| cg12619398 | DMR | *DDHD2* | -1.17×10^-1^ | 3.22×10^-1^ | 1.73×10^-1^ | 1.49×10^-1^ | -1.28×10^-1^ | 2.75×10^-1^ | 9.88×10^-2^ | 4.12×10^-1^ |
| cg04218418 | DMR | *DDHD2* | 2.33×10^-2^ | 8.44×10^-1^ | 3.43×10^-2^ | 7.76×10^-1^ | 1.75×10^-1^ | 1.33×10^-1^ | 1.27×10^-1^ | 2.92×10^-1^ |
| cg23285761 | DMR | *DDHD2* | 6.91×10^-2^ | 5.59×10^-1^ | 2.15×10^-1^ | 7.13×10^-2^ | 4.26×10^-2^ | 7.17×10^-1^ | 2.16×10^-1^ | 7.10×10^-2^ |
| cg16937126 | DMR | *DDHD2* | 3.27×10^-2^ | 7.82×10^-1^ | -6.87×10^-2^ | -6.87×10^-2^ | 1.32×10^-3^ | 9.91×10^-1^ | -1.60×10^-1^ | 1.83×10^-1^ |
| cg17139035 | DMR | *DDHD2* | -5.22×10^-2^ | 6.59×10^-1^ | -1.39×10^-1^ | 2.49×10^-1^ | 8.86×10^-2^ | 4.50×10^-1^ | -1.05×10^-1^ | 3.86×10^-1^ |
| cg12859234 | DMR | *DDHD2* | 6.44×10^-2^ | 5.86×10^-1^ | 1.13×10^-1^ | 3.46×10^-1^ | 6.76×10^-2^ | 5.64×10^-1^ | -1.30×10^-1^ | 2.81×10^-1^ |
| cg20338628 | DMR | *DDHD2* | 5.96×10^-2^ | 6.14×10^-1^ | 2.16×10^-2^ | 8.58×10^-1^ | -7.93×10^-2^ | 4.99×10^-1^ | -7.07×10^-2^ | 5.58×10^-1^ |
| cg16232702 | DMR | *DDHD2* | -1.39×10^-1^ | 2.38×10^-1^ | 5.51×10^-3^ | 9.64×10^-1^ | -3.00×10^-1^ | 8.86×10^-3^ | -1.31×10^-1^ | 2.75×10^-1^ |
| cg07088328 | DMR | *DDHD2* | 6.15×10^-2^ | 6.03×10^-1^ | -9.83×10^-2^ | 4.15×10^-1^ | 1.02×10^-1^ | 3.86×10^-1^ | 5.67×10^-2^ | 6.38×10^-1^ |
| cg27052402 | DMR | *DDHD2* | 1.47×10^-1^ | 2.12×10^-1^ | 1.96×10^-1^ | 1.02×10^-1^ | 1.24×10^-1^ | 2.88×10^-1^ | 2.85×10^-1^ | 1.62×10^-2^ |
| cg24002388 | DMR | *DDHD2* | -6.13×10^-3^ | 9.59×10^-1^ | 1.26×10^-1^ | 2.95×10^-1^ | -8.11×10^-2^ | 4.89×10^-1^ | 1.83×10^-1^ | 1.27×10^-1^ |
| cg11201273 | DMR | *DDHD2* | 1.67×10^-1^ | 1.55×10^-1^ | 3.70×10^-2^ | 7.60×10^-1^ | -1.15×10^-1^ | 3.26×10^-1^ | 1.69×10^-1^ | 1.59×10^-1^ |
| cg25308662 | DMR | *KCNG2* | 1.24×10^-1^ | 2.93×10^-1^ | 3.09×10^-1^ | 8.72×10^-3^ | 2.65×10^-1^ | 2.16×10^-2^ | 1.13×10^-1^ | 3.47×10^-1^ |
| cg05212510 | DMR | *KCNG2* | 4.26×10^-1^ | 1.52×10^-4^ | 5.67×10^-1^ | 2.54×10^-7^ | 3.79×10^-1^ | 7.86×10^-4^ | 2.19×10^-1^ | 6.64×10^-2^ |
| cg21467717 | DMR | *KCNG2* | 2.09×10^-2^ | 8.60×10^-1^ | 1.68×10^-1^ | 1.62×10^-1^ | 1.81×10^-1^ | 1.20×10^-1^ | 1.11×10^-1^ | 3.56×10^-1^ |
| cg18936757 | DMR | *KCNG2* | 3.61×10^-1^ | 1.58×10^-3^ | 3.93×10^-1^ | 6.94×10^-4^ | 3.61×10^-1^ | 1.47×10^-3^ | 2.45×10^-1^ | 3.91×10^-2^ |
| cg23825213 | DMR | *KCNG2* | 1.66×10^-1^ | 1.57×10^-1^ | 1.93×10^-1^ | 1.06×10^-1^ | 1.61×10^-1^ | 1.66×10^-1^ | 7.73×10^-2^ | 5.22×10^-1^ |
| cg03682112 | DMR | *KCNG2* | 2.26×10^-1^ | 5.25×10^-2^ | 1.73×10^-1^ | 1.49×10^-1^ | 2.52×10^-1^ | 2.94×10^-2^ | 1.51×10^-1^ | 2.10×10^-1^ |
| cg27264388 | DMR | *KCNG2* | 3.47×10^-1^ | 2.49×10^-3^ | 4.09×10^-1^ | 3.95×10^-4^ | 3.44×10^-1^ | 2.47×10^-3^ | 1.32×10^-1^ | 2.72×10^-1^ |
| cg24959938 | Colocalization | *KCNG2* | 1.40×10^-1^ | 2.34×10^-1^ | 2.19×10^-1^ | 6.64×10^-2^ | 1.38×10^-1^ | 2.38×10^-1^ | 5.49×10^-2^ | 6.49×10^-1^ |
| cg07082201 | Colocalization | *LINC01795* | -3.21×10^-2^ | 7.86×10^-1^ | 2.17×10^-2^ | 8.57×10^-1^ | 1.49×10^-1^ | 2.01×10^-1^ | -3.47×10^-2^ | 7.74×10^-1^ |
| cg06242242 | Colocalization | *SBNO1* | 8.67×10^-1^ | 1.65×10^-23^ | 8.55×10^-1^ | 2.45×10^-21^ | 8.32×10^-1^ | 2.47×10^-20^ | 5.95×10^-2^ | 6.22×10^-1^ |
| cg16068780 | Colocalization | *SBNO1* | 1.68×10^-1^ | 1.52×10^-1^ | 2.59×10^-1^ | 2.90×10^-2^ | 2.18×10^-1^ | 6.01×10^-2^ | 5.41×10^-2^ | 6.54×10^-1^ |

The CpG sites with blood-brain consistency in DNA methylation level, which will be utilized to construct the proxy DNA methylation model (proxyDNAm), are represented by items in red. *DMR* differentially methylated region, *PFC* prefrontal cortex, *EC* entorhinal cortex, *STG* superior temporal gyrus, *CER* cerebellum, *SEMA7A* Semaphorin 7A, *DDHD2* DDHD domain containing 2, *KCNG2* potassium voltage-gated channel modifier subfamily G member 2, *LINC01795* long intergenic non-protein coding RNA 1795, *SBNO1* strawberry notch homolog 1

1. Evaluation of methylation proxy models

| CpG | Gene | Algorithm | Resample methods | RMSE | *r* | | *P-*value | |
| --- | --- | --- | --- | --- | --- | --- | --- | --- |
|  |  |  |  |  | Training dataset | Test dataset | Training dataset | Test dataset |
| cg05964363 | *DDHD2* | QRF | 10×10-Fold CV | 1.24 | 0.46 | 0.05 | 4.94×10^-1^ | 5.71×10^-21^ |
| cg05964363 | *DDHD2* | QRF | LOOCV | 1.22 | 0.46 | 0.05 | 5.28×10^-1^ | 1.04×10^-20^ |
| cg05964363 | *DDHD2* | RF | LOOCV | 1.13 | 0.47 | 0.13 | 1.10×10^-1^ | 3.58×10^-22^ |
| cg05964363 | *DDHD2* | RF | 10×10-Fold CV | 1.12 | 0.47 | 0.13 | 1.14×10^-1^ | 1.09×10^-21^ |
| cg05964363 | *DDHD2* | SVMPoly | LOOCV | 213.24 | 0.14 | 0.01 | 8.96×10^-1^ | 6.57×10^-3^ |
| cg05964363 | *DDHD2* | SVMPoly | 10×10-Fold CV | 124.41 | 0.16 | 0.00 | 9.61×10^-1^ | 1.91×10^-3^ |
| cg16232702 | *DDHD2* | QRF | 10×10-Fold CV | 1.25 | 0.37 | 0.06 | 4.36×10^-1^ | 1.26×10^-13^ |
| cg16232702 | *DDHD2* | QRF | LOOCV | 1.22 | 0.36 | -0.02 | 8.11×10^-1^ | 1.13×10^-12^ |
| cg16232702 | *DDHD2* | RF | LOOCV | 1.15 | 0.39 | 0.07 | 3.85×10^-1^ | 2.18×10^-15^ |
| cg16232702 | *DDHD2* | RF | 10×10-Fold CV | 1.18 | 0.40 | 0.07 | 3.90×10^-1^ | 3.50×10^-16^ |
| cg16232702 | *DDHD2* | SVMPoly | 10×10-Fold CV | 246.63 | 0.10 | 0.12 | 1.39×10^-1^ | 5.23×10^-2^ |
| cg16232702 | *DDHD2* | SVMPoly | LOOCV | 121.37 | 0.15 | 0.07 | 4.11×10^-1^ | 4.36×10^-3^ |
| cg16937126 | *DDHD2* | QRF | 10×10-Fold CV | 1.24 | 0.47 | 0.16 | 4.18×10^-2^ | 3.22×10^-22^ |
| cg16937126 | *DDHD2* | QRF | LOOCV | 1.23 | 0.46 | 0.13 | 1.08×10^-1^ | 3.06×10^-21^ |
| cg16937126 | *DDHD2* | RF | 10×10-Fold CV | 1.14 | 0.45 | 0.23 | 4.08×10^-3^ | 8.94×10^-20^ |
| cg16937126 | *DDHD2* | RF | LOOCV | 1.15 | 0.44 | 0.22 | 5.88×10^-3^ | 4.87×10^-19^ |
| cg16937126 | *DDHD2* | SVMPoly | 10×10-Fold CV | 86.04 | 0.21 | 0.27 | 7.94×10^-4^ | 6.15×10^-5^ |
| cg16937126 | *DDHD2* | SVMPoly | LOOCV | 114.93 | 0.20 | 0.26 | 9.02×10^-4^ | 1.20×10^-4^ |
| cg27052402 | *DDHD2* | QRF | 10×10-Fold CV | 1.25 | 0.48 | 0.02 | 7.61×10^-1^ | 1.28×10^-22^ |
| cg27052402 | *DDHD2* | QRF | LOOCV | 1.24 | 0.43 | 0.02 | 8.39×10^-1^ | 2.14×10^-18^ |
| cg27052402 | *DDHD2* | RF | 10×10-Fold CV | 1.14 | 0.43 | 0.05 | 5.42×10^-1^ | 4.09×10^-18^ |
| cg27052402 | *DDHD2* | RF | LOOCV | 1.15 | 0.43 | 0.04 | 6.15×10^-1^ | 2.91×10^-18^ |
| cg27052402 | *DDHD2* | SVMPoly | 10×10-Fold CV | 308.09 | 0.20 | 0.08 | 3.12×10^-1^ | 6.80×10^-5^ |
| cg27052402 | *DDHD2* | SVMPoly | LOOCV | 205.27 | 0.22 | 0.05 | 5.01×10^-1^ | 2.26×10^-5^ |
| cg03682112 | *KCNG2* | QRF | 10×10-Fold CV | 1.08 | 0.07 | 0.13 | 1.01×10^-1^ | 1.80×10^-1^ |
| cg03682112 | *KCNG2* | QRF | LOOCV | 1.10 | 0.08 | 0.13 | 1.03×10^-1^ | 1.06×10^-1^ |
| cg03682112 | *KCNG2* | RF | 10×10-Fold CV | 1.19 | 0.50 | 0.13 | 9.67×10^-2^ | 8.14×10^-25^ |
| cg03682112 | *KCNG2* | RF | LOOCV | 1.15 | 0.52 | 0.10 | 1.92×10^-1^ | 3.03×10^-27^ |
| cg03682112 | *KCNG2* | SVMPoly | LOOCV | 434.39 | 0.07 | 0.08 | 3.17×10^-1^ | 2.06×10^-1^ |
| cg03682112 | *KCNG2* | SVMPoly | 10×10-Fold CV | 456.45 | 0.06 | 0.07 | 3.51×10^-1^ | 2.12×10^-1^ |
| cg05212510 | *KCNG2* | QRF | LOOCV | 0.64 | 0.00 | 0.04 | 5.77×10^-1^ | 9.47×10^-1^ |
| cg05212510 | *KCNG2* | QRF | 10×10-Fold CV | 0.66 | 0.01 | -0.02 | 7.60×10^-1^ | 8.68×10^-1^ |
| cg05212510 | *KCNG2* | RF | LOOCV | 1.02 | 0.28 | 0.07 | 4.01×10^-1^ | 2.16×10^-8^ |
| cg05212510 | *KCNG2* | RF | 10×10-Fold CV | 0.96 | 0.28 | 0.06 | 4.70×10^-1^ | 2.94×10^-8^ |
| cg05212510 | *KCNG2* | SVMPoly | 10×10-Fold CV | 239.07 | 0.07 | 0.07 | 3.95×10^-1^ | 1.54×10^-1^ |
| cg05212510 | *KCNG2* | SVMPoly | LOOCV | 422.13 | 0.06 | 0.04 | 6.43×10^-1^ | 2.38×10^-1^ |
| cg18936757 | *KCNG2* | QRF | 10×10-Fold CV | 1.19 | 0.16 | 0.01 | 9.26×10^-1^ | 1.39×10^-3^ |
| cg18936757 | *KCNG2* | QRF | LOOCV | 1.25 | 0.15 | -0.01 | 9.36×10^-1^ | 2.76×10^-3^ |
| cg18936757 | *KCNG2* | RF | 10×10-Fold CV | 1.18 | 0.42 | -0.11 | 1.57×10^-1^ | 1.15×10^-17^ |
| cg18936757 | *KCNG2* | RF | LOOCV | 1.16 | 0.41 | -0.11 | 1.72×10^-1^ | 7.39×10^-17^ |
| cg18936757 | *KCNG2* | SVMPoly | LOOCV | 734.57 | 0.15 | -0.05 | 5.31×10^-1^ | 2.76×10^-3^ |
| cg18936757 | *KCNG2* | SVMPoly | 10×10-Fold CV | 249.59 | 0.16 | -0.03 | 6.71×10^-1^ | 2.38×10^-3^ |
| cg25308662 | *KCNG2* | QRF | LOOCV | 1.24 | 0.31 | -0.03 | 7.44×10^-1^ | 8.02×10^-10^ |
| cg25308662 | *KCNG2* | QRF | 10×10-Fold CV | 1.15 | 0.34 | 0.01 | 9.06×10^-1^ | 2.51×10^-11^ |
| cg25308662 | *KCNG2* | RF | LOOCV | 1.14 | 0.41 | -0.05 | 5.53×10^-1^ | 2.97×10^-16^ |
| cg25308662 | *KCNG2* | RF | 10×10-Fold CV | 1.14 | 0.41 | -0.05 | 5.76×10^-1^ | 1.92×10^-16^ |
| cg25308662 | *KCNG2* | SVMPoly | LOOCV | 869.70 | 0.24 | 0.00 | 9.56×10^-1^ | 2.35×10^-6^ |
| cg25308662 | *KCNG2* | SVMPoly | 10×10-Fold CV | 327.33 | 0.24 | 0.00 | 9.85×10^-1^ | 2.10×10^-6^ |
| cg27264388 | *KCNG2* | QRF | LOOCV | 1.16 | 0.15 | 0.02 | 7.91×10^-1^ | 4.63×10^-3^ |
| cg27264388 | *KCNG2* | QRF | 10×10-Fold CV | 1.17 | 0.05 | 0.02 | 8.42×10^-1^ | 3.65×10^-1^ |
| cg27264388 | *KCNG2* | RF | LOOCV | 1.17 | 0.45 | 0.03 | 6.86×10^-1^ | 2.87×10^-20^ |
| cg27264388 | *KCNG2* | RF | 10×10-Fold CV | 1.03 | 0.45 | 0.03 | 7.08×10^-1^ | 5.31×10^-20^ |
| cg27264388 | *KCNG2* | SVMPoly | LOOCV | 377.81 | 0.09 | 0.10 | 1.95×10^-1^ | 9.23×10^-2^ |
| cg27264388 | *KCNG2* | SVMPoly | 10×10-Fold CV | 78.82 | 0.08 | 0.08 | 3.11×10^-1^ | 1.08×10^-1^ |
| cg00080972 | *RUFY1* | QRF | LOOCV | 1.17 | 0.49 | 0.49 | 5.67×10^-11^ | 1.76×10^-24^ |
| cg00080972 | *RUFY1* | QRF | 10×10-Fold CV | 1.17 | 0.50 | 0.48 | 1.32×10^-10^ | 3.83×10^-25^ |
| cg00080972 | *RUFY1* | RF | LOOCV | 1.09 | 0.48 | 0.51 | 6.77×10^-12^ | 1.14×10^-22^ |
| cg00080972 | *RUFY1* | RF | 10×10-Fold CV | 1.09 | 0.48 | 0.51 | 8.44×10^-12^ | 1.05×10^-22^ |
| cg00080972 | *RUFY1* | SVMPoly | 10×10-Fold CV | 163.69 | 0.41 | 0.49 | 4.78×10^-11^ | 6.71×10^-17^ |
| cg00080972 | *RUFY1* | SVMPoly | LOOCV | 56.03 | 0.42 | 0.47 | 3.75×10^-10^ | 1.01×10^-17^ |
| cg01955137 | *RUFY1* | QRF | 10×10-Fold CV | 1.23 | 0.48 | 0.38 | 1.10×10^-6^ | 6.42×10^-23^ |
| cg01955137 | *RUFY1* | QRF | LOOCV | 1.19 | 0.47 | 0.34 | 9.83×10^-6^ | 8.23×10^-22^ |
| cg01955137 | *RUFY1* | RF | 10×10-Fold CV | 1.11 | 0.44 | 0.38 | 7.03×10^-7^ | 1.06×10^-18^ |
| cg01955137 | *RUFY1* | RF | LOOCV | 1.10 | 0.44 | 0.38 | 7.82×10^-7^ | 6.86×10^-19^ |
| cg01955137 | *RUFY1* | SVMPoly | LOOCV | 89.35 | 0.35 | 0.37 | 2.44×10^-6^ | 2.68×10^-12^ |
| cg01955137 | *RUFY1* | SVMPoly | 10×10-Fold CV | 101.78 | 0.37 | 0.34 | 1.14×10^-5^ | 6.85×10^-14^ |
| cg02136620 | *RUFY1* | QRF | 10×10-Fold CV | 1.23 | 0.46 | 0.36 | 2.98×10^-6^ | 3.45×10^-21^ |
| cg02136620 | *RUFY1* | QRF | LOOCV | 1.25 | 0.46 | 0.35 | 7.13×10^-6^ | 5.07×10^-21^ |
| cg02136620 | *RUFY1* | RF | LOOCV | 1.12 | 0.43 | 0.39 | 3.74×10^-7^ | 3.20×10^-18^ |
| cg02136620 | *RUFY1* | RF | 10×10-Fold CV | 1.11 | 0.43 | 0.39 | 3.86×10^-7^ | 3.82×10^-18^ |
| cg02136620 | *RUFY1* | SVMPoly | 10×10-Fold CV | 370.24 | 0.37 | 0.37 | 2.38×10^-6^ | 2.83×10^-13^ |
| cg02136620 | *RUFY1* | SVMPoly | LOOCV | 231.93 | 0.37 | 0.36 | 3.99×10^-6^ | 3.01×10^-13^ |
| cg05457628 | *RUFY1* | QRF | LOOCV | 1.20 | 0.51 | 0.48 | 1.70×10^-10^ | 2.21×10^-26^ |
| cg05457628 | *RUFY1* | QRF | 10×10-Fold CV | 1.14 | 0.51 | 0.46 | 1.53×10^-9^ | 3.77×10^-26^ |
| cg05457628 | *RUFY1* | RF | 10×10-Fold CV | 1.09 | 0.48 | 0.53 | 5.67×10^-13^ | 1.23×10^-22^ |
| cg05457628 | *RUFY1* | RF | LOOCV | 1.11 | 0.47 | 0.53 | 7.11×10^-13^ | 3.10×10^-22^ |
| cg05457628 | *RUFY1* | SVMPoly | LOOCV | 105.48 | 0.40 | 0.50 | 2.40×10^-11^ | 5.53×10^-16^ |
| cg05457628 | *RUFY1* | SVMPoly | 10×10-Fold CV | 118.05 | 0.40 | 0.48 | 2.18×10^-10^ | 6.98×10^-16^ |
| cg06118287 | *RUFY1* | QRF | 10×10-Fold CV | 1.15 | 0.52 | 0.55 | 8.37×10^-14^ | 3.20×10^-27^ |
| cg06118287 | *RUFY1* | QRF | LOOCV | 1.13 | 0.51 | 0.54 | 1.84×10^-13^ | 4.34×10^-26^ |
| cg06118287 | *RUFY1* | RF | LOOCV | 1.07 | 0.50 | 0.55 | 4.77×10^-14^ | 5.95×10^-25^ |
| cg06118287 | *RUFY1* | RF | 10×10-Fold CV | 1.07 | 0.50 | 0.55 | 5.89×10^-14^ | 3.38×10^-25^ |
| cg06118287 | *RUFY1* | SVMPoly | LOOCV | 238.50 | 0.47 | 0.56 | 2.16×10^-14^ | 3.92×10^-22^ |
| cg06118287 | *RUFY1* | SVMPoly | 10×10-Fold CV | 94.41 | 0.45 | 0.54 | 3.25×10^-13^ | 3.91×10^-20^ |
| cg08058472 | *RUFY1* | QRF | LOOCV | 1.18 | 0.51 | 0.52 | 2.23×10^-12^ | 4.79×10^-26^ |
| cg08058472 | *RUFY1* | QRF | 10×10-Fold CV | 1.15 | 0.50 | 0.51 | 5.84×10^-12^ | 8.28×10^-25^ |
| cg08058472 | *RUFY1* | RF | LOOCV | 1.07 | 0.48 | 0.56 | 3.10×10^-14^ | 6.20×10^-23^ |
| cg08058472 | *RUFY1* | RF | 10×10-Fold CV | 1.09 | 0.48 | 0.56 | 3.82×10^-14^ | 8.58×10^-23^ |
| cg08058472 | *RUFY1* | SVMPoly | LOOCV | 248.33 | 0.42 | 0.54 | 4.38×10^-13^ | 4.30×10^-17^ |
| cg08058472 | *RUFY1* | SVMPoly | 10×10-Fold CV | 101.18 | 0.41 | 0.52 | 4.60×10^-12^ | 7.39×10^-17^ |
| cg09060608 | *RUFY1* | QRF | LOOCV | 1.19 | 0.48 | 0.52 | 2.55×10^-12^ | 6.98×10^-23^ |
| cg09060608 | *RUFY1* | QRF | 10×10-Fold CV | 1.15 | 0.48 | 0.51 | 1.11×10^-11^ | 1.19×10^-22^ |
| cg09060608 | *RUFY1* | RF | LOOCV | 1.09 | 0.48 | 0.55 | 7.99×10^-14^ | 1.12×10^-22^ |
| cg09060608 | *RUFY1* | RF | 10×10-Fold CV | 1.10 | 0.48 | 0.55 | 8.03×10^-14^ | 6.51×10^-23^ |
| cg09060608 | *RUFY1* | SVMPoly | LOOCV | 280.34 | 0.43 | 0.56 | 3.84×10^-14^ | 4.52×10^-18^ |
| cg09060608 | *RUFY1* | SVMPoly | 10×10-Fold CV | 63.70 | 0.44 | 0.48 | 2.14×10^-10^ | 2.23×10^-19^ |
| cg14820908 | *RUFY1* | QRF | LOOCV | 1.17 | 0.51 | 0.50 | 1.63×10^-11^ | 5.04×10^-26^ |
| cg14820908 | *RUFY1* | QRF | 10×10-Fold CV | 1.16 | 0.49 | 0.50 | 2.97×10^-11^ | 6.06×10^-24^ |
| cg14820908 | *RUFY1* | RF | LOOCV | 1.05 | 0.50 | 0.51 | 1.32×10^-11^ | 4.37×10^-25^ |
| cg14820908 | *RUFY1* | RF | 10×10-Fold CV | 1.08 | 0.50 | 0.50 | 1.85×10^-11^ | 8.28×10^-25^ |
| cg14820908 | *RUFY1* | SVMPoly | LOOCV | 374.46 | 0.44 | 0.47 | 6.68×10^-10^ | 8.56×10^-19^ |
| cg14820908 | *RUFY1* | SVMPoly | 10×10-Fold CV | 120.14 | 0.44 | 0.46 | 2.07×10^-9^ | 9.84×10^-19^ |
| cg20199595 | *RUFY1* | QRF | 10×10-Fold CV | 1.14 | 0.53 | 0.54 | 1.99×10^-13^ | 9.32×10^-29^ |
| cg20199595 | *RUFY1* | QRF | LOOCV | 1.15 | 0.53 | 0.52 | 1.92×10^-12^ | 6.15×10^-28^ |
| cg20199595 | *RUFY1* | RF | LOOCV | 1.08 | 0.49 | 0.54 | 3.06×10^-13^ | 4.53×10^-24^ |
| cg20199595 | *RUFY1* | RF | 10×10-Fold CV | 1.10 | 0.49 | 0.54 | 3.11×10^-13^ | 1.83×10^-24^ |
| cg20199595 | *RUFY1* | SVMPoly | LOOCV | 281.52 | 0.44 | 0.51 | 7.73×10^-12^ | 1.64×10^-19^ |
| cg20199595 | *RUFY1* | SVMPoly | 10×10-Fold CV | 137.44 | 0.42 | 0.46 | 1.13×10^-9^ | 1.00×10^-17^ |
| cg21226059 | *RUFY1* | QRF | LOOCV | 1.12 | 0.51 | 0.54 | 1.76×10^-13^ | 3.15×10^-26^ |
| cg21226059 | *RUFY1* | QRF | 10×10-Fold CV | 1.14 | 0.51 | 0.54 | 3.88×10^-13^ | 2.34×10^-26^ |
| cg21226059 | *RUFY1* | RF | LOOCV | 1.05 | 0.49 | 0.54 | 1.91×10^-13^ | 3.35×10^-24^ |
| cg21226059 | *RUFY1* | RF | 10×10-Fold CV | 1.07 | 0.49 | 0.54 | 2.47×10^-13^ | 1.97×10^-24^ |
| cg21226059 | *RUFY1* | SVMPoly | LOOCV | 300.33 | 0.45 | 0.54 | 3.02×10^-13^ | 4.77×10^-20^ |
| cg21226059 | *RUFY1* | SVMPoly | 10×10-Fold CV | 170.66 | 0.43 | 0.48 | 1.75×10^-10^ | 2.01×10^-18^ |
| cg22764044 | *RUFY1* | QRF | LOOCV | 1.20 | 0.49 | 0.43 | 1.37×10^-8^ | 2.99×10^-24^ |
| cg22764044 | *RUFY1* | QRF | 10×10-Fold CV | 1.18 | 0.49 | 0.42 | 3.34×10^-8^ | 5.50×10^-24^ |
| cg22764044 | *RUFY1* | RF | 10×10-Fold CV | 1.09 | 0.46 | 0.46 | 1.37×10^-9^ | 9.48×10^-21^ |
| cg22764044 | *RUFY1* | RF | LOOCV | 1.09 | 0.46 | 0.46 | 1.64×10^-9^ | 6.17×10^-21^ |
| cg22764044 | *RUFY1* | SVMPoly | LOOCV | 157.27 | 0.39 | 0.46 | 1.46×10^-9^ | 2.94×10^-15^ |
| cg22764044 | *RUFY1* | SVMPoly | 10×10-Fold CV | 90.43 | 0.40 | 0.42 | 3.89×10^-8^ | 2.00×10^-15^ |
| cg25658438 | *RUFY1* | QRF | LOOCV | 1.17 | 0.50 | 0.56 | 1.77×10^-14^ | 1.97×10^-25^ |
| cg25658438 | *RUFY1* | QRF | 10×10-Fold CV | 1.18 | 0.49 | 0.56 | 1.84×10^-14^ | 1.64×10^-24^ |
| cg25658438 | *RUFY1* | RF | 10×10-Fold CV | 1.09 | 0.50 | 0.55 | 1.21×10^-13^ | 1.17×10^-24^ |
| cg25658438 | *RUFY1* | RF | LOOCV | 1.08 | 0.50 | 0.55 | 1.23×10^-13^ | 1.01×10^-24^ |
| cg25658438 | *RUFY1* | SVMPoly | LOOCV | 105.02 | 0.43 | 0.47 | 5.74×10^-10^ | 3.00×10^-18^ |
| cg25658438 | *RUFY1* | SVMPoly | 10×10-Fold CV | 184.28 | 0.43 | 0.47 | 8.18×10^-10^ | 4.19×10^-18^ |
| cg26516362 | *RUFY1* | QRF | LOOCV | 1.18 | 0.51 | 0.46 | 1.50×10^-9^ | 1.04×10^-25^ |
| cg26516362 | *RUFY1* | QRF | 10×10-Fold CV | 1.17 | 0.51 | 0.45 | 2.71×10^-9^ | 1.08×10^-26^ |
| cg26516362 | *RUFY1* | RF | 10×10-Fold CV | 1.10 | 0.47 | 0.47 | 4.07×10^-10^ | 3.83×10^-22^ |
| cg26516362 | *RUFY1* | RF | LOOCV | 1.09 | 0.47 | 0.47 | 4.41×10^-10^ | 3.04×10^-22^ |
| cg26516362 | *RUFY1* | SVMPoly | LOOCV | 212.77 | 0.41 | 0.43 | 1.31×10^-8^ | 8.33×10^-17^ |
| cg26516362 | *RUFY1* | SVMPoly | 10×10-Fold CV | 111.77 | 0.41 | 0.43 | 2.18×10^-8^ | 6.93×10^-17^ |
| cg06242242 | *SBNO1* | QRF | 10×10-Fold CV | 1.32 | 0.38 | -0.09 | 2.84×10^-1^ | 1.80×10^-14^ |
| cg06242242 | *SBNO1* | QRF | LOOCV | 1.31 | 0.38 | -0.05 | 5.18×10^-1^ | 2.39×10^-14^ |
| cg06242242 | *SBNO1* | RF | LOOCV | 1.17 | 0.43 | -0.03 | 7.05×10^-1^ | 1.36×10^-18^ |
| cg06242242 | *SBNO1* | RF | 10×10-Fold CV | 1.16 | 0.43 | -0.02 | 8.11×10^-1^ | 1.28×10^-18^ |
| cg06242242 | *SBNO1* | SVMPoly | LOOCV | 89.78 | 0.23 | -0.04 | 6.39×10^-1^ | 8.33×10^-6^ |
| cg06242242 | *SBNO1* | SVMPoly | 10×10-Fold CV | 104.44 | 0.17 | -0.02 | 8.09×10^-1^ | 9.27×10^-4^ |
| cg16068780 | *SBNO1* | QRF | LOOCV | 0.89 | 0.26 | 0.12 | 1.39×10^-1^ | 2.40×10^-7^ |
| cg16068780 | *SBNO1* | QRF | 10×10-Fold CV | 1.29 | 0.12 | 0.12 | 1.40×10^-1^ | 1.84×10^-2^ |
| cg16068780 | *SBNO1* | RF | 10×10-Fold CV | 1.24 | 0.56 | 0.70 | 1.42×10^-24^ | 9.11×10^-32^ |
| cg16068780 | *SBNO1* | RF | LOOCV | 1.27 | 0.55 | 0.68 | 7.13×10^-23^ | 6.01×10^-31^ |
| cg16068780 | *SBNO1* | SVMPoly | LOOCV | 369.20 | 0.18 | 0.20 | 1.11×10^-2^ | 4.06×10^-4^ |
| cg16068780 | *SBNO1* | SVMPoly | 10×10-Fold CV | 476.11 | 0.18 | 0.18 | 2.51×10^-2^ | 5.70×10^-4^ |
| cg06263834 | *SEMA7A* | QRF | 10×10-Fold CV | 1.22 | 0.38 | 0.07 | 3.85×10^-1^ | 1.22×10^-14^ |
| cg06263834 | *SEMA7A* | QRF | LOOCV | 1.25 | 0.39 | 0.02 | 8.20×10^-1^ | 1.10×10^-14^ |
| cg06263834 | *SEMA7A* | RF | 10×10-Fold CV | 1.14 | 0.46 | 0.11 | 1.57×10^-1^ | 6.87×10^-21^ |
| cg06263834 | *SEMA7A* | RF | LOOCV | 1.14 | 0.47 | 0.11 | 1.70×10^-1^ | 1.66×10^-21^ |
| cg06263834 | *SEMA7A* | SVMPoly | LOOCV | 79.64 | 0.20 | 0.00 | 9.77×10^-1^ | 9.35×10^-5^ |
| cg06263834 | *SEMA7A* | SVMPoly | 10×10-Fold CV | 77.18 | 0.24 | 0.00 | 9.81×10^-1^ | 2.48×10^-6^ |
| cg08125401 | *SEMA7A* | QRF | LOOCV | 1.28 | 0.41 | -0.07 | 3.70×10^-1^ | 6.71×10^-17^ |
| cg08125401 | *SEMA7A* | QRF | 10×10-Fold CV | 1.27 | 0.38 | -0.05 | 5.58×10^-1^ | 4.17×10^-14^ |
| cg08125401 | *SEMA7A* | RF | 10×10-Fold CV | 1.19 | 0.41 | -0.07 | 3.75×10^-1^ | 1.97×10^-16^ |
| cg08125401 | *SEMA7A* | RF | LOOCV | 1.17 | 0.40 | -0.06 | 4.89×10^-1^ | 7.32×10^-16^ |
| cg08125401 | *SEMA7A* | SVMPoly | LOOCV | 47.28 | 0.23 | -0.01 | 9.19×10^-1^ | 1.06×10^-5^ |
| cg08125401 | *SEMA7A* | SVMPoly | 10×10-Fold CV | 107.11 | 0.21 | 0.00 | 9.70×10^-1^ | 3.02×10^-5^ |
| cg20736847 | *SEMA7A* | QRF | 10×10-Fold CV | 1.25 | 0.38 | 0.07 | 3.53×10^-1^ | 2.10×10^-14^ |
| cg20736847 | *SEMA7A* | QRF | LOOCV | 1.21 | 0.39 | 0.06 | 4.70×10^-1^ | 3.74×10^-15^ |
| cg20736847 | *SEMA7A* | RF | LOOCV | 1.17 | 0.43 | 0.12 | 1.51×10^-1^ | 7.17×10^-18^ |
| cg20736847 | *SEMA7A* | RF | 10×10-Fold CV | 1.12 | 0.43 | 0.10 | 2.08×10^-1^ | 1.71×10^-18^ |
| cg20736847 | *SEMA7A* | SVMPoly | 10×10-Fold CV | 79.17 | 0.26 | 0.16 | 4.18×10^-2^ | 3.81×10^-7^ |
| cg20736847 | *SEMA7A* | SVMPoly | LOOCV | 104.61 | 0.27 | 0.14 | 7.89×10^-2^ | 1.07×10^-7^ |
| cg20927731 | *SEMA7A* | QRF | 10×10-Fold CV | 1.24 | 0.39 | -0.06 | 4.67×10^-1^ | 5.02×10^-15^ |
| cg20927731 | *SEMA7A* | QRF | LOOCV | 1.29 | 0.39 | -0.04 | 6.26×10^-1^ | 5.59×10^-15^ |
| cg20927731 | *SEMA7A* | RF | 10×10-Fold CV | 1.16 | 0.43 | -0.08 | 3.48×10^-1^ | 6.82×10^-18^ |
| cg20927731 | *SEMA7A* | RF | LOOCV | 1.16 | 0.42 | -0.06 | 4.24×10^-1^ | 2.91×10^-17^ |
| cg20927731 | *SEMA7A* | SVMPoly | LOOCV | 177.65 | 0.25 | -0.01 | 8.55×10^-1^ | 1.41×10^-6^ |
| cg20927731 | *SEMA7A* | SVMPoly | 10×10-Fold CV | 58.57 | 0.25 | -0.01 | 8.65×10^-1^ | 1.49×10^-6^ |

The final adopted proxy methylation model for each CpG site is marked in red. *QRF* quantile random forest, *RF* random forest, *SVMPoly* support vector machines with polynomial kernel, *LOOCV* leave one out cross-validation, *10×10-Fold CV* 10-time repeated 10-fold cross-validation, *SEMA7A* Semaphorin 7A, *DDHD2* DDHD domain containing 2, *KCNG2* potassium voltage-gated channel modifier subfamily G member 2, *LINC01795* long intergenic non-protein coding RNA 1795, *SBNO1* strawberry notch homolog, *RMSE* root mean square error

1. Performance evaluation for RES-prediction models

| Cohort | Model name | *P-*value | *r* (95%CI) | AUC (95%CI) | MAE | MAPE | RMSE | *R*^2^ |
| --- | --- | --- | --- | --- | --- | --- | --- | --- |
| Discovery cohort (*n*=2,307) | C+PGM | 0.0000 | 0.76(0.74 to 0.78) | 0.87(0.87 to 0.88) | 0.21 | 1.33 | 0.27 | 0.48 |
|  | C+P | 6.93×10^-211^ | 0.58(0.56 to 0.61) | 0.83(0.82 to 0.83) | 0.25 | 1.87 | 0.32 | 0.27 |
|  | C+M | 6.28×10^-136^ | 0.48(0.45 to 0.51) | 0.79(0.78 to 0.8) | 0.28 | 1.74 | 0.35 | 0.11 |
|  | C+G | 1.34×10^-126^ | 0.47(0.44 to 0.50) | 0.77(0.76 to 0.78) | 0.28 | 2.29 | 0.36 | 0.10 |
| Validation cohort (*n*=1,379) | C+PGM | 1.65×10^-245^ | 0.75(0.72 to 0.77) | 0.85(0.84 to 0.86) | 0.15 | 1.47 | 0.21 | 0.51 |
|  | C+P | 1.10×10^-94^ | 0.52(0.48 to 0.55) | 0.78(0.77 to 0.8) | 0.20 | 1.94 | 0.26 | 0.23 |
|  | C+M | 4.79×10^-68^ | 0.45(0.4 to 0.49) | 0.75(0.74 to 0.77) | 0.22 | 1.99 | 0.27 | 0.13 |
|  | C+G | 1.16×10^-75^ | 0.47(0.42 to 0.51) | 0.75(0.73 to 0.76) | 0.22 | 1.86 | 0.28 | 0.11 |

*C+P* clinical information +PRSs, *C+G* clinical information + GRS, *C+M* clinical information + proxyDNAm, *C+PGM* clinical information + PRS +GRS + proxyDNAm, *AUC* area under the curve, *MAE* mean absolute error, *MAPE* mean absolute percentage error, *RMSE* root mean square error, *R^2^* coefficient of determination

# Figures


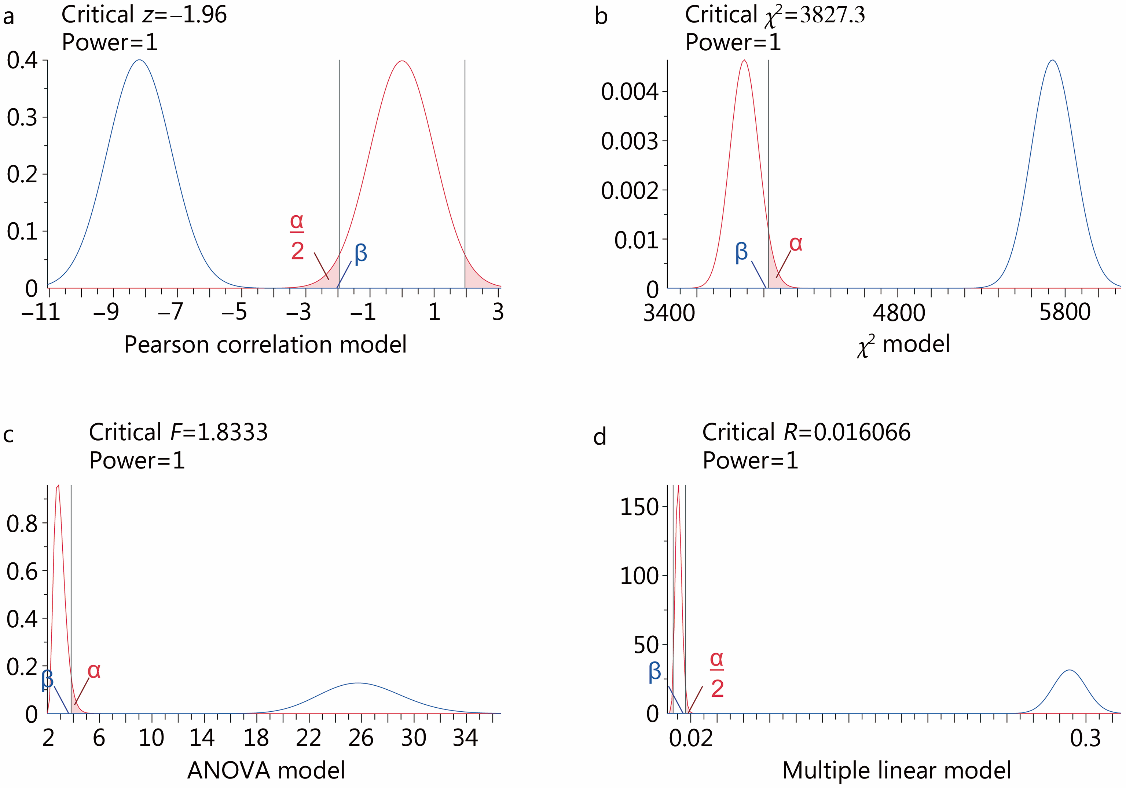


1. Statistical power of samples. The statistical power of a sample size of 3686 was calculated using G*power software. **a** In Pearson correlation model, when the error probability of α was 0.05, the power (1-error probability of β) was 1. **b** In *χ*^2^ correlation model, when the error probability of α was 0.05 and the ratio of variable 1 and variable 2 was 1.5, the power (1-error probability of β) was 1. **c** In ANOVA model, when the effect size of *F* was 0.25, error probability of α was 0.05, and number of groups was 7 (same as the number of APDs type), the power (1-error probability of β) was 1. **d** In the multiple-linear model, the power (1-error probability of β) was 1 with parameters that error probability of α was 0.05, and number of predictors was 40. ANOVA analysis of variance, APD antipsychotic drug


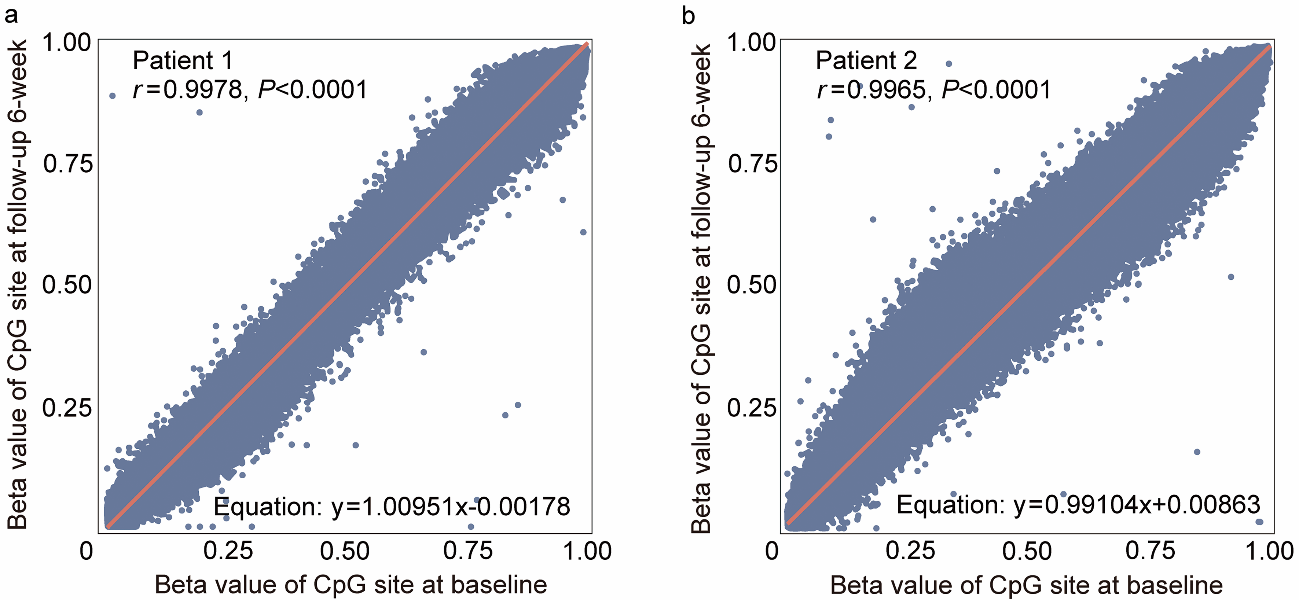


1. Influence of APDs treatment on DNAm and technical replication. Two patients (sample 1 and sample 2) were randomly selected to profile and compare their genome-wide methylation at baseline and after 6 weeks of antipsychotic drug treatment. The correlation between the two time points was analyzed using Pearson correlation. **a-b** Both samples showed significant correlation (*P* < 0.0001) between baseline and at 6-week with antipsychotic drug treatment. Orange line represented the linear regression line. APDs antipsychotic drugs


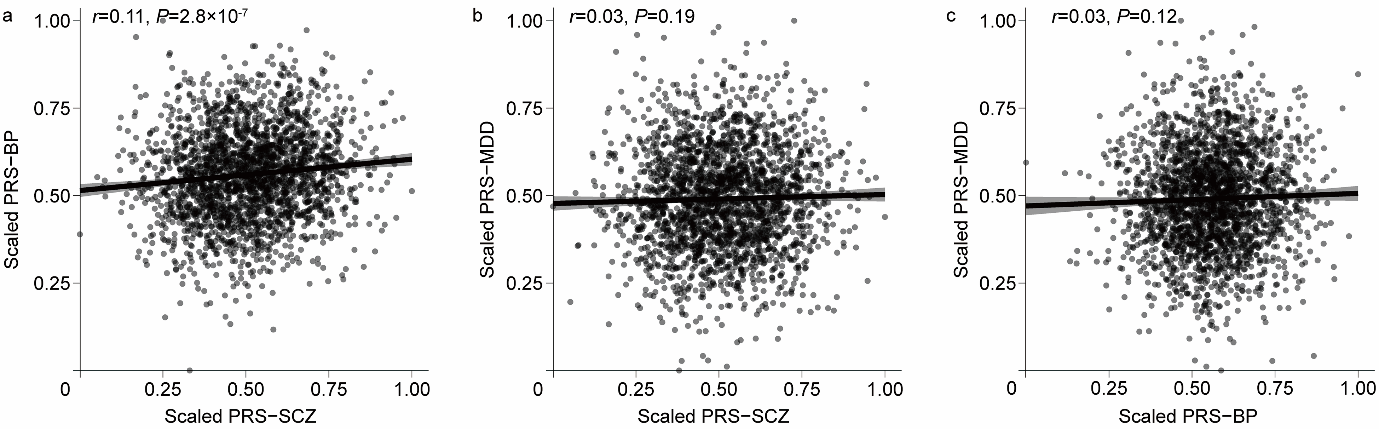


1. Correlation between PRSs. Pearson correlation suggested the PRS-SCZ was significantly correlated to PRS-BP (**a**) but not PRS-MDD (**b**). **c** No significant correlation was observed between PRS-BP and PRS-MDD. PRS polygenic risk score, SCZ schizophrenia, BP bipolar disorder, MDD major depressive disorder


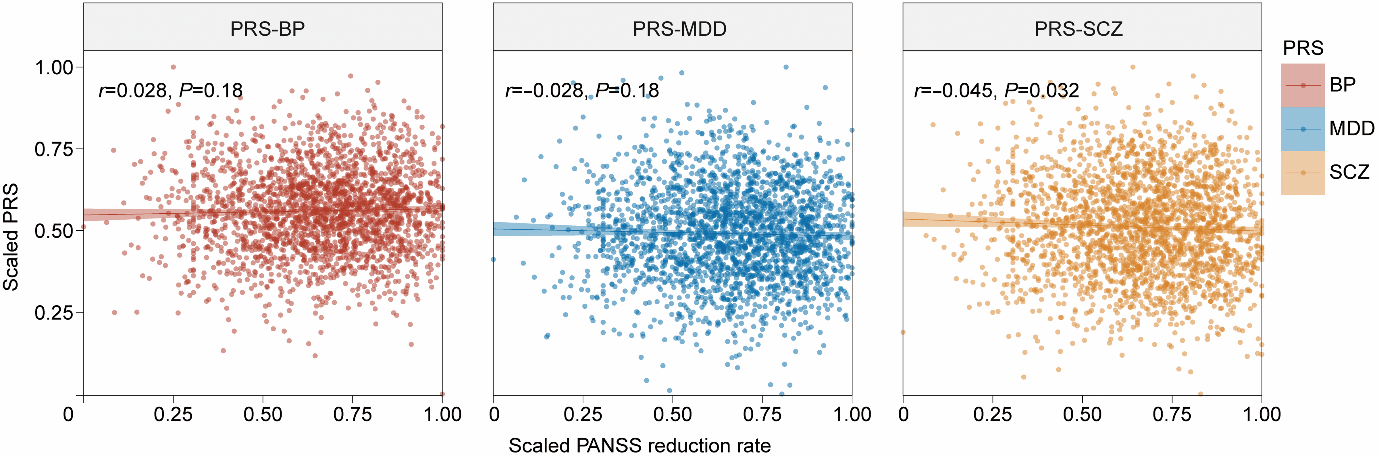


1. Relationship between treatment response and PRSs. PANSS reduction rate was significantly correlated to PRS-SCZ (*r* = -0.045, *P* = 0.032) but not PRS-BP or PRS-MDD. PANSS Positive and Negative syndrome scale, PRS polygenic risk score*,* SCZ schizophrenia, BP bipolar disorder, MDD major depressive disorder


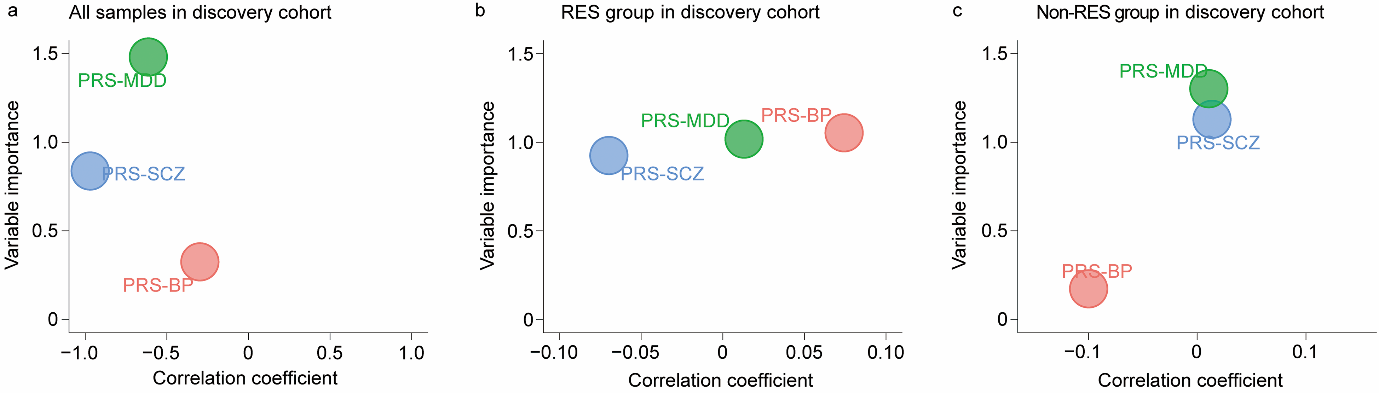


1. Variable importance plot of PRSs. A partial least square analysis was conducted to determine the importance of variables in explaining the outcome. The plot illustrated the importance of PRS-SCZ, PRS-BP, and PRS-MDD in explaining variance of treatment response and the coefficient of their correlation to treatment response in **a** all samples, **b** RES group, and **c** non-RES group, respectively. PRS polygenic risk score, SCZ schizophrenia, BP bipolar disorder, MDD major depressive disorder, RES response


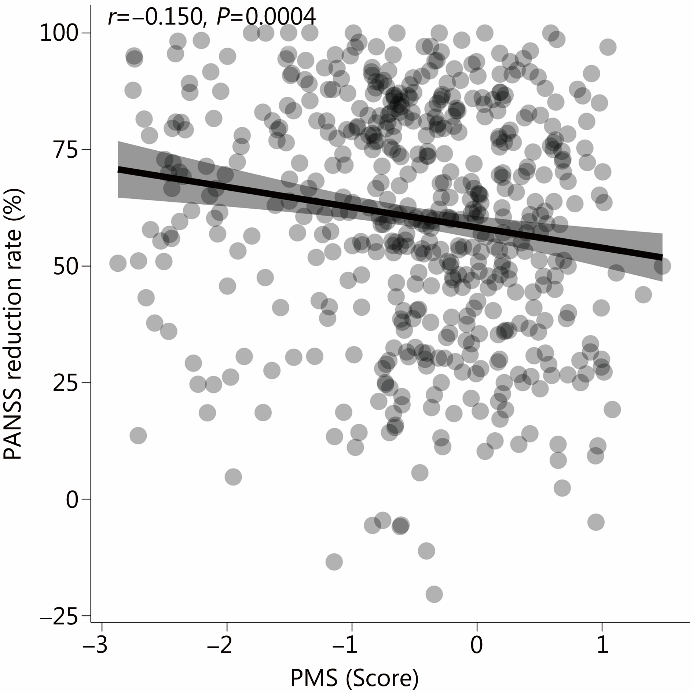


1. Correlation of PMS to PANSS reduction rate. PMS was significantly correlated to PANSS reduction rate (Pearson correlation, *r* = -0.15, *P* = 0.0004). PANSS positive and negative syndrome scale, PMS polymethylation score


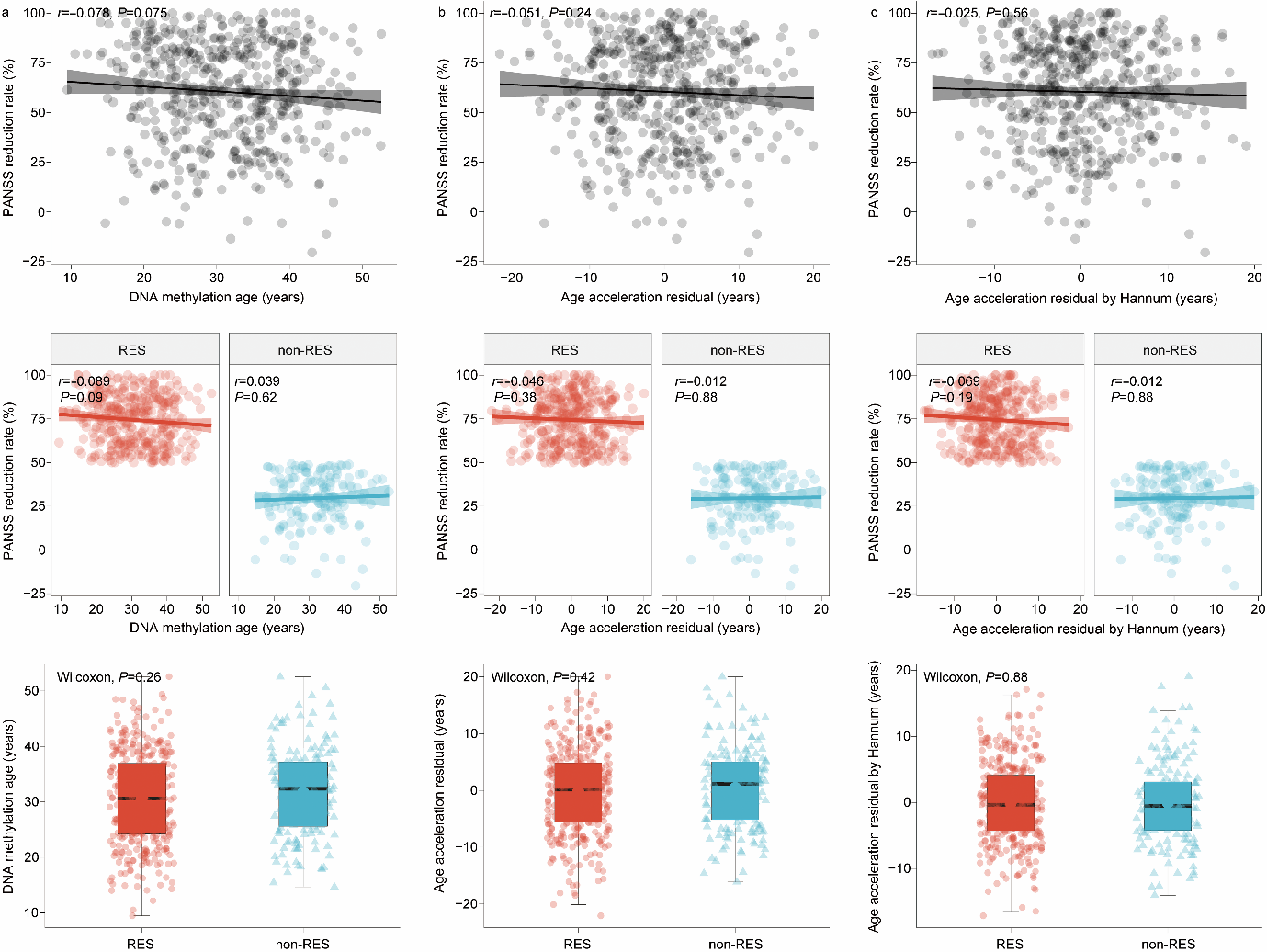


1. Correlation of epigenetic clocks and PANSS reduction rate was evaluated, which showed (**a-c)** that epigenetic clocks were not significantly (Pearson correlation, *P* > 0.05) correlated to PANSS reduction rate and that the variance of epigenetic clocks was not significantly different between RES group and non-RES group (Wilcoxon test, *P* > 0.05). PANSS positive and negative syndrome scale, RES response

**C**


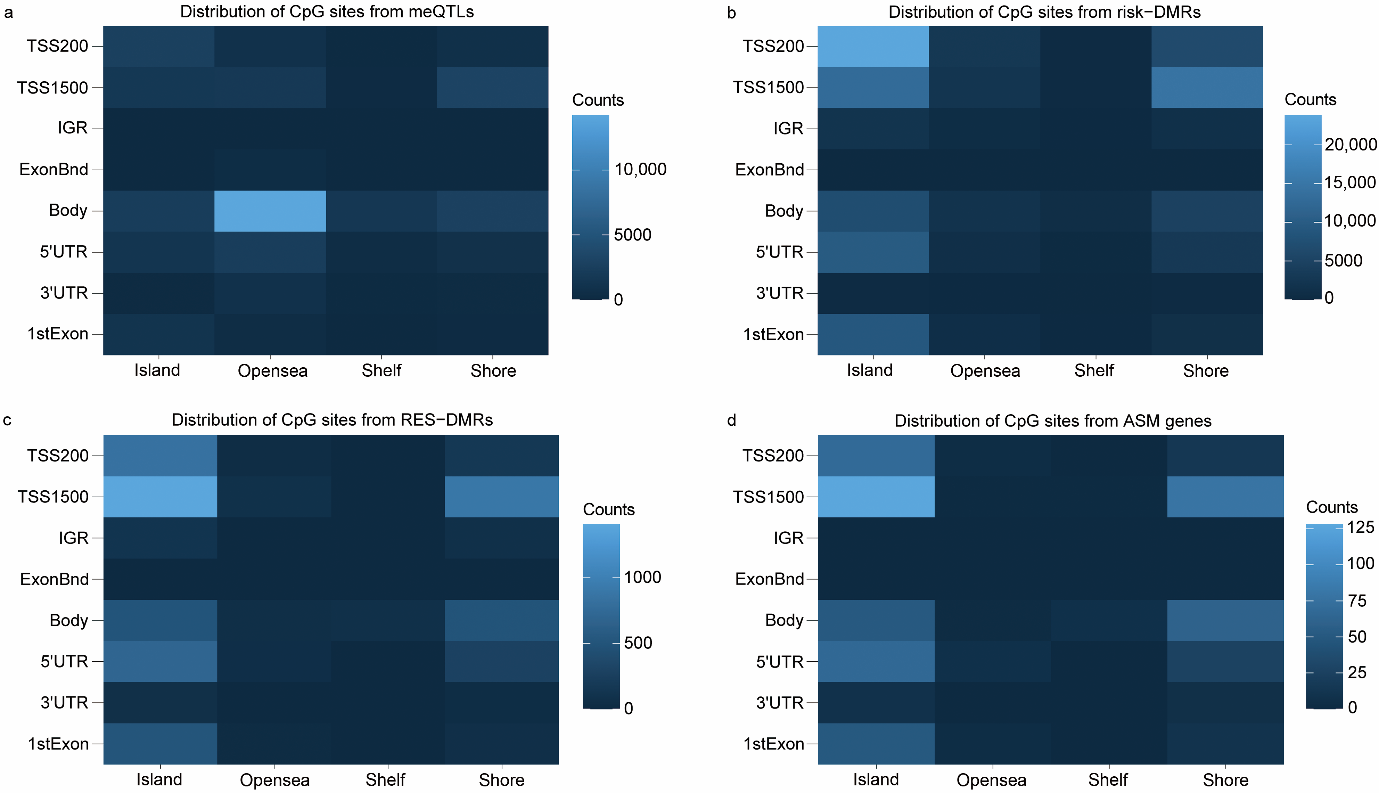


1. Heatmap for the distribution of CpG sites from meQTLs, risk-DMRs, RES-DMRs, and ASM genes. **a-d** Illustrated the distribution of CpG sites identified in meQTLs, risk-DMRs, RES-DMRs, and ASM genes, respectively. TSS transcription start site, IGR intergenic region, ExonBnd exon boundary, meQTL methylation quantitative trait loci, DMR differentially methylated region, RES response, ASM allele-specific methylated


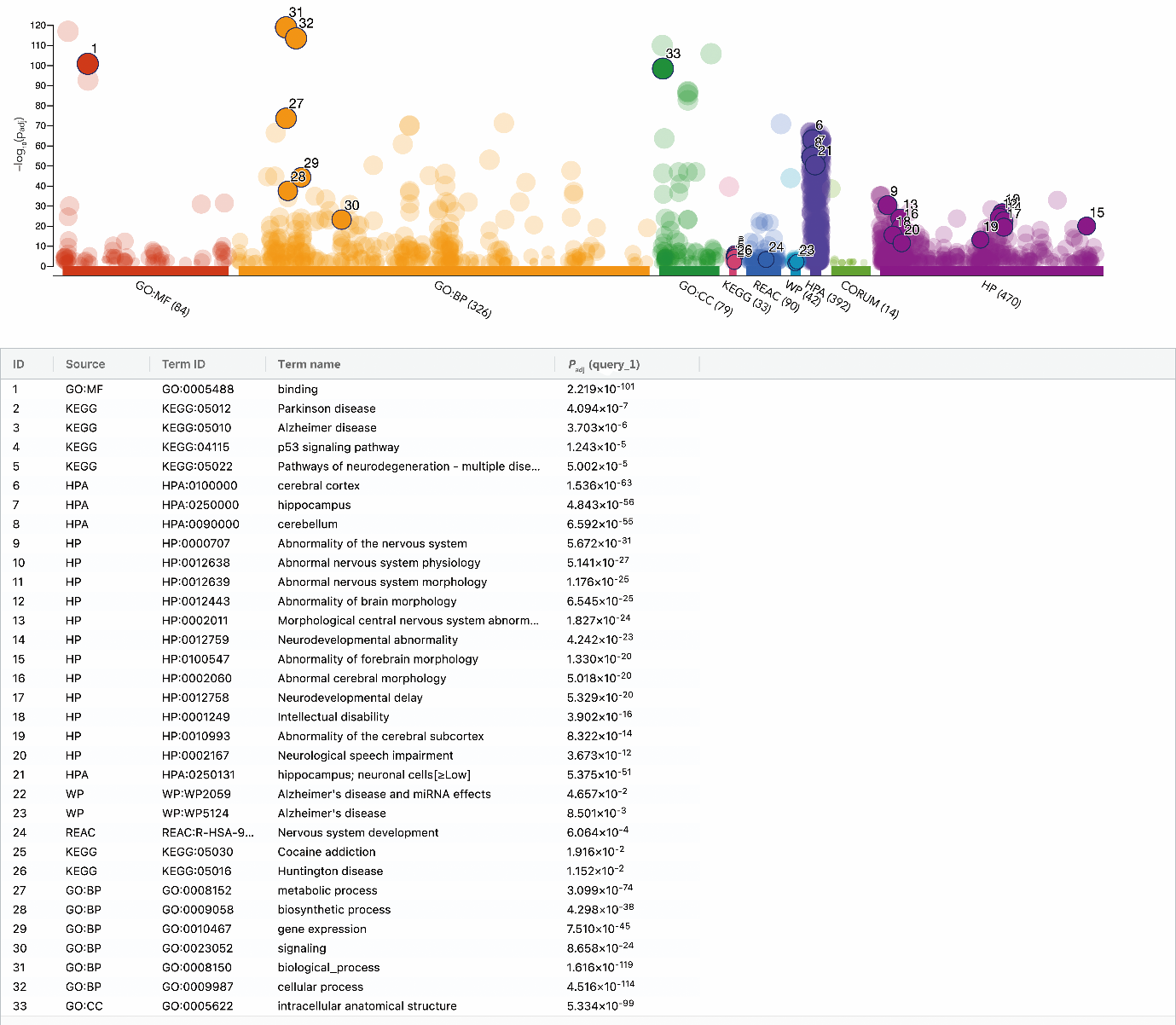


1. Enrichment analysis of gene ontology and biological pathways for ASM genes was conducted by the online tool g:Profiler in nine parts including GO:MF, GO:BP, GO:CC, KEGG, REAC, WP, HPA, CORUM, and HP). A total of 33 relevant items were shown in plot. The full enrichment result can be found in Table S4. GO gene ontology, ASM allele specific methylated, GO:MF GO molecular function, GO:BP GO biological process, GO:CC GO cellular component, KEGG kyoto encyclopedia of genes and genomes, REAC reactome, WP wikipathways, HPA human protein atlas, CORUM comprehensive resource of mammalian protein complexes, HP human phenotype ontology


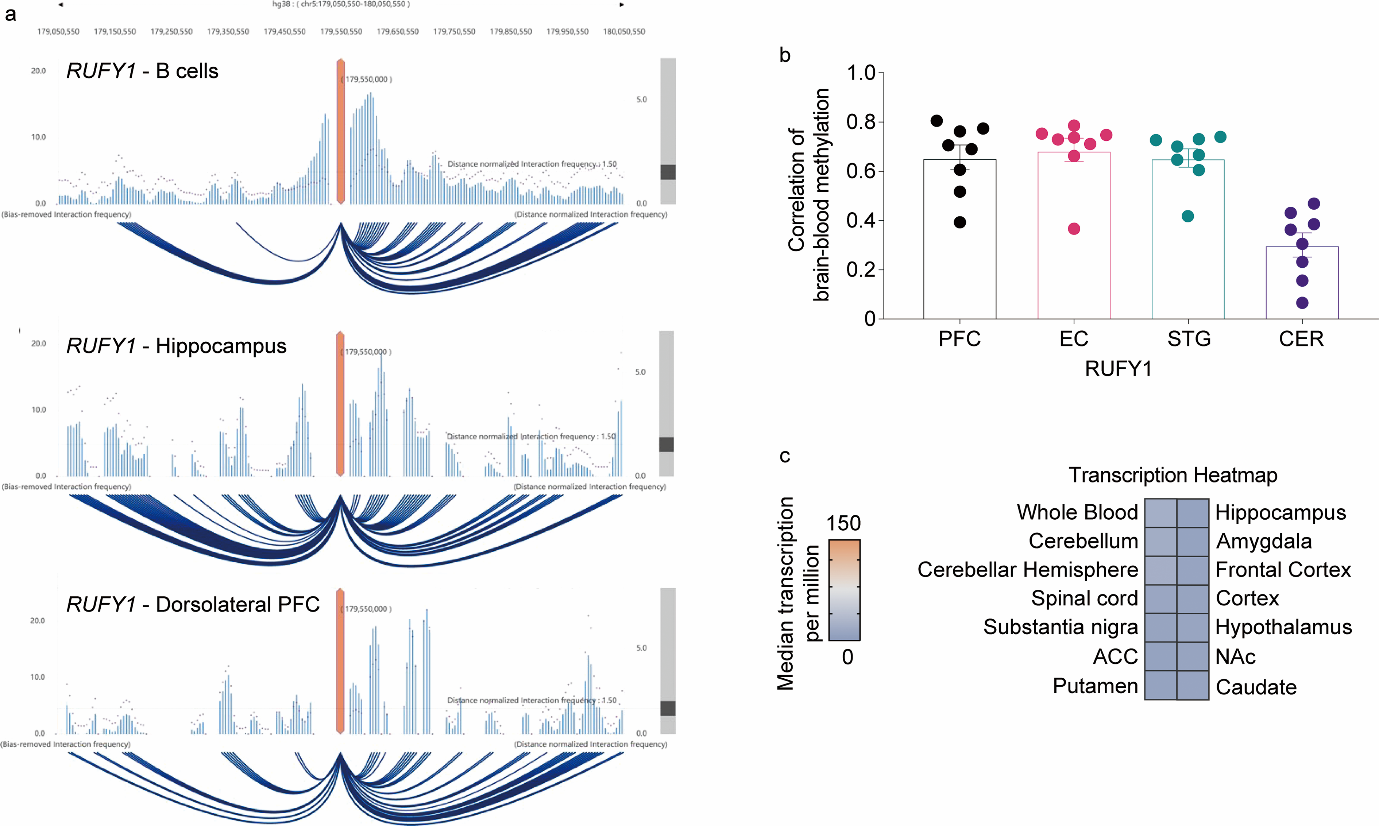


1. Comparison between peripheral blood and brain tissues in transcription, methylation, and chromatin interaction of *RUFY1*. **a** 3D-Genome Interaction Viewer & database showed the captured chromatin interactions of *RUFY1* were abundant and comparative in B cells, hippocampus and dorsolateral prefrontal cortex. **b** The methylation levels of eight probes from *RUFY1* in peripheral blood and brain regions including PFC, EC, STG, and CER were highly correlated. **c** The transcription level of *RUFY1* was in consistency among peripheral blood and brain tissues. PFC prefrontal cortex, EC entorhinal cortex, STG superior temporal gyrus, CER cerebellum, ACC anterior cingulate cortex, NAc nucleus accumbens, RUFY1 RUN and FYVE domain containing 1


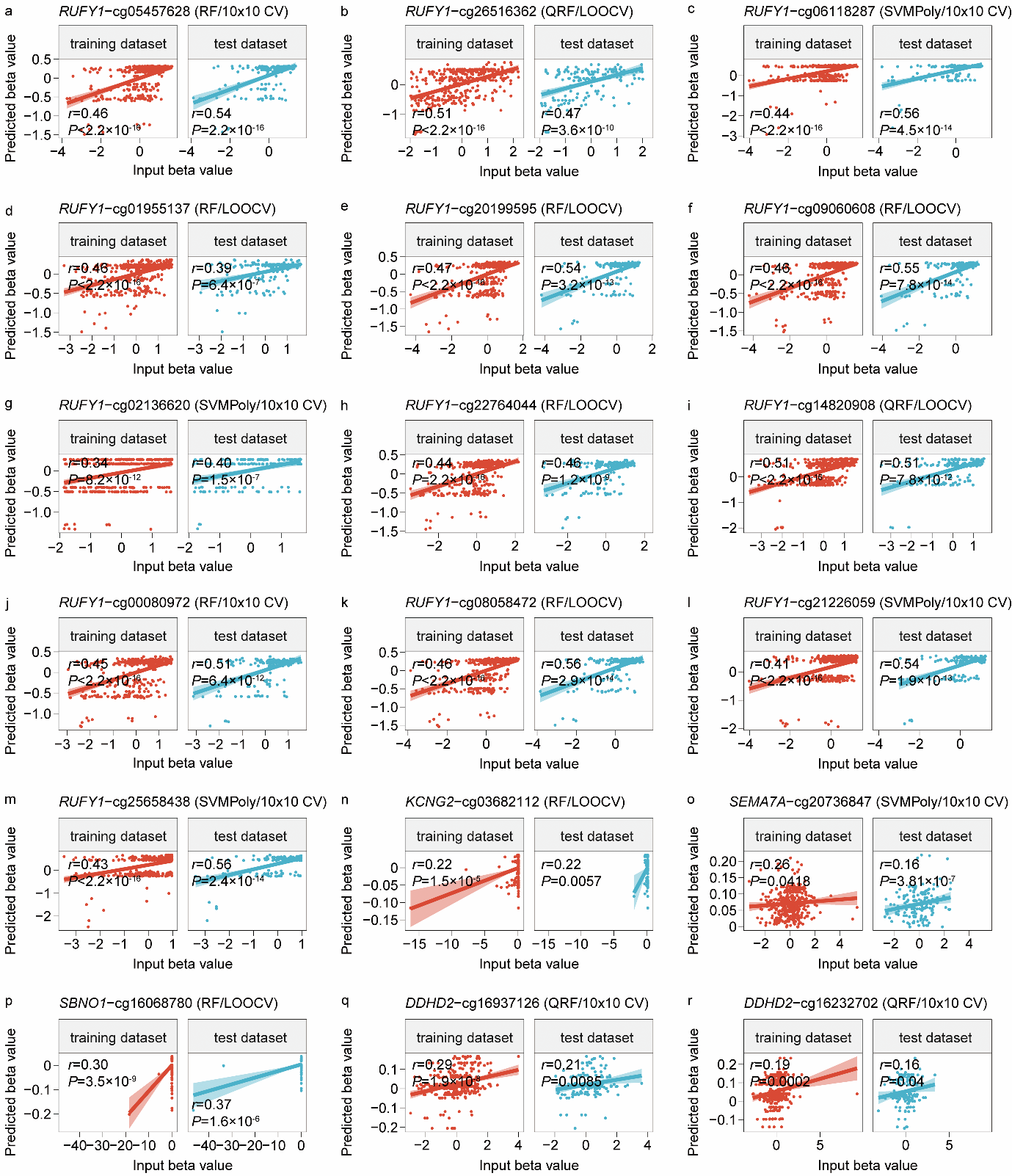


**Fig.S11** Performance of the optimal proxyDNAm models. The 18 scatter plots illustrated the performance of the optimal proxyDNAm model for each CpG site. Algorithms (e.g., RF, SVMPoly and QRF) and resampling methods for cross-validation (e.g., 10×10CV and LOOCV) were described within the brackets. RF random forest, SVMPoly support vector machine polynomial kernel, QRF, quantile random forest, CV cross-validation, LOOCV leave-one-out cross-validation, RUFY1 RUN and FYVE domain containing 1, KCNG2 potassium voltage-gated channel modifier subfamily G member 2, SEMA7A Semaphorin 7A, SBNO1 strawberry notch homolog 1, DDHD2 DDHD domain containing 2
